# Probing the donor strength of yldiide ligands: synthesis, structure and reactivity of rhodium complexes with a PC_ylide_N pincer ligand†

**DOI:** 10.1039/d2sc06759e

**Published:** 2023-03-13

**Authors:** Sébastien Lapointe, Prakash Duari, Viktoria H. Gessner

**Affiliations:** a Faculty of Chemistry and Biochemistry, Chair of Inorganic Chemistry II, Ruhr University Bochum Universitätsstr. 150 44801 Bochum Germany viktoria.gessner@rub.de

## Abstract

Control of the metal ligand interaction by changes in the ligand protonation state is vital to many catalytic transformations based on metal–ligand cooperativity. Herein, we report on the coordination chemistry of a new PC_y_(H)N pincer ligand with a central ylide as donor site, which through deprotonation to the corresponding yldiide changes from a neutral L_3_-type ligand to an anionic L_2_X-type PC_Y_N ligand. The isolation of a series of rhodium complexes showed that the strong donor ability of the neutral ylide PC_Y_(H)N is further increased upon deprotonation, as evidenced by one of the lowest reported CO stretching frequencies in complex [(PC_Y_N)Rh(CO)] (2) compared to other known rhodium carbonyl complexes. DFT calculations revealed that the high donor ability mostly results from the antibonding interaction of the p_π_ orbital at the ylide with the d_*xz*_ orbital at rhodium, which enhances the backdonation into the π* orbital of the CO ligand. This unique interaction results in a rather long metal–carbon bond, but still a strong activation of the CO ligand in order to minimize repulsion between the filled orbitals at the rhodium and the ylide ligand. Accordingly, CO by phosphine replacement leads to a strong deviation from the square-planar geometry in the analogous phosphine complexes [(PC_Y_N)Rh(PR_3_)] and an unusual reactivity with small alkyl halides, which upon oxidative addition add to the CO ligand, before inserting into the P–C bond in the pincer ligand. These results demonstrate the unique donor strength of yldiide ligands and their potential in the activation of strong bonds.

## Abstract 2

A series of rhodium carbonyl complexes with a PC_y_(H)N pincer ligand featuring a central ylide as donor site has been isolated to quantify the donor strength of yldiides in comparison to related ligands.

## Graphical abstract

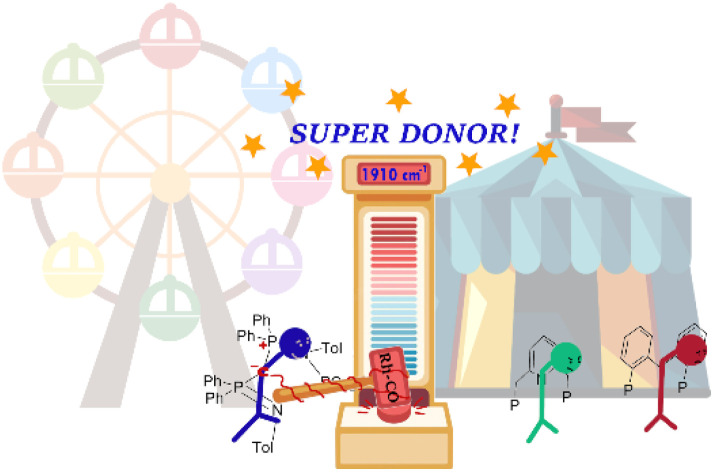

## Introduction

**Fig. 1:**
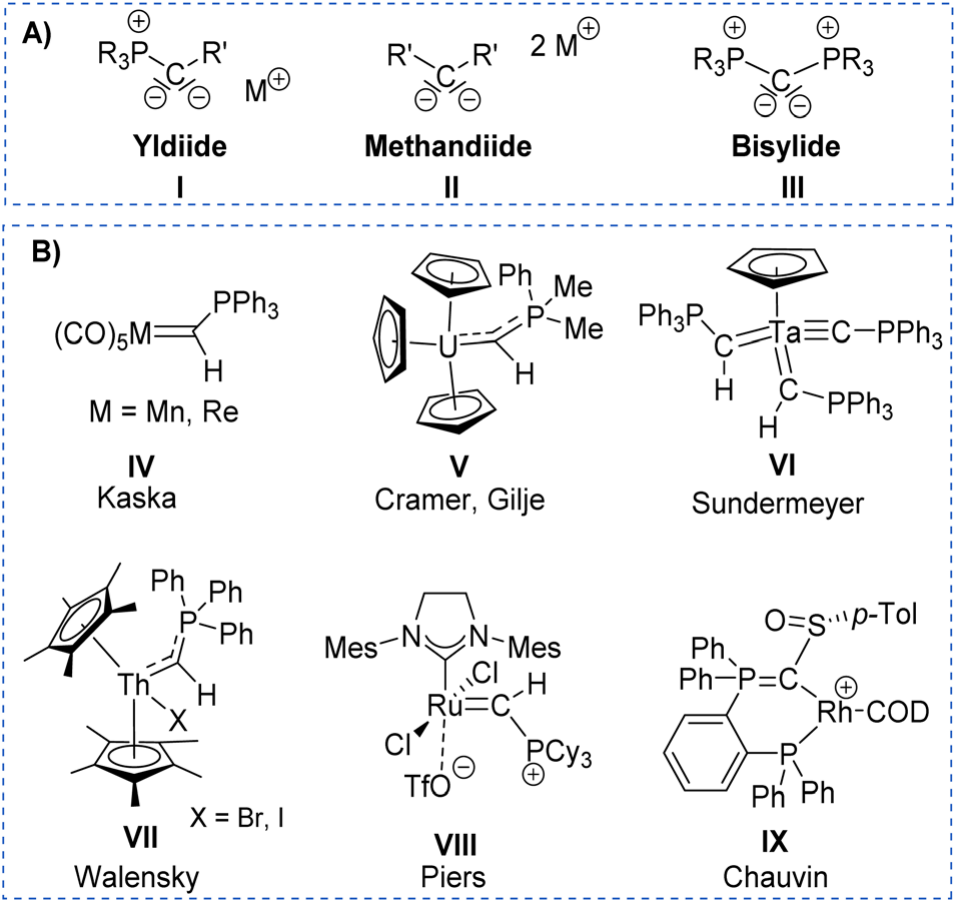
(A) Carbon-based ligands with two lone pairs at carbon and (B) Literature examples of metal yldiide complexes IV–VIII.

Despite ylides being known and applied in organic synthesis for over 100 years,^1^ the chemistry of their α-metalated congeners, the yldiides (I, Fig. 1), is still only little explored and particularly their coordination chemistry towards the d- and f-block elements remains almost untapped.^2^ Yldiide ligands are unique ligands that exhibit unusual bonding patterns and strong electron-donating abilities, which make them attractive as ligand systems to stabilize unusual oxidation states and metal-carbon interactions which might open new reactivity patterns important to catalytic transformations as well as small-molecule activations. Due to the σ- and π-donor abilities of these ligands, even the formation of unusual metal-carbon double bonds to give rise to phosphoniocarbene complexes is possible. However, the formation of these complexes has not systematically been explored and their reactivity is almost unknown. This is surprising giving their relation to methandiides (II)^3^ and bisylides (III),^4^ which have intensively been studied in the past years^5^ and shown to enable new reactivities including bond activations *via* metal ligand cooperativity involving the M=C bond^6^ and multi-metal catalysis.^7^

The first synthesis of a metal yldiide complex was reported by Kaska in 1974.^8^ Albeit no structure analysis was reported at that time, the isolation of the complexes IV obtained from the reaction of manganese(i) and rhenium(i) carbonyl complexes with two equiv. of methylenetriphenylphosphorane was confirmed by IR and NMR spectroscopic data (See Fig. 1). Surprisingly, the first crystallographic proof could be provided by Cramer and Gilje with an actinide metal through isolation of, uranium complex V.^9,10^ Further examples were reported in the following years by Sundermeyer with tantalum and tungsten yldiide complexes such as VI bearing a phosphonium methylide ligand formed by deprotonation of the corresponding ylide in the coordination sphere of the metal.^11,12^ A similar strategy was employed by Walensky and Chen to access the first thorium yldiide VII and scandium/lutetium complexes, respectively,^13^ whereas the analogue to Grubbs catalyst VIII with a phosphoniocarbene ligand reported by Piers was prepared from a carbide complex by addition of tricyclohexylphosphine.^14^ Another notable example includes the work of Chauvin that explored the bonding situation and nature of the cationic rhodium yldiide complex IX.^15^

Our group has recently reported on the isolation of s-block metal yldiides to access low-valent and cationic complexes of the p-block elements.^16^ These applications in main group chemistry impressively demonstrated the potential of this class of ligands to stabilise unique bonding situations thus enabling unusual electronic properties and reactivity patterns.^17^ For example, bond activation reactions repeatedly proceeded *via* a bifunctional mechanism with active participation of the ylidic carbon center in the bond cleavage process.^18^ Analogous studies with transition metals are yet unknown, but highly desirable for the development of novel catalytic transformations. Previous findings with the main group element compounds have shown that the stability of the metal-ylide bonding would benefit from further coordination sites that prevent elimination of the ylide from the metal sphere after the activation step.^19^ Therefore, we addressed the synthesis of a pincer-type ligand framework with a central yldiide donor site to obtain stable transition metal complexes. Here, we report the facile formation of such an PC_Y_N ligand with a central ylide donor site. This ligand allowed the preparation of rhodium complexes which differ in the protonation state of the ligand (ylide PC_y_(H)N *versus* yldiide PC_y_N) and thus allowed a systematic comparison of the donor properties of an ylide *versus* an yldiide donor. Furthermore, we demonstrate that the strong donor properties of the yldiide ligand leads to a strong activation of CO enabling its alkylation and insertion into the ligand backbone.

## Results and discussion

### Synthesis of ligands L1–Li and L2

**Scheme 1:**
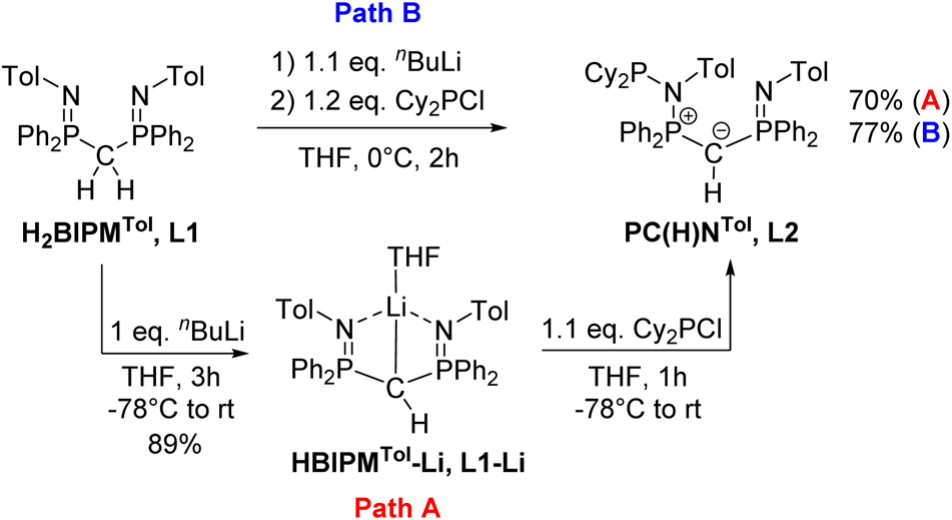
Preparation of ligands L2 from H2BIPM^Tol^ (L1) using two different synthetic pathways.

Previous studies on the bis(iminophosphoranyl)methane ligand H_2_BIPM^Tol^ (L1) and related ligands have shown that their metallated derivatives may undergo substitution either at one of the nitrogen or at the methanide carbon atom.^20*a–f*,21,22^ We assumed that this reactivity may be used for the convenient synthesis of an ylide ligand with an aminophosphonium group through substitution at the nitrogen atom. Indeed, phosphorylation of H_2_BIPM^Tol^ (L1)^23^ to ligand L2 (PC_Y_(H)N) (Scheme 1 and the ESI†) was accomplished either *via* a step-wise or a one-pot reaction protocol: the first pathway involves the deprotonation of L1 with 1 equivalent of *n*-butyllithium and isolation of HBIPM^Tol^-Li, (L1–Li), followed by reaction with 1.1 equiv. of Cy_2_PCl at −78 °C to afford L2 in an overall yield of 70%. Alternatively, L1 is deprotonated with *n*-butyllithium to L1–Li*in situ* and subsequently reacted with Cy_2_PCl to form L2 in 77% yield. This pathway gives the product in overall higher yields despite the formation of the N-protonated HBIPM^Tol^ ligand as an impurity, which is removed through multiple washings with pentanes or hexanes. L2 is partially soluble in pentane or hexanes, and stable in solid state under inert atmosphere, but decomposes over time as a solution in the presence of air.

The ^31^P{^1^H} NMR features of L2 are highly diagnostic and indicative for all complexes derived from this ligand. Whereas L1 and L1–Li feature only a single signal in THF-*d*_8_ at −4.15 ppm and 15.1 ppm, respectively, the loss of symmetry in ligand L2 leads to three distinct signals for the inequivalent phosphorous atoms. The most high-field shifted signal for phosphorus atom P3 (assignment from the solid-state structure, Fig. 3) appears as a doublet at −0.82 ppm (^2^*J*_P3P2_ = 33.1 Hz), the phosphonium atom P2 as a doublet of doublet at 41.6 ppm (^2^*J*_P2P1_ = 79.3 Hz; ^2^*J*_P2P3_ = 33.1 Hz) and the P(iii) atom (P1) as a doublet at 63.4 ppm (^2^*J*_P1P2_ = 79.3 Hz). The ^1^H NMR spectrum also reflects the breaking of symmetry *via* the doubling of signals in the aromatic region. While the signal for the hydrogen on the central carbon resonates as a doublet at 1.82 ppm (*J*_PH_ = 7.3 Hz), the signal of the central ylidic carbon appears as a doublet of doublet of doublet at 14.8 ppm (*J*_PC_ = 136.3, 109.8, 7.9 Hz) in ^13^C{^1^H} NMR spectrum.

**Fig. 2:**
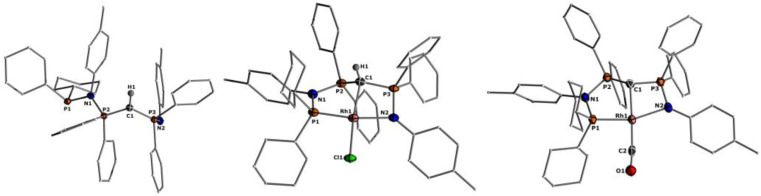
Molecular structures of ligand L2 (left), complex 1 (middle), and 2 (right). Ellipsoids are shown at 50% probability. Solvent molecules, minor disordered components as well as hydrogen atoms, except for H1 omitted for clarity. Only one of the two molecules present in the asymmetric unit is shown for complex 2. Selected bond angles and distances are given in Table 1.

The solid-state structure of L2 (Fig. 2) reflects the differences in the nature of the P–C and P–N bonds flanking the central carbon atom C1. Whereas the P3–C1 bond length of 1.734(3)Å is in the range of a typical single bond, the bond length of P2–C1 of 1.688(3) Å is considerably shorter due to its ylidic character (Table 1). Likewise, the ylidic P3–N2 bond of the iminophosphoryl group is shorter than the P2–N1 distance. To further understand the nature of the P–C bonds, we have calculated the natural charge of the involved atoms (Table S9 in ESI†). The natural charge of P2 (1.87*e*) is slightly higher than the charge of P3 (1.83*e*), thus giving rise to a slightly stronger attractive interactions in the P–C–P linkage with the negatively charged carbon atom (−1.43*e*).

**Table 1:** Bond distances (A) and angles (deg) for complexes 1 and 2

	Rh1–P1 [Å]	Rh1–N2 [Å]	C1–Rh1 [Å]	Rh1-L [Å]	P2–C1 [Å]	P3–C1 [Å]	P2–N1 [Å]	P3–N2 [Å]	P2–C1–P3 [deg]	C1–Rh1-L [deg]	P1–Rh1–N2 [deg]	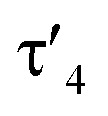 a
L2	—	—	—	—	1.688(3)	1.733(3)	1.754(2)	1.593(2)	124.8(2)	—	—	—
1^b^	2.136(1)	2.138(1)	2.118(2)	2.376(1)	1.732(2)	1.783(2)	1.679(1)	1.593(1)	125.8(1)	173.0(1)	162.2(1)	0.141
2^c^	2.191(1)	2.137(2)	2.100(2)	1.828(3)	1.671(2)	1.695(2)	1.693(2)	1.623(2)	135.13(15)	178.0(1)	159.8(1)	0.099

a Geometrical index 
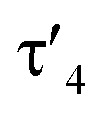
 for the rhodium centers.^24^

b Data are listed for the main disordered component.

c There are two complexes in the asymmetric unit; data are tabulated for the first one.

### Synthesis and characterization of a rhodium ylide and yldiide complex

**Scheme 2:**
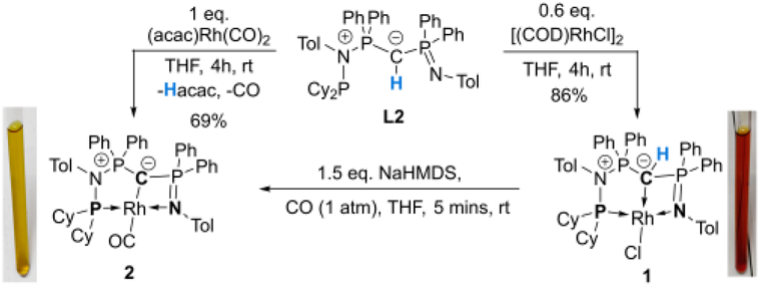
Preparation of rhodium chloride and rhodium carbonyl complexes 1 and 2.

To probe the ability of L2 to serve as pincer ligand, the ligand was metalated using commercially available rhodium precursors. In the presence of 0.6 equiv. of [(COD)RhCl]_2_ (COD = 1,5-cyclooctadiene) in THF at room temperature complex 1 was formed as an orange-brown powder in a high yield of 86% (Scheme 2). Complex 1 is sensitive to air or moisture and decomposes over time under air in solution. In a similar way, the reaction of 1 equiv. of [(acac)Rh(CO)_2_] (acac = acetylacetonate) with L2 produces the rhodium complex 2 as a bright yellow powder in 69% yield. Here, the acac ligand acts as a base, deprotonating the ylidic carbon atom to the yldiide. In contrast to 1, the yldiide complex 2 is stable under air in solid state and in solution. Interestingly, when complex 1 is reacted with 1.5 eq. of NaHMDS under an atmosphere of CO, complete conversion of 1 to 2 was observed by ^31^P{^1^H} NMR spectroscopic studies (Fig. S52†) and also confirmed by the colour change from orange to yellow.

**Fig. 3:**
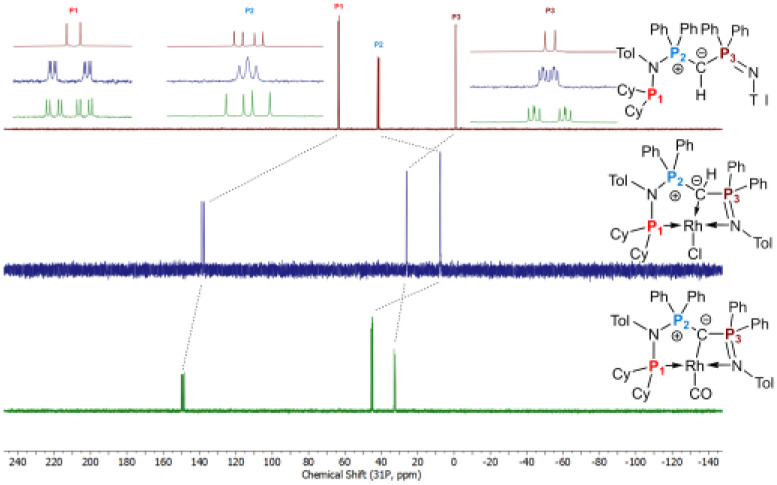
Stacked ^31^P{^1^H} NMR spectra of ligand L2 (top, burgundy), complex 1 (middle, blue), and 2 (bottom, green). Zoomed portions at the top are the signals for each phosphorus atoms aligned to each other for better comparison.

The formation of the complexes 1 and 2 can be easily followed by the changes of the chemical shift in the ^31^P{^1^H} NMR spectrum as well as the appearance of the coupling to the rhodium center (Fig. 3). For example, The signal for P1 appears at 138.1 ppm as a ddd (^1^*J*_RhP1_ = 202.4 Hz, ^2^*J*_P1P2_ = 29.8 Hz, and ^3^*J*_P1P3_ = 9.9 Hz) with a shift of +74.7 ppm *vs.*L2, whereas P2 is highly upfield shifted (*Δ* = −34.1 ppm) compared to L2 and appears as a triplet at 7.5 ppm due to the similar coupling constants with Rh and P1. Finally, the signal of P3 resonates downfield shifted (*Δ* = +26.4 ppm) at 26.1 ppm as a ddd (^2^*J*_RhP3_ = 36.2 Hz, ^2^*J*_P3P2_ = 13.8 Hz, ^2^*J*_P3P1_ = 10.2 Hz). Complex 2 exhibits significant differences in the coupling patterns and NMR shifts. The signals for P1–P3 are downfield shifted compared to complex 1 and ligand L2, which might be explained by the more electron-withdrawing nature of the CO ligand in 2. Unfortunately, the signals for the ylidic and carbonyl carbon atom could not be detected in the ^13^C{^1^H} NMR spectrum.

**Fig. 4:**
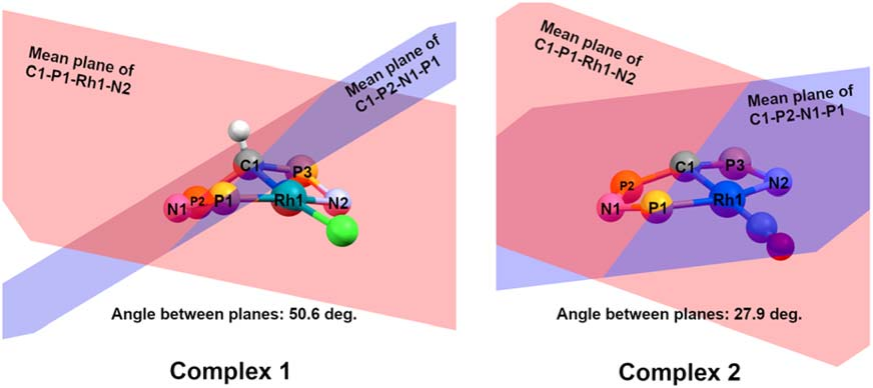
Comparison of the buckling angles in complexes 1 (left) and 2 (right). Only selected atoms are shown for clarity. The red plane is formed from the mean between the atoms C1–P1–Rh1–N2, the blue plane from the mean between the atoms P1–N2–P2–C1. The buckling angle is the angle between both mean planes.

Important insights into the coordination properties of the ligand in its two different protonation states were obtained from the structures of the complexes 1 and 2 in the solid state (Fig. 2). With a 
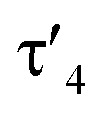
 parameter of 0.141 the Rh center in 1 adopts a geometry in between that of an ideal square planar (
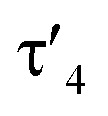
 = 0) and ideal see-saw (
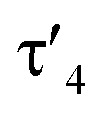
 = 0.24) structure.^24^ In contrast, complex 2 features a distorted square-planar geometry with a 
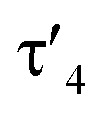
 value of 0.099.^12^ To our surprise, the Rh–C1 bond lengths in 1 and 2 don't reflect the expected change in electronic nature of the metal carbon interaction, *i.e.* Rh-ylide *versus* Rh-yldiide bonding.^15^ Only a small difference in the bond lengths (2.116(2) for 1*vs.* 2.100(2) for 2) is observed, despite the propensity of the yldiide to form an additional π-bond. This can presumably be explained by the pyramidalization of the carbon atom in both the ylide and the yldiide ligand. This pyramidalization is less pronounced in 2, but still prevents an efficient π-interaction with the metal center. This difference can be visualized by comparing the buckling angle in the 5-membered ring (P1–N1–P2–C1–Rh1) for both complexes (Fig. 4). Complex 1 has a buckling angle of 50.6°, while complex 2 features a buckling angle of 27.9°, thus resulting in a different orbital overlap in both systems, but still no efficient π-bonding. The difference in geometries also becomes apparent when comparing the P2–C1 and P3–C1 bond lengths in both complexes. These bonds are longer in complex 1 than in complex 2, which is in line with an increased electron density at the central carbon atom in 2 because of the deprotonation of the ylide. Overall, the structural parameters indicate that the higher charge of the yldiide ligand is both delocalized within the ligand backbone as well as slightly towards the metal centre, thus arguing for a stronger donor ability of the ligand.

### Electronic structure and bonding situation

**Fig. 5:**
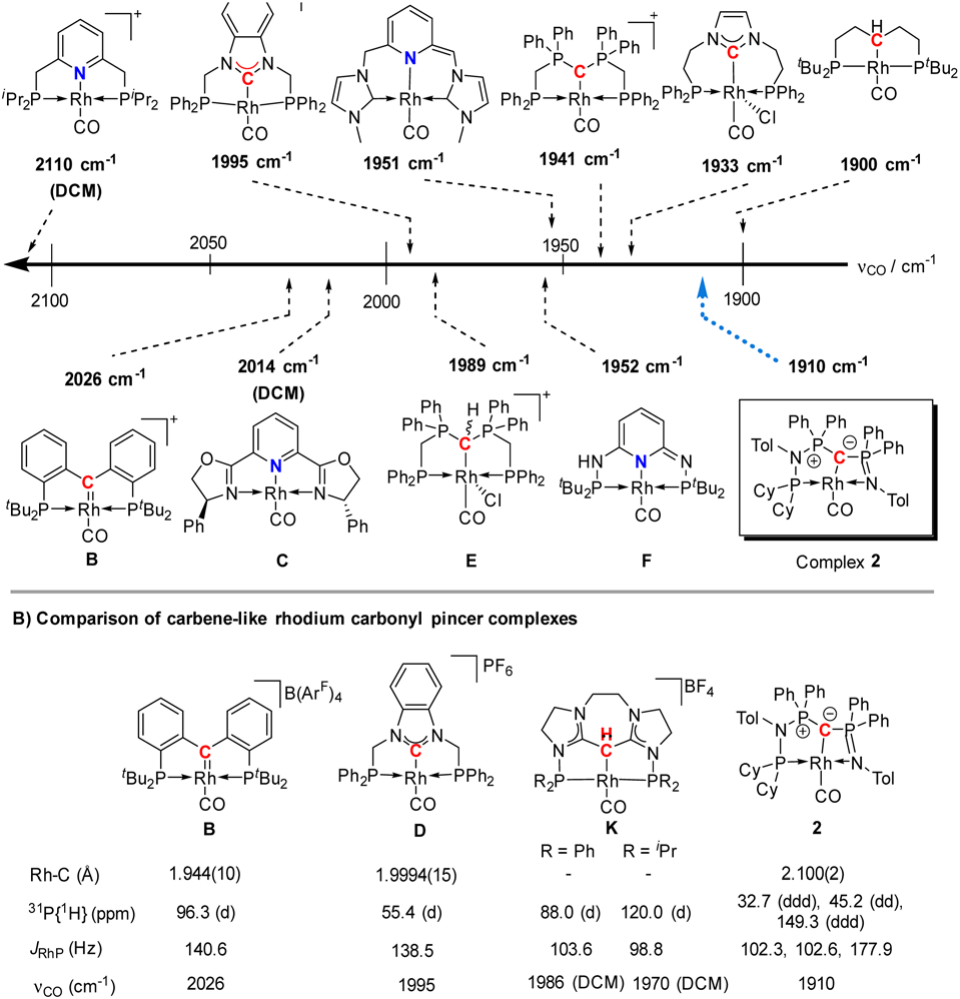
(A) Carbonyl stretching frequency of different pincer-based rhodium complexes A–J and complex 2,^25–28,30–32,36^ and (B) comparison of different properties of selected carbenoid rhodium carbonyl pincer complexes B, D, K, and complex 2. Unless noted, the carbonyl stretching frequencies were reported in the solid state.

The highly electron-donating nature of the yldiide ligand attached to the rhodium center is confirmed by IR analysis of the CO stretching vibration of complex 2, showing a frequency of 1910 cm^−1^. This stretching frequency is extremely red shifted. A literature survey in fact showed that it is – to the best of our knowledge – the second lowest frequency from all other reported pincer rhodium carbonyl complexes (Fig. 5A).^25–31^ The lowest frequency of 1900 cm^−1^ was reported by Shaw for the aliphatic PCP rhodium carbonyl complex J.^29^ It becomes apparent, that the nature of the bonding between the Rh and the central atom has an important effect on the CO stretching frequency. Comparison of the cationic complexes A, D and H reflects the weaker donor strength of pyridines compared to carbenes, which itself are weaker donors than the bisylide ligand in H. Although the different charges make a direct comparison difficult, the yldiide ligand again appears to contribute to a further increase in donor strength. Also, the hybridization of the donor carbon site decisively influences the donor properties. Complex E has a *ν*_CO_ 48 cm^−1^ less than the carbene complex H formed by treatment of E with a base.^30^

A comparison (Fig. 5B) of selected carbenoid pincer complexes, including an NHC moiety (D),^32,33^ a “Fischer-like” carbene (B),^34^ a carbodicarbene (K)^35^ and our yldiide ligand 2 shows that complex B has the shortest Rh–C bond length with 1.944(10) Å, but the highest CO stretching frequency (2026 cm^−1^) in this series. In contrast, complex 2 shows the highest donor ability of these complexes with the lowest CO stretching frequency (1910 cm^−1^) but the longest Rh–C bond length (2.100(2) Å).^36^ These trends can be rationalized by the different bonding situations between these ligands and the metal center: as such, the Fischer carbene complex forms the strongest M–C_carbene_ bond due to the synergistic bonding situation with the σ-donating and π-accepting carbene ligand. This bonding interaction prevents charge accumulation at the metal and hence leads to a higher stretching frequency. In contrast, the σ- and π-donating properties of the carbodicarbene and yldiide ligand lead to lower stretching frequencies due to the increased electron transfer towards the metal, but a weaker metal carbon interaction owing to the missing backdonation.

**Fig. 6:**
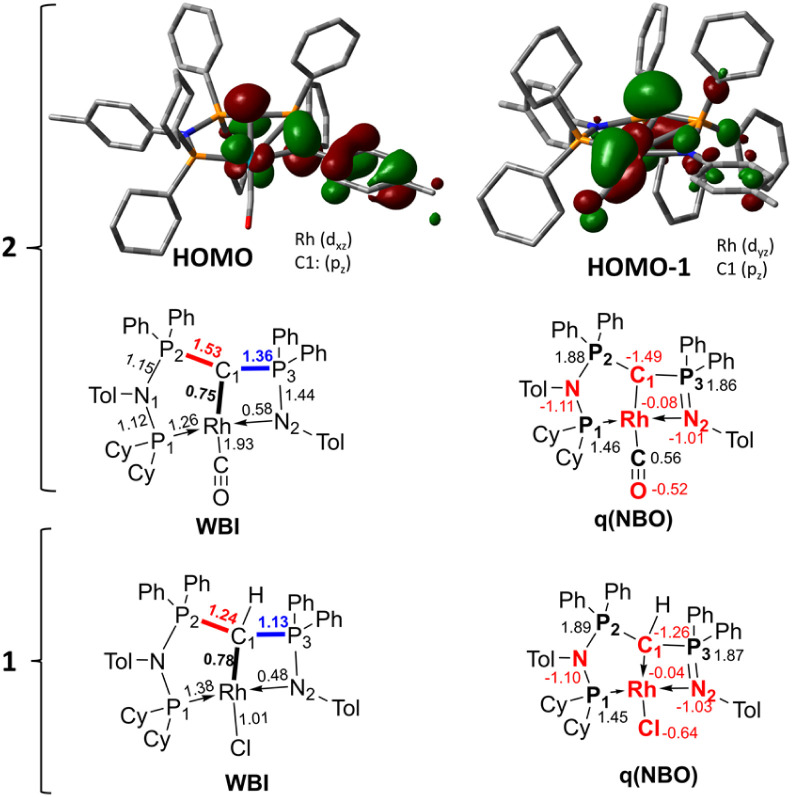
Display of the two highest occupied molecular orbitals of complex 2 and Wiberg bond indices (WBI) and NBO charges in 1_calc_ and 2_calc_.

To better understand the properties of complex 2 computational studies on the PW6B95-D3/def2tzvp^37,38^ level of theory were performed. The electronic structure can be rationalized by the two highest molecular orbitals (HOMO) (Fig. 6). The HOMO of 2 is mostly based on the imino group and represents an antibonding interaction with the Rh centre (d_*xz*_ orbital, Rh contribution: 15.8%), with only a low contribution from C1 (9.6%). In contrast, the HOMO-1 represents the C1–Rh π interaction between the p_π_ orbital at the ylide and the d_*yz*_ orbital at the metal as well as the π* orbital of the CO ligand. Most remarkably, this interaction is antibonding in nature and thus leads to no significant π bonding character of the carbon metal bond. This is reflected in the rather long Rh–C distance observed in the crystal structure and the high negative charge residing at the C1 carbon atom (*q* = −1.49*e*) (See the ESI† for further representations of the orbitals, charges and WBIs in 1_calc_ and 2_calc_). Similar bonding interactions have been observed with methandiide ligands in combination with late transition metals, which resulted in a high reactivity of the M–C bond and enabled bifunctional bond activation reactions.^39,40^ Presumably, this antibonding character of the ligand–metal π interaction is responsible for the pronounced weakening of the C–O bond, as evidenced by the very low stretching frequency. To minimize the repulsive interaction between the two filled orbitals at the carbon (p_π_) and at the metal center (x_*yz*_), the back-bonding into the π* orbital of the CO ligand is strengthened. This ultimately results in a longer C–O bond, but also causes a stronger interaction between the Rh and C atom relative to the bonding found with the protonated ligand (*vide infra*).

### Reactivity studies of complexes 2

**Scheme 3:**
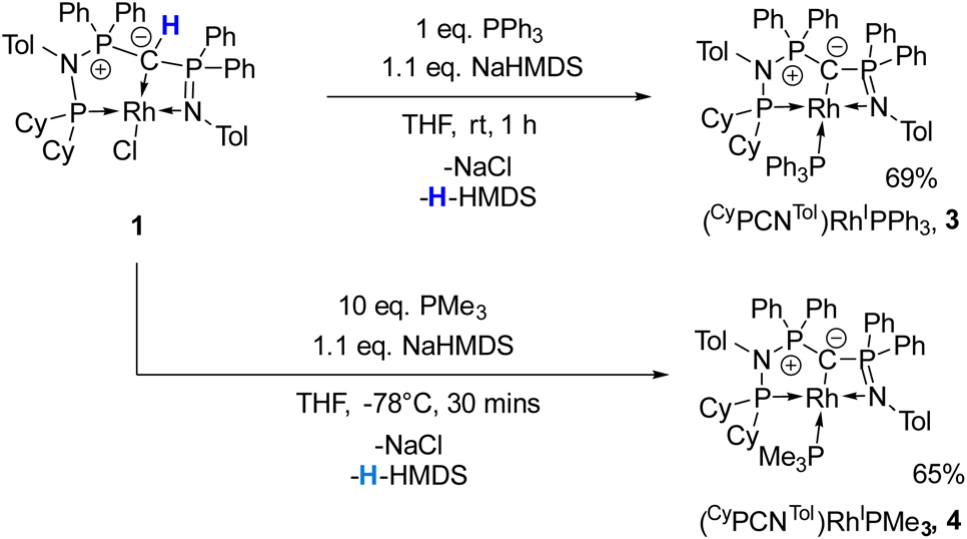
Reactivity of complex 1 with NaHMDS and PR_3_ (R = Ph, Me) to form complexes 3, and 4.

**Fig. 7:**
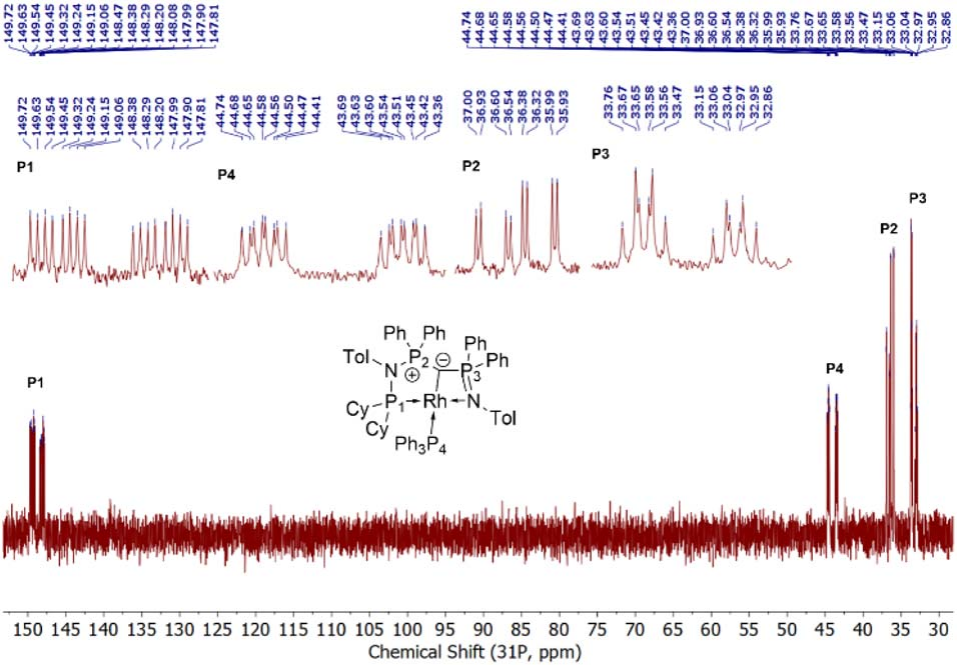
^31^P{^1^H} NMR of complex 3 (162 MHz, C_6_D_6_). Zoomed regions to show coupling patterns.

Given the unique electronic structure of yldiide complex 2 we next focussed on its reactivity. Initially, we were interested in the impact of other co-ligands than CO on the C–M interactions. Since phosphines are weaker acceptors than CO, we assumed that phosphine complexes analogous to 2 would exhibit an even stronger polarization of the C–Rh bond, since the more electron-rich metal center should be less able to accept electron density from the yldiide ligand. Whereas simple CO for phosphine (PPh_3_ or PMe_3_) exchange was unsuccessful with complex 2, reaction of 1 in the presence of 1.1 eq. NaHMDS and 1 eq. PPh_3_ led to the desired yldiide phosphine complex 3 as brown powder in an approx. 69% yield (Scheme 3). The very low solubility of 3 hampered efforts to fully purify the complex. However, we were able to confirm the connectivity in complex 3 by XRD analysis, (the quality of the crystals was too low for a detailed analysis), and its very characteristic ^31^P{^1^H} NMR spectrum, showing four phosphorus signals with a distinct coupling pattern (Fig. 7 and the ESI†).

**Fig. 8:**
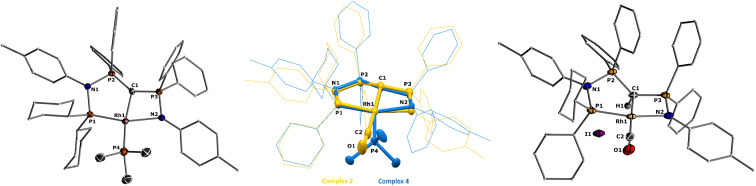
Solid-state molecular representation of complex 4 (left), overlay with complex 2 (middle), and structure of complex 5. Ellipsoids are shown at 50% probability level. Hydrogen atoms are omitted for clarity. Only one of the two molecules present in the asymmetric unit is shown for complex 4 and 5. Complex 2 in the overlay is in yellow, while complex 4 is in blue. Overlay RMS = 0.173.

The same reactivity of complex 1 was observed with an excess of PMe_3_ at low temperatures. According to ^31^P NMR spectroscopy complex 4 is formed in good yields, but revealed to be less stable than complex 3, showing signs of degradation *via* loss of phosphine under prolonged vacuum. Nonetheless, the complex can be purified by crystallization *via* vapor diffusion of pentane into a concentrated benzene solution, to afford dark red crystals suitable for XRD analysis. Reacting complex 2 under the same conditions but at room temperature led to the formation of further by-products, which complicated the isolation and purification of the product. The instability of the complex already demonstrates the weak bonding of the phosphine ligand in *trans*-position to the strongly donating yldiide. This electronic mismatch is also reflected in the molecular structures (Fig. 8 and Table 2). As such, coordination of the PMe_3_ group dramatically changes the geometry of the complex in comparison to 2, which is especially apparent when comparing the C1–Rh–P4 (169.2°) angle in 4 to the C1–Rh–CO angle in 2 (178.0°). As can be seen by overlapping the two structures (Fig. 8, middle), the phosphine ligand bents out of the metal-yldiide plane, probably to avoid repulsive electronic interactions (see below). This bending leads to a further change in the geometry around C1 towards planarity (buckling from the plane for 4 (10.04°) compared to 2 (27.9°)) and a better orbital interaction between C1 and the metal center. Therefore, the Rh–C distance is – in contrast to our initial expectation – slightly shorter in the phosphine complex than in 2.

**Table 2:** Bond distances (A) and angles (deg) for complexes 3–5

	Rh1–P1 [Å]	Rh1–N2 [Å]	C1–Rh1 [Å]	Rh1-L [Å]	P2–C1 [Å]	P3–C1 [Å]	C1–Rh1-L [deg]	P1–Rh1–N2 [deg]	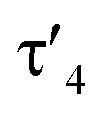 a
3^d^	2.176	2.191	2.059	2.247	1.676	1.655	168.37	158.06	0.204
4^c^	2.175(1)	2.176(2)	2.088(3)	2.239(1)	1.646(3)	1.686(3)	168.6(1)	158.3(1)	0.193
5^b^	2.222(1)	2.143(3)	2.141(3)	1.848(3)	1.737(3)	1.768(3)	176.2(1)	160.3(1)	0.114

a Geometrical index 
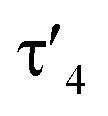
 for the rhodium centers.^24^

b There are two complexes in the asymmetric unit; data are tabulated for the first one.

c Data are listed for the main disordered component.

d Bond lengths and angles are only reported for comparison but are not accurate as they obtained from a connectivity analysis.

It is also interesting to note, that the stronger Rh–yldiide interaction in 4 affects the bonding of the P and N donor sites to the metal. While the Rh–P1 distance is slightly shorter (2.185(1) Å) for 4*vs.*2 (2.191(1) Å), the Rh–N bond is much longer in 4 (2.190(3) Å) than in 2 (2.137(2) Å). This might be the result of a lower shift of electron density from the ylide into the PN moiety (*via* negative hyperconjugation) in 4, thus rendering the nitrogen atom less electron-rich and hence a weaker donor. This is subsequently compensated by the stronger donation of the phosphine P1. The geometry of the PPh_3_ complex 3 is similar to that of 4 (Table 2), and as such we assume that any bonding analysis would be similar for 3.

**Scheme 4:**
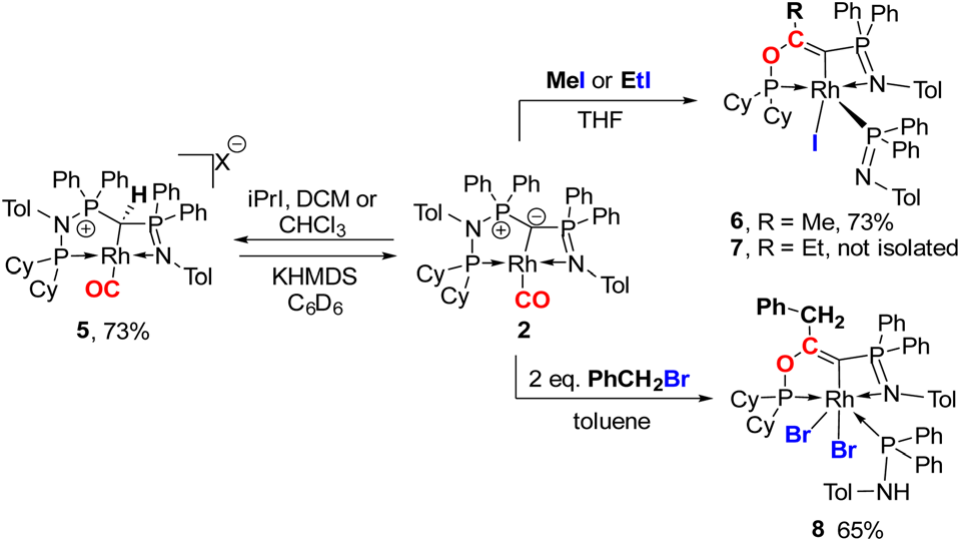
Reactivity of complex 2 with different alkylhalides to form complexes 5–8 (X = I for reaction with iPrI or X = Cl for reaction with DCM or CHCl_3_).

Next, we addressed the reactivity of the M–C bond of 2, particularly focusing on a potential bifunctional behaviour as reported for methandiide complexes with similar electronic structures.^6,39,41^ However, attempts at reacting complex 1 (in the presence of 1.5 equiv. of NaHMDS) or 2 with H_2_, NH_3_, isopropanol, or CO_2_ mostly gave decomposition of the complex, or a mixture of products which could not be identified. Therefore, we turned our attention towards carbon electrophiles to probe whether the complex undergoes traditional oxidative addition reactions at the metal center. The reaction of complex 2 with excessive isopropyl iodide at RT (Scheme 4) led to the slow formation of a new complex and reached full conversion in chloroform after 72 hours (Fig. S53 in ESI† for reaction monitoring) to give the cationic complex 5 in 73% yield. The choice of solvent is important for this reaction. Using THF instead of chloroform only gives 9% under the same conditions. Yellow crystals suitable for XRD analysis were obtained by diffusion of pentane into a concentrated chloroform solution and revealed the formation of the cationic carbonyl complex *via* protonation at the ylidic carbon center. Complex 5 shows three distinct phosphorous signals in the ^31^P{^1^H} NMR spectrum, which appear at 37.0, 50.8 and 144.8 ppm with characteristic coupling patterns. The ^1^H NMR for complex 5 has the C–H signal of the central carbon at 2.1 ppm as a doublet (*J*_RhH_ = 13.1 Hz), while the ^13^C{^1^H} signal for the carbon appears as a doublet at 37.6 ppm (^1^*J*_RhC_ = 24.3 Hz). Moreover, reaction of complex 5 with 1.2 equivalent of KHMDS in THF instantaneously gives back the starting complex 2*via* deprotonation of the central carbon atom and removal of the iodide (Fig. S54 in ESI†).

**Fig. 9:**
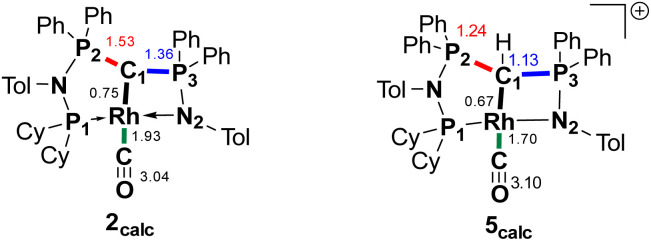
Summary of the Wiberg bond indices in Lowdin orthogonalized basis at selected atoms in complexes 2_calc_ and 5_calc_.

We initially hypothesize that 5 is formed by oxidative addition of isopropyl iodide to the rhodium center, which is followed by the abstraction of a proton from one of the methyl groups by the ylidic carbon atom, releasing propylene and forming a cationic Rh^I^ center. However, also reaction of 2 with phenyl iodide slowly (6 days) forms complex 5 suggesting the involvement of the solvent in the reaction process. Indeed, stirring of complex 2 in chloroform (or in DCM at 60 °C) in the absence of any alkyl or aryl halide led to the formation of 5 within 3 days reaction time (Fig. S59 and S60 in the ESI†). The isolation of 5 provides the unique possibility of comparing the impact of the protonation state of the ligand on the metal carbon bonding. In the molecular structure, complex 5 has a Rh–C1 bond length of 2.144(4) Å which is longer than that of the yldiide carbonyl complex 2 and even slightly longer than that in the chloro complex 1 (Fig. 8 and Table 2). The geometry index of *τ*_4_ = 0.114 for 5 is only slightly higher than in 2, suggesting that the longer Rh–C distance in 5 is the result of the differences in the bonding of the ylide and yldiide ligand and not due to changes in the geometry at the Rh center. Finally, the IR stretching frequency of the CO moiety in 5 appears at 1968 cm^−1^, much higher than in 2 (1910 cm^−1^) and indicative of a much less electron-donating character of the ligand. This comparison clearly demonstrates that depite the pyramidal C atom in 2 and the antibonding Rh–C π-interaction (Fig. 4), there is an additional transfer of electron density from the yldiide ligand to the Rh center. This difference is also reflected in the differences in the Wiberg bond indices in 2_calc_ and 5_calc_ (Fig. 9). As such, the deprotonation to 2 results in an increase of the bond indices of the C1–P bonds, but also of the C1–Rh and the Rh–CO linkage. Thus, the strengthening of the C–Rh bond results in a stronger π-backbonding as indicated by the lower C–O bond index in 2_calc_ and the experimentally observed C–O vibrations.

**Fig. 10:**
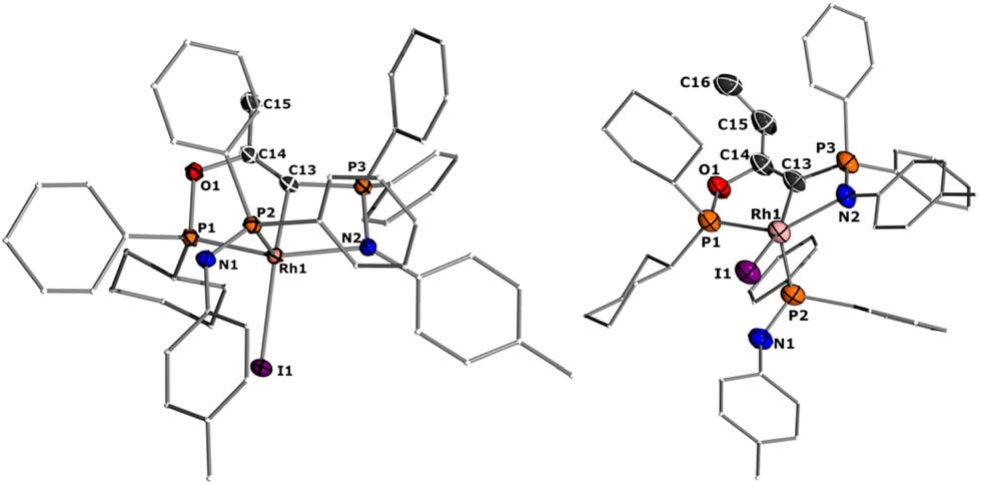
Solid-state structures of complex 6 (left) and 7 (right). Ellipsoids are shown at a 50% probability level. Hydrogens were omitted for clarity. Only 1 of the two molecules in the asymmetric unit is shown for complex 7. Selected bond lengths and angles: 6: Rh1–P1 2.209(1), C13–Rh1 1.9963(19), Rh1–P2 2.278(1), C13–C14 1.331(3), C14–O1 1.393(2), Rh1–N2 2.2435(16), Rh1–I1 2.7006(2), P3–C13 1.752(2). 7 Rh1–P1 2.1983(14), Rh1–C13 2.017(5), C13–C14 1.328(7), C13–O1 1.399(6), Rh1–N2 2.265(4), Rh1–I1 2.7136(5), P3–C13 1.739(5).

Since the formation of 5 indicates no fast conversion with carbon electrophiles (^i^PrI and PhI), we turned our attention towards more reactive reagents. Indeed, treatment of 2 with methyl iodide resulted instantly in a colour change from yellow to deep purple. Performance of the reaction at −30 °C and slowly warming to room temperature resulted in a new species characterized by three signals at 185.5, 39.1 and 33.7 ppm in the ^31^P NMR spectrum, which could be isolated in a 73% yield. XRD analysis (Fig. 10) revealed the product of the reaction to be Rh^III^ complex 6 where the insertion of an acyl moiety^42–45^ into the ligand backbone along with the cleavage of the P1–N1 bond and C1–P2 bond took place. The resulting ligand is no longer an ylide, but a more traditional PCN pincer ligand with a sp^2^-carbon at the central position (C13). The Rh1–C1 bond length is shorter than in complexes 1–5 at only 1.9963(19) Å due to the higher oxidation state of the metal.^46–48^ The C13–C14 bond length amounts to 1.331(3) Å and is thus in the range of a typical double bond. In contrast, the C–O bond from the former carbonyl ligand is considerably elongated (1.164(3) Å for 2*vs.* 1.393(2) Å for 6), indicating a reduction of the triple to the single bond. To the best of our knowledge such a reactivity has not been observed before for any ylide ligand. It is noteworthy that complex 6 is not stable in solution for a longer period, but slowly undergoes intramolecular C–H bond activation of the tolyl group of the Ph_2_PNTol ligand (see ESI† for details).

Interestingly, this reactivity is not unique to methyl iodide, but also observed for ethyl iodide and benzyl bromide, albeit with lower selectivity. In both cases the reactions proceed slower and led to the formation of by-products. In contrast, *tert*-butyl bromide and isopropyl iodide do not undergo any reaction presumably due to steric restrictions. In case of EtI the C–H activation in the Ph_2_PNTol proceeds faster, thus preventing the isolation of 7, which nonetheless could unambiguously be identified by XRD analysis (Fig. 10 and ESI†). The reaction of benzyl bromide required 24 h and 2 equiv. of the bromide to afford complex 8 in 65% yield. We could also confirm the nature of complex 8 through XRD analysis (see Fig. S72 in ESI†), confirming the same constitution of the ligand as in 6, but with a different bonding pattern due to the protonation of the iminophosphine arm and the binding of a second bromide atom at the rhodium center (see the ESI† for a proposed mechanism for the formation of 6).

Overall, the observed reactivities clearly demonstrate the strong bonding of the yldiide ligand to the rhodium center. Despite the high charge concentration at the ylidic carbon atom, the C–Rh linkage remains intact in all transformations. Instead, the high donor capacity leads to a strong activation of the CO ligand (as demonstrated by the low CO stretching frequency), and to transformations directly involving the carbonyl ligand. This effective activation of CO by a *trans*-bound yldiide donor may be used in the future for the development of new catalysts for CO transformations. While the ligand scaffold presented here still suffers from limited stability towards insertion reactions, this deficiency can be considered in the design of new ligands to eventually take advantage of the particular donor strength of yldiide ligands for catalytic applications.

## Conclusions

In conclusion, we reported on the synthesis of a new PCN pincer ligand with an ylide/yldiide moiety as donor site and its coordination ability in different rhodium complexes. Depending on the protonation state of the ylide, the ligand can either function as neutral L_3_ ligand, *e.g.* to form a [(PC(H)N)RhCl] complex, or as anionic L_2_X ligand such as in the corresponding carbonyl complex [(PCN)Rh(CO)]. In its deprotonated form the yldiide interacts with the metal *via* a σ and π donation, thus giving rise to a remarkably high donor strength of the ligand as judged by its CO vibration and comparison to related rhodium pincer complexes. Detailed structural and computational analyses demonstrated that this donor strength is reached despite the only weak π-bonding between the carbon and the rhodium center due to an unfavorable antibonding p_π_–d_y*z*_ interaction. However, this electronic situation results in an increased back-donation from Rh to the CO ligand to decrease the electron density at the metal and prevent electrostatic repulsion with the ylide ligand. Consequently, the corresponding phosphine complexes feature an unusual distortion from the square planar geometry at the rhodium center, in which the phosphine avoids the *trans*-position to the ylide donor to minimize further repulsive interactions.

The high donor ability of the yldiide ligand and the strong donation of electron density into the carbonyl ligand results in a unique reactivity of the Rh carbonyl complex towards alkyl halides. Instead of adding across the Rh–C linkage, small alkyl halides led to the alkylation of the carbonyl ligand which subsequently inserted into the ligand backbone to form a new PCN ligand. These findings demonstrate that the strong CO activation by *trans*-bound yldiide ligands offers new possibilities for developing catalytic transformations of carbon monoxide. Future ligand design should exploit these special donor properties of yldiide donors to enable new catalytic applications.

## Data availability

The characterization of all compounds synthesized, crystallographic analysis of all relevant compounds, and the Cartesian coordinates of all computationally analyzed species are available in the ESI.†

## Author contributions

S. L. has synthesized the ligand and most of the complexes, conducted the XRD analyses and performed the computational studies. P. D. has contributed to the reactivity studies of complex 2, isolated and characterized complexes 6, 7 and 8. V. H. G. has designed the study, supervised the research activities, and prepared the manuscript together with S. L.

## Conflicts of interest

There are no conflicts of interest.

## Supplementary Material

SC-014-D2SC06759E-s001

SC-014-D2SC06759E-s002
