# Supplementary material for: Probing the donor strength of yldiide ligands: synthesis, structure and reactivity of rhodium complexes with a PCylideN pincer ligand
Source: Chem Sci. 2023 Mar 13;14(14):3816–25. doi: 10.1039/d2sc06759e (PMC10074424; doi:10.1039/d2sc06759e)
Supplement: SC-014-D2SC06759E-s001 [file SC-014-D2SC06759E-s001.pdf]

Electronic Supplementary Material (ESI) for Chemical Science.
This journal is © The Royal Society of Chemistry 2023

Supporting Information

Probing the Donor Strength of Yldiide Ligands: Synthesis, Structure and Reactivity of Rhodium Complexes with a $PC_{ylide}N$ Pincer Ligand

Sébastien Lapointe, Prakash Duari, and Viktoria H. Gessner*

Faculty of Chemistry and Biochemistry, Ruhr-University Bochum, 44801 Bochum, Germany
Email: viktoria.gessner@rub.de

Contents

1. Synthetic Procedures 2
1.1. General information 2
1.2. Ligand Synthesis 2
1.3. Synthesis of the rhodium complexes 5
1.4. Reactivity of 2 towards alkyl halides 9
2. NMR spectra of the isolated products 13
3. IR spectra 48
4. Crystal structure analyses 49
4.1. Crystallographic details 49
4.2. Crystal structure analysis of 1-Li 54
4.3. Crystal structure analysis of L2 55
4.4. Crystal structure analysis of complex 1 56
4.5. Crystal structure analysis of complex 2 57
4.6. Crystal structure analysis of complex 4 58
4.7. Crystal structure analysis of complex 5 59
4.8. Crystal structure analysis of complex 6 60
4.9. Crystal structure analysis of complex 7 61
4.10. Crystal structure analysis of complex 8 62
4.11. Crystal structure analysis of complex 9. 63
5. Computational studies 64
5.1. General information and results 64
5.2. Proposed mechanism of the formation of 6 and 7 71
5.3. Cartesian coordinates 73
6. References 92

1. Synthetic Procedures

1.1. General information

All operations were performed using standard Schlenk or glovebox techniques under an Ar atmosphere unless indicated otherwise. Unless otherwise indicated, all solvents and reagents were used as received. Non-deuterated solvents were taken from a solvent purification system (MBRAUN SPS). Deuterated solvents were added to activated 3 Å molecular sieves. PCy_2Cl[1] was prepared according to literature procedures. (acetylacetonato)dicarbonylrhodium(I) and chloro(1,5-cyclooctadiene)rhodium(I) dimer were purchased from Sigma and used as received.

NMR spectra were recorded on Avance-400 spectrometers at 25 °C if not stated otherwise. All values of the chemical shift are in ppm regarding the δ-scale. All spin-spin coupling constants (*J*) are printed in Hertz (Hz). To display multiplicities and signal forms correctly the following abbreviations were used: s = singlet, d = doublet, t = triplet m = multiplet, dd = doublet of doublet, br = broad signal. Signal assignment was supported by, HSQC (^1H / ^{13}C), HMBC (^1H / ^{13}C, ^1H / ^{31}P) correlation experiments.

IR-Spectra were recorded on a Thermo Nicolet iS5 FT-IR in transmission mode with an ATR module at 22°C. Abbreviations are as follows: w – weak, m – medium, s – strong.

Elemental analyses were performed on an Elementar vario MICRO cube elemental analyzer.

HRMS-ESI: An LTQ Orbitrap Velos (Thermo Fisher Scientific, Bremen, Germany) was used for direct infusion via a syringe pump. The heated desolvation capillary was set to 200°C and a spray voltage of 1.8 kV was supplied. In the tune file the LTQ Orbitrap was set to the following parameters (R = 30,000; IT = 500 ms; AGC Target = 1,000,000).

For details about the single-crystal X-ray diffraction analyses, see chapter 4.

1.2. Ligand Synthesis

(^{Ph}BIPM^{Tol}) ligand L1.

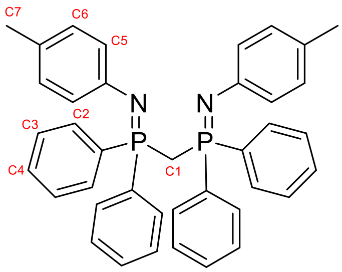

Ligand **L1** was prepared via a slightly modified procedure than previously reported.[2] In a 2-necked 500 mL Schlenk flask was added Bis(diphenylphosphino)methane (dppm) (1.00 eq, 10.0 g, 25.2 mmol) followed by 350 mL of anhydrous toluene. An addition funnel was attached to the RBF and filled with 50mL of anhydrous toluene. The reaction mixture was heated up to 60°C, a condenser was added and topped with a bubbler. Once the temperature reached 60°C, p-Tolyl azide (2.50 eq, 8.39 g, 63.0 mmol) was added to the 50 mL of anhydrous toluene in the addition funnel, and a medium speed addition was started over 15 minutes while heating. Once the addition was finished, the reaction was left to stir at 60°C for 4 hours total. The toluene was evaporated while keeping heating at 40°C until a beige powder was

obtained. An orange impurity was removed by addition of 50 mL of diethyl ether and filtered off using a cannula. The solids are evaporated to obtain ligand **L1** as an off-white powder (14.49g, 96.7%).

^{1}H NMR (400 MHz, THF-d$_8$): δ 2.11 (s, 6H, C7-$\underline{H_3}$), 3.89 (t, J_{HH} = 13.7 Hz, 2H, C1-$\underline{H_2}$), 6.39 (d, J_{HH} = 8.2 Hz, 4H, C5-$\underline{H}$), 6.64 (d, J_{HH} = 7.9 Hz, 4H, C6-$\underline{H}$), 7.22 – 7.28 (m, 8H, C3-$\underline{H}$), 7.35 (td, J = 7.4, 1.4 Hz, 4H, C4-$\underline{H}$), 7.77 (ddd, J = 12.0, 7.4, 1.6 Hz, 8H, C2-$\underline{H}$).

^{31}P{^{1}H} NMR (162 MHz, THF-d$_8$): δ -4.15 (s).

The spectroscopic data are in line with the literature reports.[2]

Lithiated ^{Ph}BIPM^{Tol} ligand, L1-Li.

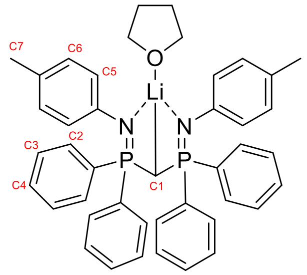

In a 100 mL RBF Schlenk was added **L1** (1 eq, 2 g, 3.36 mmol) in 40 mL of anhydrous THF. Then, the solution was cooled down at -78 degrees and a solution of ^{n}Butyl lithium (1.66 M in hexanes, 1.00 eq, 3.36 mmol, 2.02 mL) was added to the reaction mixture, without any significant color change. The reaction was left to stir at -78°C and slowly over 3h come back to room temperature. The solvent was then evaporated under reduced pressure, and 10 mL of anhydrous diethyl ether was added to facilitate the formation of a solid, then the solvent was evaporated. The lithiated ligand **L1-Li** was obtained as a beige powder (2.01 g, 89%). Colorless crystals suitable for X-ray diffraction analysis were grown through diffusion of pentane in a concentrated THF solution of **L1-Li**.

^{1}H NMR (400 MHz, THF-d$_8$): 1.21 (t, J_{HH} = 2.9 Hz, 1H, C1-$\underline{H}$), 1.75-1.79 (m, 4H, THF), 2.05 (s, 1H, C7-$\underline{H_3}$), 3.60-3.63 (m, 4H, THF), 6.43 (d, J_{HH} = 8.3 Hz, 4H, C5-$\underline{H}$), 6.56 (d, J_{HH} = 8.1 Hz, 4H, C6-$\underline{H}$),7.10 (td, J_{HH} = 7.6, 1.8 Hz, 8H, C3-$\underline{H}$), 7.18 – 7.22 (m, 4H, C4-$\underline{H}$), 7.58o (ddt, J_{HH} = 9.7, 6.8, 1.4 Hz, 8H, C2-$\underline{H}$).

^{31}P{^{1}H} NMR (162 MHz, THF-d$_8$): δ 15.05 (s).

(^{Cy}PC(H)N^{Tol}), ligand L2.

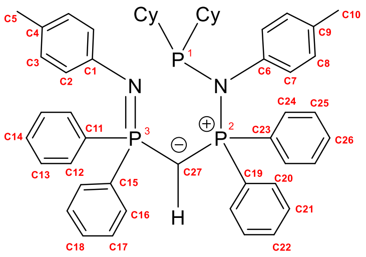

Method 1. In a 100 mL Schlenk flask under an argon atmosphere was added ligand **L1** (1.00 eq., 2.00 g, 3.36 mmol) and anhydrous THF (40 mL) and the flask as cooled to -78°C using an acetone/LN$_2$ bath. Then, a ^{n}Butyl lithium solution (1.66 M in hexanes) (1.00 eq., 2.02 mL, 3.36 mmol) was added and left to stir at room temperature over 3 hours. The solvent was then evaporated under reduced pressure to give a beige powder. 10 mL of diethyl ether was then added, and the solid evaporated under reduced pressure to give the lithiated BIPM ligand **L1-Li**

(2.008 g, 2.991 mmol, 89%). **L1-Li** (1.05 g, 1.56 mmol, 1.00 eq.) was then added to a 100 mL Schlenk flask, and anhydrous THF was added (40 mL). The solution was cooled to -78°C using an ice bath, then dicyclohexylchlorophosphine (380 µL, 1.72 mmol, 1.1 eq.) was added and left to stir at room temperature for 1 hour before the solvent was evaporated. 15 mL of diethyl ether was added to solubilize the ligand but remove the LiCl formed, and the solution was evaporated under reduced pressure. A second portion of diethyl ether is added to remove some of the starting **L1** ligand formed in the reaction as a side-product, and the solvent is removed under reduced pressure to obtain **L2** as a yellow powder (1.02 g, 1.29 mmol, 82.7%).

Method 2. In a 250 mL Schlenk RBF under an argon atmosphere was added the ligand **L1** (1 eq, 4.00 g, 6.73 mmol) and 150 mL of anhydrous THF at 0° using an ice bath, then a ^{n}Butyl lithium solution (1,57 M in hexanes) (1.1 eq, 4.7 mL, 7.4 mmol) was added. In a second 250 mL Schlenk flask was added chlordicyclohexylphosphine (1.2 eq, 1.78 mL, 8.08 mmol) and 50 mL of anhydrous THF, and cooled down. The first solution was transferred into the second Schlenk and was stirred at 0°C for 2h. The solvent was evaporated under reduced pressure, and diethylether was added (100 mL), the LiCl filtered off, and the diethyl ether solution evaporated. Then, the resulting solid was washed 3 times with 50 mL pentanes and evaporated, giving the desired product as a yellow solid (4.11 g, 5.19 mmol, 77 % yield). Yellow crystals suitable for X-ray diffraction studies were grown from diffusion of pentane into a concentrated THF solution **L2**.

^{1}H NMR (400 MHz, THF-d$_8$): δ 0.96-1.21 (m, 12H, P<u>*Cy*</u>), 1.55 (d, 2H, J_{HH} = 9.9 Hz, P<u>*Cy*</u>), 1.62 (d, J_{HH} = 10.3 Hz, 4H, P<u>*Cy*</u>), 1.75-1.78 (m, 2H, P<u>*Cy*</u>), 1.82 (d, J_{HH} = 7.3 Hz, C27-<u>*H*</u>), 2.01 (t, J_{HH} = 10.0 Hz, 2H, P<u>*Cy*</u>), 2.12 (s, 3H, C10-<u>*H$_3$*</u>), 2.24 (s, 3H, C5-<u>*H$_3$*</u>), 6.53 (d, J_{HH} = 8.1 Hz, 2H, C7-<u>*H*</u>), 6.63 (d, J_{HH} = 8.0 Hz, 2H, C2-<u>*H*</u>), 6.66 (dd, J_{HH} = 8.1, 1.0 Hz, 2H, C3-<u>*H*</u>), 6.92 (d, J_{HH} = 8.0 Hz, 2H, C8-<u>*H*</u>), 7.10 (dtd, 10H, J_{HH} = 19.7 Hz, 7.0 Hz, 6.4 Hz, 2.8 Hz, C12-<u>*H*</u> + C13-<u>*H*</u> + C14-<u>*H*</u> + C16-<u>*H*</u> + C17-<u>*H*</u> + C18-<u>*H*</u>), 7.32 (td, J_{HH} = 7.4, 1.5 Hz, 2H, C22-<u>*H*</u>+ C26-<u>*H*</u>), 7.49 (ddd, J_{HH} = 12.7 Hz, 8.2 Hz, 1.4 Hz, 4H, C21-<u>*H*</u> + C25-<u>*H*</u>), 7.75 (ddd, J_{HH} = 11.6 Hz, 8.1 Hz, 1.6 Hz, 4H, C20-<u>*H*</u> + C24-<u>*H*</u>).

^{13}C{^{1}H} (101 MHz, THF-d$_8$): δ 14.82 (^{v}ddd, J_{PC} = 136.2, 109.9, 7.8 Hz, <u>*C27*</u>), 21.06 (<u>*C10*</u>), 21.12 (<u>*C5*</u>), 27.51 (P-<u>*Cy*</u>), 27.88 (^{v}d, J_{PC} = 7.8 Hz, P-<u>*Cy*</u>), 28.67 (^{v}d, J_{PC} = 9.5 Hz, P-<u>*Cy*</u>), 28.79 (^{v}d, J_{PC} = 8.1 Hz, P-<u>*Cy*</u>), 32.65 (^{v}d, J_{PC} = 23.3 Hz, P-<u>*Cy*</u>), 38.32 (^{v}dd, J_{PC} = 24.1 Hz, 3.0 Hz, P-<u>*Cy$_{ipso}$*</u>), 123.15 (<u>*C11*</u>+<u>*C15*</u>), 124.24 (^{v}d, J_{PC} = 20.6 Hz, <u>*C7*</u>), 127.96 (^{v}d, J_{PC} = 8.0 Hz, <u>*C13*</u> + <u>*C17*</u>), 128.08 (^{v}d, J_{PC} = 9.2 Hz, <u>*C12*</u> + <u>*C16*</u>), 129.26 (^{v}d, J_{PC} = 1.8 Hz, <u>*C3*</u>), 129.55 (^{v}d, J_{PC} = 2.7 Hz, <u>*C14*</u> + <u>*C18*</u>), 130.14 (^{v}d, J_{PC} = 1.7 Hz, <u>*C8*</u>), 130.93 (^{v}d, J_{PC} = 106.6 Hz, <u>*C23*</u> + <u>*C19*</u>), 131.61 (^{v}d, J_{PC} = 3.2 Hz, <u>*C2*</u>), 132.02 (^{v}d, J = 2.9 Hz, <u>*C22*</u> + <u>*C26*</u>), 132.99 (^{v}dd, J_{PC} = 8.9 Hz, 1.5 Hz, <u>*C20*</u> + <u>*C24*</u>), 134.89 (^{v}dd, J_{PC} = 10.0 Hz, 3.1 Hz, <u>*C21*</u> + <u>*C25*</u>), 137.30 (^{v}d, J_{PC} = 2.1 Hz, <u>*C4*</u>), 139.65 (^{v}dd, J = 6.0, 1.4 Hz, <u>*C1*</u>), 141.09 (^{v}dd, J_{PC} = 107.8 Hz, 6.7 Hz, <u>*C6*</u>) 152.42 (^{v}d, J = 2.8 Hz, <u>*C9*</u>).

^{31}P{^{1}H} (162 MHz, THF-d$_8$): δ -0.82 (d, J_{PP} = 32.7 Hz, <u>*P3*</u>), 41.64 (dd, J_{PP} = 79.3, 33.1 Hz, <u>*P2*</u>), 63.44 (d, J_{PP} = 79.3 Hz, <u>*P1*</u>).

Elemental analysis: Anal. Calcd. for $C_{51}H_{58}N_2P_3$; Calculated: C, 77.45; H, 7.26; N, 3.54. Found: C, 77.21; H, 7.43; N, 3.60.

1.3. Synthesis of the rhodium complexes

(^{Cy}PC(H)N^{Tol})Rh^{I}Cl, 1.

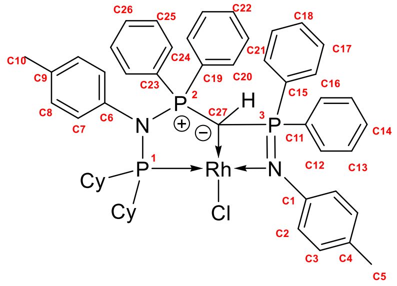

In a 100 mL schlenk was added ligand **L2** (1 eq, 300 mg, 0.379 mmol) and chloro(1,5-cyclooctadiene)rhodium(I) dimer (0.6 eq, 112 mg, 0.227 mmol), followed by anhydrous THF (15 mL). The solution turned brown and was left to stir at room temperature for 24 hours before the solvent was removed under reduced pressure. The solid was washed 6 times with pentanes, and the remaining solid was dried under reduced pressure to afford a brown powder (304 mg, 0.327 mmol, 86%). Brown crystals suitable for X-ray diffraction studies were grown by diffusion of pentane on a concentrated THF solution of **1**. Despite our best efforts and due to the reactive nature of complex **1**, we were not able to obtain satisfactory elemental analyses.

^{1}H NMR (400 MHz, THF-d$_8$): δ 0.62-0.71 (m, 1H, P-*Cy*), 1.03 (q, J_{HH} = 12.7 Hz, 2H, P-*Cy*), 1.26-1.60 (m, 11H, P-*Cy*), 1.81-1.96 (m, 7H, P-*Cy*), 2.20-2.29 (m, 1H, P-*Cy*), 2.39 (t, J_{HH} = 12.2 Hz, P-*Cy*), 3.46-3.54 (m, 1H, C27-*H*), 6.47 (d, J_{HH} = 8.0 Hz, 2H, C8-*H*), 6.83 (d, J_{HH} = 8.1 Hz, 2H, C7-*H*), 6.95 (dt, J_{HH} = 8.0, 4.0 Hz, 4H, C20+C24-*H*), 7.05 (ddd, J_{HH} = 17.9, 9.7, 5.5 Hz, 6H, C21+C22+C25+C26-*H*), 7.12–7.24 (m, 4H, C12+C16-*H*), 7.28 (t, J_{HH} = 7.2 Hz, 1H, C14-*H*), 7.41 (t, J_{HH} = 7.5 Hz, 1H, C18-*H*), 7.57 (dd, J_{HH} = 7.1, 2.5 Hz, 4H, C13+C17-*H*), 8.61–8.68 (m, 2H, C3-*H*), 9.06 (dd, J_{HH} = 14.1, 7.7 Hz, 2H, C2-*H*).

^{13}C{^{1}H} NMR (101 MHz, THF-d$_8$): δ -14.7 (^{v}d, $J_{Rh\text{-}C}$ = 11.8 Hz, *C27*), 20.9 (*C10*), 21.1 (*C5*), 27.10 (P-*Cy*), 27.6 (P-*Cy*), 27.7 (P-*Cy*), 27.8 (P-*Cy*), 27.9 (P-*Cy*), 27.93 (^{V}d, J_{PC} = 7.5 Hz, C-*Py*), 28.6 (P-*Cy*), 28.7 (P-*Cy*). 28.8 (P-*Cy*), 29.9 (*C27*), 30.7 (^{v}d, J_{PC} = 2.4 Hz, C-*Py*), 38.23 (^{v}d, J_{PC} = 16.9 Hz, P-*Cy$_{ipso}$*), 38.6 (^{v}d, J_{PC} = 19.1 Hz, P-*Cy$_{ipso}$*), 123.8 (d, J_{PC} = 10.4 Hz, *C7*), 126.41 (*C1*), 128.07 (^{v}d, J_{PC} = 13.9 Hz, *C12/C16*), 128.3 (^{v}d, J_{PC} = 10.4 Hz, *C21/C25*), 128.6 (^{v}d, J_{PC} = 1.0 Hz, *C8*), 128.9 (^{v}d, J_{PC} = 11.6 Hz, *C20/C24*), 129.8 (^{v}d, J_{PC} = 11.0 Hz, *C13/C17*), 132.1 (d, J_{PC} = 2.2 Hz, *C14*), 132.5 (^{v}d, J_{PC} = 10.6 Hz, *C20/C24*), 132.64 (^{v}d, J_{PC} = 2.7 Hz, *C18*), 132.8 (^{v}d, J_{PC} = 10 Hz, *C3*), 132.7 (*C17*), 132.9 (*C9*), 133.60 (^{v}d, J_{PC} = 8.3 Hz, *C22/C26*), 134.1 (^{v}d, J_{PC} = 9.0 Hz, *C19/C23*), 134.9 (^{v}d, J_{PC} = 9.2 Hz, *C11/C15*), 137.8 (^{v}d, J_{PC} = 13.9 Hz, *C2*), 138.3 (^{v}d, J_{PC} = 2.5 Hz, *C6*), 139.2 (^{v}d, J_{PC} = 3.0 Hz, *C4*), 148.3 (*C1*).

^{31}P{^{1}H} NMR (162 MHz, THF-d$_8$): δ 7.50 (t, $J_{RhP2,P2P1}$ = 33.0 Hz, *P2*), 26.1 (ddd, $^{2}J_{RhP3}$ = 36.2 Hz, $^{2}J_{P3P2}$ = 13.8 Hz, $^{2}J_{P3P1}$ = 10.2 Hz, *P3*), 138.1 (ddd, $^{1}J_{RhP1}$ = 202.4 Hz, $^{2}J_{P1P2}$ = 29.8 Hz, $^{3}J_{P1P3}$ = 9.9 Hz, *P1*).

Elemental Analysis: Anal. Calcd. for $C_{51}H_{57}N_2P_3Rh_1Cl_1$; Calculated: C, 65.92; H, 6.18; N, 3.01; Found: C, 63.82; H, 6.33; N, 2.97.

(^{Cy}PCN^{Tol})Rh^{I}CO, 2.

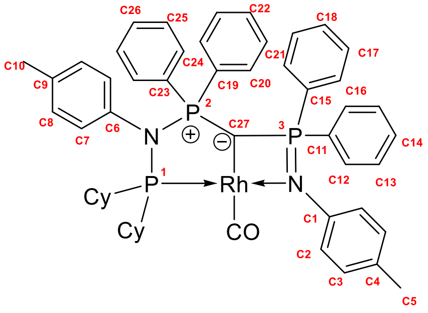

In a Schlenk flask under an argon atmosphere was added ligand **L2** (1.00 eq, 500 mg, 0.632 mmol) and acetylacetonato(dicarbonyl)rhodium(I) (1.00 eq., 163 mg, 0.632 mmol). The flask was taken out of the glovebox, and under an argon atmosphere was added anhydrous THF (25 mL), which produced effervescence through CO evolution and a color change to orange. The solution was left to stir at room temperature for 4 hours before removing the solvent until 2 mL remains, then 20 mL pentanes was added, resulting in a yellow solid crashing out of the solution. Half of the solvent was removed to promote additional solid crashing, and the solvent was filtered off via cannula. The remaining solid was washed 3 times with pentanes to afford **2** as a bright yellow powder (400 mg, 0.434 mmol, 69%). Orange crystals suitable for X-ray diffraction studies were grown by diffusion of pentanes on a concentrated THF solution of **2** at room temperature.

^{1}H NMR (400 MHz, THF-d$_8$): δ 1.08-1.17 (m, 2H, P-*Cy*), 1.20-1.36 (m, 4H, P-*Cy*), 1.41-1.55 (m, 2H, P-*Cy*), 1.65 (d, J_{HH} = 12.5 Hz, 6H, P-*Cy*), 1.78-2.00 (m, 6H, P-*Cy*), 2.05 (s, 3H, C10-*H*), 2.24 (s, 3H, C5-*H*),f 2.26-2.30 (m, 1H, P-*Cy*), 6.49 (d, J_{HH} = 7.9 Hz, C8-*H*), 6.56-6.60 (m, 4H, C2-*H* +C3-*H)*, 6.91 (d, J_{HH} = 8.0 Hz, 2H, C7-*H*), 7.00 (td, J_{HH} = 7.8, 2.8 Hz, 4H, C21-*H* + C25-*H*), 7.09 (td, J_{HH} = 7.7, 2.4 Hz, 4H, C20-*H* +C24-*H*), 7.18-7.28 (m, 8H, C13-*H* + C14-*H* + C17-*H* + C18-*H* + C22-*H* + C26-*H*), 7.49-7.54 (m, 4H, C12-*H* +C16-*H*).

^{13}C{^{1}H} NMR (101 MHz, THF-d$_8$): δ 20.4 (*C10*), 20.8 (*C5*), 27.0 (P-*Cy*), 27.7 (^{v}dd, J_{PC} = 14.4, 13.6 Hz, P-*Cy*), 29.1 (P-*Cy*), 29.4 (d, J_{PC} = 4.1 Hz, P-*Cy*), 40.5 (^{v}d, J_{PC} = 25.0 Hz, P-*Cy$_{ipso}$*), 120.9 (^{v}d, J_{PC} = 14.8 Hz, *C2*), 124.5 (*C11* + *C15*), 127.8 (^{v}d, J_{PC} = 11.9 Hz, *C21*+*C25*), 128.2 (^{v}d, J_{PC} = 11.0 Hz, *C20*+*C24*), 128.6 (*C3*), 130.1 (^{v}d, J_{PC}= 2.9 Hz, *C7*), 130.2 (*C22*+*C26*), 131.3 (^{v}d, J_{PC} = 2.6 Hz, *C14*+*C18*), 131.6 (^{v}d, J_{PC} = 10.3 Hz, *C12*+*C16*), 131.9 (*C8*), 132.4 (^{v}d, J_{PC} = 2.6 Hz, *C19* + *C23*), 133.4 (^{v}d, J_{PC} = 10.6 Hz, *C13*+*C17*), 135.6 (^{v}d, J_{PC} = 76.5 Hz, *C6*), 138.3 (*C4*) 138.5 (^{v}d, J_{PC} = 4.3 Hz, *C1*), 147.5 (^{v}d, J_{PC} = 3.6 Hz, *C9*), 193.0 (^{v}dd, J_{RhC} = 70.7 Hz, J_{PC} = 14.8 Hz, Rh-*C*O). The signal of C27 could not be observed. The coupling constants were mostly assigned via the APT NMR in the supporting information.

^{31}P{^{1}H} NMR (162 MHz, THF-d$_8$): δ 32.68 (ddd, $^{2}J_{RhP}$ = 102.3 Hz, $^{2}J_{P3P1}$ = 20.0, $^{2}J_{P3P2}$ = 16.3 Hz, *P3*), 45.2 (dd, $^{2}J_{RhP}$ = 102.6 Hz, $^{2}J_{P2P1}$ = 68.2 Hz, *P2*), 149.3 (ddd, $^{1}J_{RhP}$ = 177.9 Hz, $^{2}J_{P1P2}$ = 68.3 Hz, $^{2}J_{P1P3}$ = 20.0 Hz, *P1*).

ATR-IR (cm–1): 2926.5 (m), 2847.1 (m), 1910.1 (s, C≡O), 1605.6 (m), 1501.02 (s), 1434.1 (m), 1312.3 (m), 1264.3 (w), 1193.0 (s), 1173.6 (s), 1103.8 (s), 1020.10 (w), 986.0 (m), 959.5 (m), 927.4 (s), 908.9 (s), 846.4 (w), 814.4 (m), 761.9 (m), 738.4 (s), 713.9 (s), 689.5 (s), 662.7 (m), 642.0 (w), 620.9 (w), 613.1 (w), 585.9 (s), 573.4 (s), 553.4 (s), 521.0 (s), 495.4 (s), 476.9 (s), 450.5 (m) 417.5 (w).

ESI-HRMS (*m/z*) for [$C_{52}H_{56}N_2O_1P_3Rh$ + H]$^+$; Calculated: 921.2739. Obtained for = 921.2727.

Elemental Analysis: Anal. Calcd. For $C_{52}H_{56}N_2O_1P_3Rh_1$:C, 67.82; H, 6.13; N, 3.04. Found: C, 67.56; H, 6.10; N, 3.08.

(^{Cy}PCN^{Tol})Rh^{I}PPh$_3$, 3.

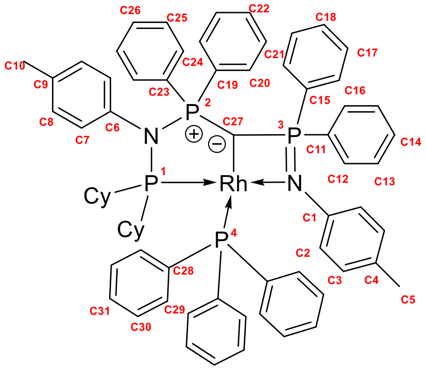

In a 100 mL Schlenk was added complex **1** (1.00 eq, 300 mg, 0.323 mmol), NaHMDS (1.10 eq, 65.2 mg, 0.355 mmol), and PPh_3 (1.00 eq, 84.7 mg, 0.323 mmol), before adding 20 mL of anhydrous THF. The solution turned dark red, and was left to stir at room temperature for 1 hour. The solvent was evaporated under reduced pressure, then pentane was added, the solid was scratched from the walls, and further stirred in pentanes. The mixture was filtered using a cannula filter, and the solid was further washed three times with pentanes. Complex **3** was obtained as a brown solid that is sparsely soluble in organic solvents (256 mg, 0.222 mmol, 68.6%). Despite our best efforts, we were not able to obtain satisfactory elemental analysis due to the low solubility of **3** in all common organic solvents.

^{1}H NMR (400 MHz, C_6D_6): δ 0.92-1.06 (m, 4H, P-Cy), 1.18-1.32 (m, 7H, P-Cy), 1.61 (d, J_{HH} = 13.9 Hz, 4H, P-Cy), 1.76 (d, J_{HH} = 15.5 Hz, P-Cy), 1.85 (s, 3H, C10-H), 1.88 (s, 3H, C5-H), 2.01-2.13 (m, 3H, P-Cy), 6.20-6.26 (m, 4H, C2 + C3-H), 6.57 (d, J_{HH} = 8.0 Hz, C8-H), 6.79 (d, J_{HH} = 8.1 Hz, C7-H), 6.93-7.06 (m, 15H, C29 + C30 + C31-H), 7.09-7.15 (m, 6H, C13+C14+C17+C18-H), 7.84 (t, J_{HH} = 9.8 Hz, 4H, C12+C16-H), 7.92 (t, J_{HH} = 9.1 Hz, 4H, C20+C24-H), 8.33 (t, J_{HH} = 8.4 Hz, 6H, C21+C22+C25+C26).

^{13}C{^{1}H} NMR (101 MHz, C_6D_6): δ 14.32 (d, J_{PC} = 7.2 Hz, C27), 20.73 (C10), 20.88 (C5), 26.83 (Cy), 26.96 (Cy), 27.11 (Cy), 27.24 (Cy), 27.80 (d, J_{PC} = 9.8 Hz, Cy), 28.51 (d, J_{PC} = 5.3 Hz, Cy), 38.31 (d, J_{PC} = 19.3 Hz, Cy), 126.64 (d, J_{PC} = 2.7 Hz, C1), 127.14 (C14+C18), 127.24 (C13+C17), 127.56 (C31), 127.69 (d, J_{PC} = 10.2 Hz, C2), 127.84 (C3), 128.42 (d, J_{PC} = 13.1 Hz, C29), 129.56 (C8), 129.94 (C30), 131.53 (d, J_{PC} = 9.3 Hz, C20+C24), 132.30 (d, J_{PC} = 1.5 Hz, C7), 132.41 (C4), 132.50 (C9), 133.46 (d, J_{PC} = 9.3 Hz, C12+C16), 136.07 (d, J_{PC} = 13.1 Hz, C21+C22+C25+C26), 136.63 (d, J_{PC} = 2.0 Hz, C28), 140.15 (C11+C15), 140.45 (C19+C23), 147.67 (C6).

^{31}P{^{1}H} NMR (162 MHz, C_6D_6): δ 33.31 (ddt, $^2J_{RhP3}$ = 98.8 Hz, J_{P3P2} = 17.9 Hz, J_{P3P4} = 14.4 Hz, P3), 36.46 (ddd, $^2J_{RhP2}$ = 99.2 Hz, $^2J_{P2P1}$ = 64.2 Hz, $^3J_{P2P4}$ = 10.4 Hz, P2), 44.05 (dddd, $^1J_{RhP4}$ = 169.9 Hz, $^2J_{P4P1}$ = 29.0 Hz, $^3J_{P4P3}$ = 14.5 Hz, $^3J_{P4P2}$ = 10.2 Hz, P4), 148.76 (dddd, $^1J_{RhP1}$ = 202.5 Hz, $^2J_{P1P2}$ = 63.9 Hz, $^2J_{P1P4}$ = 29.2 Hz, $^3J_{P1P3}$ = 14.3 Hz, P1).

Note: To probe whether all solid material of the reaction is indeed complex **3**, we reacted the highly insoluble material with ethereal HCl. Indeed, the reaction mixture reacted with HCl to form a new species which we identified as the cationic, protonated version of complex **3** with Cl as counter anion. Accordingly, a new set of signals was observed by ^{31}P{^{1}H} NMR spectroscopy.

The spectrum of the reaction mixture is shown in Figure S61. The high selectivity (> 95% according to 31P NMR) of this transformation clearly confirms the uniformity of the obtained solid.

$^{31}P\{^1H\}$ NMR (162 MHz, THF-d_8): δ 26.8 (dt, *J* = 27.1, 12.0 Hz), 38.7 (d, *J* = 33.9 Hz), 52.5 (td, *J* = 35.7, 15.2 Hz), 125.6 (dd, *J* = 134.5, 30.1 Hz).

(^{Cy}PCN^{Tol})Rh^{I}PMe$_3$, 4

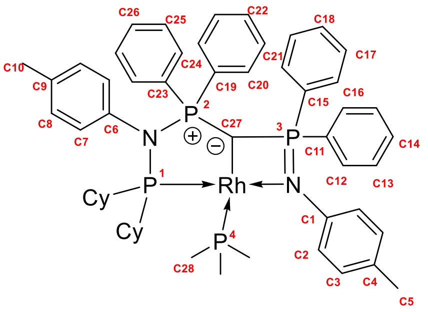

In a 100 mL Schlenk was added complex **1** (1.0 eq, 50 mg, 0.054 mmoles) and sodium hexamethyldisilazane (1.10 eq. 10.9 mg, 0.0590 mmoles). Then, 10 mL of anhydrous THF was added to the Schlenk, and quickly cooled down to -78°C. To the cold solution was added trimethylphosphine (10.0 eq. 46.9 µL, 0.538 mmoles). A color change from deep red to deep brown was observed immediately, and the solution was left to stir at low temperature for 30 minutes, before the solvent was removed under reduced pressure to afford a red residue. The residue was dissolved in diethyl ether and filtered through cannula. The solution was evaporated quickly as it decomposes under reduced pressure, giving a dark brown solid (34.0 mg, 65%, 0.0351 mmoles). Deep brown crystals suitable for X-ray diffraction analysis were grown from hexane diffusion into a concentrated deuterated benzene solution of **4** at room temperature under an atmosphere of argon.

Due to the highly sensitive nature of the complex under reduced pressure, and the decomposition of the complexes upon attempts to further purity through washings or even evaporation of crystals of the complex, we report the NMRs containing decomposition products and solvents, where the product is identified.

^1H NMR (400 MHz, C_6D_6): 0.97-1.17 (m, 9H, P-*Cy*), 1.34 (d, J_{PH} = 6.7 Hz, P-C*H$_3$*), 1.65-1.81 (m, 7H, P-*Cy*), 1.94-2.02 (m, 5H, P-*Cy*), 2.21 (s, 1H, P-*Cy*), 6.61 (d, J_{HH} = 8.0 Hz, C2-*H*), 6.72-6.78 (m, 2H, C14+C18-*H*), 6.84 (d, J_{HH} = 8.8 Hz, C3-*H*), 6.88 (dd, J_{HH} = 7.9 Hz, J_{PH} = 2.8 Hz, C12+C16-*H*), 6.92-7.00 (m, 10H, C13+C17+C21+C22+C25+C26-*H*), 7.73 (d, J_{HH} = 8.2 Hz, C7-*H*), 7.75 (d, J_{HH} = 8.2 Hz, C8-*H*), 7.92 (t, J_{HH} = 8.8 Hz, C20+C24-*H*).

$^{13}C\{^1H\}$ NMR (400 MHz, C_6D_6): 20.93 (*C5*+*C10*), 21.63 (d, J_{PC} = 20.1 Hz, *C28*), 27.21 (P-*Cy*), 27.82 (P-*Cy*), 27.94 (P-*Cy*), 28.02 (P-*Cy*), 28.11 (P-*Cy*), 29.52 (d, J_{PC} = 6.1 Hz, P-*Cy*), 127.16 (d, J_{PC} = 11.4 Hz, *C12*+*C16*), 127.63 (d, J_{PC} = 10.7 Hz, C*13*+C*17*) 128.35 (*C22*+*C26*), 128.90 (*C3*), 129.46 (*C2*), 129.78 (*C21*+*C25*), 131.42 (J_{PC} = 10.0 Hz, *C20*+*C24*), 132.14 (*C14*+*C18*), 133.15-133.35 (m, *C7*+*C8*), 136.54 (*C11*+*C15*+*C19*+*C23*), 138.92 (*C4*), 139.66 (*C1*), 140.62 (*C9*), 151.36 (*C6*).

$^{31}P\{^1H\}$ NMR (162 MHz, C_6D_6): δ -13.42 (dddd, $^1J_{RhP4}$ = 163.3 Hz, $^2J_{P4P1}$ = 31.5 Hz, $^3J_{P4P3}$ = 15.6 Hz, $^3J_{P4P2}$ = 11.1 Hz, *P4*), 28.77 (dtd, $^2J_{RhP2}$ = 101.0 Hz, $^3J_{P2P4}$ 16.6 Hz, J_{P3P1} = 11.2 Hz, *P3*), 35.76

(ddd, $^{2}J_{RhP2}$ = 100.8 Hz, $^{2}J_{P2P1}$ = 66.1 Hz, J_{P2P4} = 11.2 Hz, *P2*), 149.97 (dddd, $^{1}J_{RhP1}$ = 203.8 Hz, $^{2}J_{P1P2}$ = 65.6 Hz, $^{2}J_{P1P4}$ = 31.6, $^{3}J_{P1P3}$ = 11.2 Hz, *P1*).

1.4. Reactivity of 2 towards alkyl halides

[(^{Cy}PC(H)N^{Tol})Rh^{I}CO][I], 5.

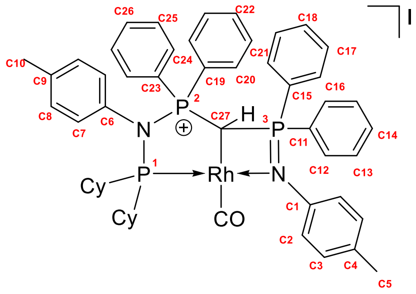

In a 100 mL Schlenk was added complex **2** (1.00 eq., 192 mg, 0.208 mmoles) and anhydrous chloroform, followed by isopropyl iodide (10.0 eq., 357 mg, 210 □L, 2.08 mmoles) and left to stir under argon for 72 hours at room temperature. A slight color change from light orange to darker orange was observed a few minutes after addition of isopropyl iodide. The solvent was then evaporated under reduced pressure and washed multiple times with pentanes to afford a brown powder of complex **5** (165 mg, 0.151 mmoles, 72.7%).

^{1}H NMR (400 MHz, CDCl$_3$): δ 0.45 (q, J_{HH} = 10.9, 10.2 Hz, 1H, P-*Cy*), 1.01 (q, J_{HH} = 13.3, 12.9 Hz, 2H, P-*Cy*), 1.22–1.54 (m, 6H, P-*Cy*), 1.63 (d, J_{HH} = 8.7 Hz, 6H, P-*Cy*), 1.87 (d, J_{HH} = 7.2 Hz, 2H, P-*Cy*), 1.94 (d, J_{HH} = 10.7 H, 4H, P-*Cy*), 2.05 (d, J_{HH} = 13.1 H, 1H, C27-*H*), 2.14 (s, 3H, C10-*H*), 2.28 (s, 3H, C5-*H*), 2.48-2.58 (br s, 1H, P-*Cy*), 6.06 (d, J_{HH} = 7.9 Hz, 1H, C20-*H*), 6.55 (d, J_{HH} = 7.6 Hz, 2H, C3-*H*), 6.58 (s (overlap), 1H, C20-*H*), 6.78 (d, J_{HH} = 7.0 Hz, 2H, C2-*H*), 6.85-7.06 (m, 8H, C12+C13+C16+C17-*H*), 7.28 (d (overlap with CDCl$_3$), J_{HH} = 8.4 Hz, 2H, C24-*H*), 7.33 (t, J_{HH} = 6.5 Hz, 1H, C18-*H*), 7.37-7.46 (m, 3H, C21+C22-*H*), 7.55 (t, J_{HH} = 6.6 Hz, 1H, C14-*H*), 7.67 (d, J_{HH} = 5.6 Hz, 2H, C7-*H*), 7.73 (d, J_{HH} = 5.5 Hz, 2fH, C8-*H*), 7.96-8.10 (m, 3H, C25+C26-*H*).

^{13}C{^{1}H} NMR (101 MHz, CDCl$_3$): δ 25.85 (P-*Cy*), 26.13 (d, J_{PC} = 14.2 Hz, P-*Cy*), 26.14 (P-*Cy*), 26.60 (d, J_{PC} = 12.2 Hz, P-*Cy*), 27.28 (d, J_{PC} = 2.4 Hz, P-*Cy*), 27.43 (d, J_{PC} = 1.9 Hz, P-*Cy*), 27.55 (d, J_{PC} = 5.2 Hz, P-*Cy*), 27.81 (d, J_{PC} = 6.9 Hz, P-*Cy*), 29.09 (P-*Cy*), 29.93 (P-*Cy*), 37.55 (d, J_{RhC} = 24.3 Hz, *C27*), 121.04 (d, J_{PC} = 14.2 Hz, *C2*), 121.97 (*C11*/*16*), 124.34 (*C19*/*C23*), 125.16 (*C9*), 129.19 (*C24*), 129.24 (*C20*), 129.34 (d, J_{PC} = 3.7 Hz, *C3*), 129.46 (d, J_{PC} = 4.0 Hz, *C13*), 129.68 (*C22*), 129.80 (*C20'*), 129.86 (*C21*), 130.18 (d, J_{PC} = 11.6 Hz, *C7*), 130.74 (*C17*), 131.00 (d, J_{PC} = 10.3 Hz, *C26*), 131.35 (d, J = 11.0 Hz, *C12*), 131.76, 132.57 (d, J_{PC} = 9.6 Hz, *C16*), 133.30 (d, J_{PC} = 3.1 Hz, *C18*), 133.89 (d, J_{pc} = 3.0 Hz, *C8*), 134.45 (d, J_{PC} = 3.3 Hz, *C14*), 134.47 (d, J_{PC} = 12.2 Hz, *C25*), 134.75 (dt, J_{PC} = 4.4, 2.2 Hz, *C6*), 135.37 (d, J_{PC} = 2.7 Hz, *C8'*), 140.52 (d, J = 1.8 Hz, *C1*), 144.32 (*C4*), 189.53 (Rh-*C*O).

^{31}P{^{1}H} NMR (162 MHz, CDCl$_3$): δ 37.05 (dddd, $^{2}J_{RhP3}$ = 26.5 Hz, $^{3}J_{P3P1}$ = 19.5 Hz, $^{2}J_{P3P2}$ = 13.3 Hz, $^{1}J_{P3C1}$ = 6.7 Hz, *P3*), 50.76 (ddd, $^{2}J_{P2P1}$ = 38.5 Hz, $^{2}J_{RhP2}$ = 26.8 Hz, $^{1}J_{P2C1}$ = 4.3 Hz, *P2*), 144.79 (ddd, $^{1}J_{RhP1}$ = 155.7 Hz, $^{2}J_{P1P2}$ = 38.6 Hz, $^{3}J_{P1P3}$ = 19.1 Hz, *P1*).

ATR-IR (cm^{-1}): 2924.53 (m), 2849.75 (m), 1968.05 (m; CO), 1607.19 (w), 1586.54 (w), 1502.92 (m), 1479.24 (w), 1435.88 (m), 1288.26 (w), 1261.98 (m), 1208.47 (w), 1187.70 (w), 1150.31 (w),

1102.16 (m), 1061.21 (m), 1017.44 (m), 960.40 (m), 933.62 (m), 920.62 (m), 892.44 (w), 850.90 (w), 818.21 (m), 738.36 (m), 713.82 (m), 688.34 (m), 655.30 (m), 626.35 (w), 602.83 (w), 536.40 (m), 518.03 (m), 471.84 (m), 446.00 (w), 418.77 (w), 404.41 (w).

Elemental analysis: Anal. Calcd. For $C_{52}H_{57}I_1N_2OP_3Rh_1$: C, 59.55; H: 5.48; N, 2.67. Found: C, 59.34; H, 5.66; N, 2.37.

(^{Cy}POC(Me)CP^{Ph}N^{Tol})Rh^{III}I(PPh$_2$NTol), 6.

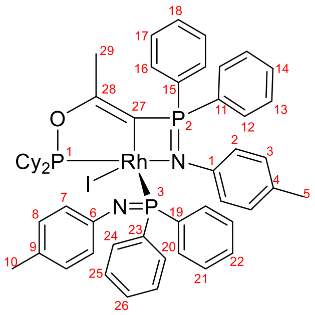

In a J. Young NMR tube was added complex **2** (1.00 eq, 32.0 mg, 0.0347 mmoles), and 1 mL of anhydrous THF was added. The solution was cooled down to -30 °C. Then, methyl iodide (1.50 eq. 3.24 µL, 0.0521 mmoles) was added at -30°C. A color change from yellow to deep purple was observed. The solution was further stirred for 90 minutes. Black crystals suitable for X-ray diffraction studies were obtained by vapor diffusion of pentanes into the THF solution (27 mg, 73%, 0.025 mmol).

^{1}H NMR (400 MHz, THF-d$_8$): δ 1.03-1.54 (m, overlap, 17H + 3H, P-Cy + C29-*H*), 1.64-1.71 (m, 3H, P-Cy), 2.05 (d, $^4J_{HH}$ = 1.8 Hz, 3H, C10-*H*), 2.16 (s, 3H, C5-*H*), 2.38-2.48 (m, 1H, P-Cy), 2.68 (t, $^2J_{HH}$ = 11.6 Hz, 1H, P-Cy), 6.24 (dd, $^3J_{HH}$ = 8.2 Hz, 2H, C7-*H*), 6.49 (d, $^3J_{HH}$ = 7.5 Hz, 2H, C3-*H*) 6.74 (dd, $^3J_{HH}$ = 8.2 Hz, 2H, C8-*H*), 6.95 (td, $^3J_{HH}$ = 7.8 Hz, $^4J_{HP}$ = 3.0 Hz, 2H, C25-*H*), 7.13 (td, $^3J_{HH}$ = 7.4 Hz, $^4J_{HP}$ = 3.5 Hz, 2H, C21-*H*), 7.16-7.25 (m, 4H, C14+C18+C22+C26-*H*), 7.31 (d, $^3J_{HH}$ = 8.4 Hz, 2H, C3-*H*), 7.33-7.37 (m, 2H (overlaps with C2-*H*), C13-*H*), 7.42 (td, $^3J_{HH}$ = 7.6 Hz, $^4J_{HP}$ = 2.2 Hz, 2H, C17-*H*), 7.49-7.60 (m, 2H, C12-*H*), 7.72 (ddd, $^3J_{HH}$ = 11.4 Hz, $^3J_{HP}$ = 8.2 Hz, $^4J_{HH}$ = 1.0 Hz, 2H, C16-*H*), 7.82 (dd, $^3J_{HH}$ = 11.6 Hz, $^3J_{HP}$ = 7.4 Hz, 2H, C24-*H*), 8.30 (dd, $^3J_{HH}$ = 12.2 Hz, $^3J_{HP}$ = 7.4 Hz, 2H, C20-*H*).

^{13}C{^{1}H} NMR (101 MHz, THF-d$_8$): δ 18.28 (^{v}dd, $^3J_{RhC}$ = 10.3 Hz, $^3J_{PC}$ = 2.7 Hz, *C29*), 20.90 (*C5*), 21.29 (*C10*), 23.38 (P-Cy), 23.69 (P-Cy), 26.55 (P-Cy), 26.55 (P-Cy), 27.63 (d, J_{PC} = 9.1 Hz, P-Cy), 27.79 (d, J_{PC} = 8.9 Hz, P-Cy), 28.41 (d, J_{PC} = 9.0 Hz, P-Cy), 29.86 (d, J_{PC} = 5.8 Hz, P-Cy), 32.71 (P-Cy), 33.53 (P-Cy), 35.44 (d, J_{RhC} = 40.2 Hz, *C27*), 39.56 (d, J_{PC} = 24.9 Hz, P-Cy), 44.77 (d, J_{PC} = 22.7 Hz, P-Cy), 124.36 (d, J_{PC} = 14.6 Hz, *C3*), 125.52 (d, J_{PC} = 18.8 Hz, *C7*), 127.09 (*C4*), 127.13 (*C9*),127.67 (d, J_{PC} = 12.0 Hz, *C21*), 128.00 (d, J_{PC} = 11.5 Hz, *C25*), 129.18 (*C8*), 129.22 (*C4*), 129.74 (*C13*), 129.85 (*C17*), 130.10 (*C14*), 130.13 (*C18*), 130.55 (*C11*), 130.93 (*C15*), 131.29 (*C19*), 131.65 (*C23*), 132.96 (d, J_{PC} = 2.7 Hz, *C22*), 133.13 (d, J_{PC} = 10.1 Hz, *C12*), 133.39 (d, J_{PC} = 10.1 Hz, *C16*), 133.41 (d, J_{PC} = 2.9 Hz, *C26*), 133.81 (d, J_{PC} = 11.0 Hz, *C24*), 135.63 (d, J_{PC} = 10.5 Hz, *C20*), 146.60 (*C1*), 149.98 (*C6*), 163.47 (*C28*).

^{31}P{^{1}H} NMR (162 MHz, THF-d$_8$): δ 33.71 (dd, $^1J_{RhP3}$ = 111.2 Hz, $^2J_{P1P3}$ = 43.6 Hz, *P3*), 39.13 (d, $^2J_{RhP2}$ = 9.6 Hz, *P2*), 185.46 (dd, $^1J_{RhP1}$ = 165.3 Hz, $^2J_{P1P3}$ = 42.9 Hz, *P1*).

Elemental analysis: Anal. Calcd. For $C_{53}H_{59}I_1N_2O_1P_3Rh_1$: C, 59.90; H, 5.60; N, 2.64. Found: C, 60.15; H, 5.39; N, 2.52.

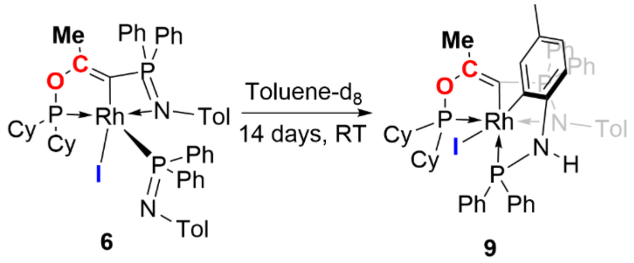

Note: Complex **6** was found to be unstable in THF solution. After 14 d, complete conversion to a decomposition product was observed, which could be identified as the C-H activation product, complex **9** by XRD analysis (Figure S70). Figure S57 shows the ^{31}P NMR spectra of the solution of **6** in THF after different reaction times. **$^{31}P\{^1H\}$ NMR (162 MHz, THF-d$_8$):** δ 35.39 (dd, J_{RhP} = 8.1 Hz, J_{PP} = 4.8 Hz, <u>*P2*</u>), 64.91 (dd, J_{RhP} = 106.5 Hz, J_{PP} = 7.1 Hz, <u>*P3*</u>), 179.43 (dt, J_{RhP} = 149.3, J_{PP} = 7.1 Hz, <u>*P1*</u>).

(^{Cy}POC(Et)CP^{Ph}N^{Tol})Rh^{III}I(PPh$_2$NTol), 7.

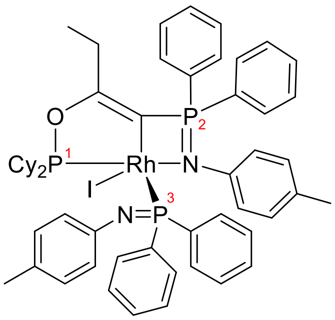

In a J. Young NMR tube was added complex **2** (1.00 eq, 30.0 mg, 0.0325 mmoles), and 1 mL of anhydrous toluene-d8 was added. The solution was cooled down to -30 °C. Then, ethyl iodide (1.50 eq. 3.9 µL, 0.0488 mmoles) was added at -30 °C. A color change from yellow to deep purple was observed. This reaction is slower than the MeI reaction. After 16 h the formation of complex **7** was noticed along with the little bit amount of starting material and the C-H bond activated product (see Figure **S57**). Black crystals of complex **7** suitable for X-ray diffraction studies were obtained by vapor diffusion of pentane into the toluene solution. Complex **7** was not isolated due to formation of C-H bond activated product.

$^{31}P\{^1H\}$ NMR (162 MHz, toluene-d8): δ 36.37 (dd, $^1J_{RhP3}$ = 110.0 Hz, $^2J_{P1P3}$ = 39.5 Hz, <u>*P3*</u>), 37.87 (d, $^2J_{RhP2}$ = 9.7 Hz, <u>*P2*</u>), 184.45 (dd, $^1J_{RhP1}$ = 165.0, $^2J_{P1P3}$ = 39.4 Hz, <u>*P1*</u>).

Note: Similar to complex **6**, complex **7** converts to the C-H activation product analogous to complex **9** (see above). Complete conversion was observed after 10d reaction time (See Figure S57)**. $^{31}P\{^1H\}$ NMR** (162 MHz, toluene-d8)**:** δ 36.43 (dd, J_{RhP} = 8.5 Hz, J_{PP} = 4.7 Hz, <u>*P2*</u>), 64.42 (dd, J_{RhP} = 105.6, J_{PP} = 5.9 Hz, <u>*P3*</u>), 179.67 (dt, J_{RhP} = 147.1, J_{PP} = 5.9 Hz, <u>*P1*</u>).

(^{Cy}POC(CH_2Ph)CP^{Ph}N^{Tol})Rh^{III}Br_2(PPh_2NHTol), 8.

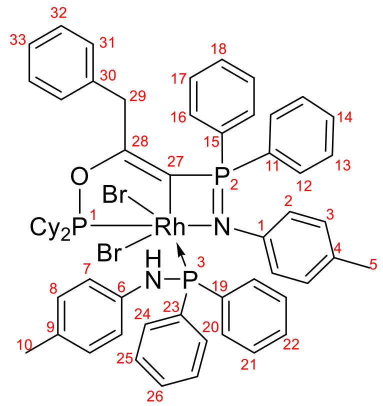

In a J. Young NMR tube was added complex **2** (1.00 eq, 30.0 mg, 0.0325 mmoles), and 1 mL of anhydrous THF was added. The solution was cooled down to -30°C. Then, benzyl bromide (2 eq., 7.77 µL, 0.065 mmoles) was added at -30°C. A color change from yellow to deep purple was observed. The solution was stirred for 24 hours. Almost full conversion was observed, along with some impurities. Black crystals suitable for x-ray diffraction studies were obtained by vapor diffusion of pentanes into the THF solution (24.7 mg, 65%, 0.0325 mmol).

^{1}H NMR (400 MHz, THF-d$_8$): δ 0.40-0.67 (m, 2H, P-Cy), 0.72-1.20 (m, 8H, P-Cy), 1.24-1.40 (m, 6H, P-Cy), 1.59 (t, J_{HH} = 13.0 Hz, 2H, P-Cy), 1.76-1.83 (m, 1H, P-Cy), 2.13 (d, J_{HH} = 1.5 Hz, 3H, C10-*H*), 2.15 (s, 3H, C5-*H*), 2.22 (d, J_{HP} = 14.7 Hz, 2H, C29-*H*), 2.28-2.39 (m, 1H, P-Cy), 3.17 (d, J_{PH} = 14.7 Hz, 1H, N-*H*), 3.27-3.38 (m, 1H, P-Cy), 6.14 (dd, J_{HH} = 8.0 Hz, J_{HP} = 1.5 Hz, 2H, C7-*H*), 6.26 (d, J_{HH} = 6.8 Hz, 2H, C31-*H*), 6.63 (d, J_{HH} = 7.6 Hz, 2H, C8-*H*), 6.73 (d, J_{HH} = 8.3 Hz, 2H, C3-*H*), 6.92-6.98 (m, 2H, C24-*H*), 6.97-7.06 (m, 3H, C32+C33-*H*), 7.14-7.21 (m, 4H, C14+C18+C22+C26-*H*), 7.30 (d, J_{HH} = 8.3 Hz, 2H, C2-*H*), 7.33-7.44 (m, 4H, C13+C17-*H*), 7.52 (td, J_{HH} = 7.9 Hz, J_{HP} = 2.6 Hz, 2H, C21-*H*), 7.59 (td, J_{HH} = 7.0 Hz, J_{HP} = 1.5 Hz, 1H, C25-*H*), 7.65 (td, J_{HH} = 7.3 Hz, J_{HP} = 1.1 Hz, 1H, C25-*H*), 7.89 (ddd, J_{HH} = 18.6 Hz, 11.5 Hz, 4H, C12+C16-*H*), 8.46 (td, J_{HH} = 12.2 Hz, 6.9 Hz, J_{HP} = 3.0 Hz, 2H, C20-*H*).

^{13}C{^{1}H} NMR (101 MHz, THF-d$_8$): δ 20.91 (*C5*), 21.25 (*C10*), 26.23 (P-Cy), 26.57 (P-Cy), 26.62 (d, J_{PC} = 4.4 Hz, P-Cy), 27.25 (P-Cy), 27.40 (P-Cy), 27.55 (d, J_{PC} = 9.4 Hz, P-Cy), 27.76 (P-Cy), 27.90 (P-Cy), 28.35 (d, J_{PC} = 8.8 Hz, P-Cy), 28.62 (d, J_{PC} = 4.9 Hz, P-Cy), 31.83 (P-Cy), 33.0 (*C27*), 40.01 (d, J_{RhC} = 8.3 Hz, *C29*), 44.58 (P-Cy), 123.66 (d, J_{PC} = 16.4 Hz, *C2*), 125.98 (d, J_{PC} = 17.3 Hz, *C7*), 126.89 (*C4*), 126.92 (*C9*), 127.28 (*C33*), 127.74 (d, J_{PC} = 12.2 Hz, *C22*+*C26*), 128.15 (d, J_{PC} = 11.5 Hz, *C24*), 128.39 (*C30*), 128.95 (*C32*), 129.26 (*C8*+*C3*), 129.73 (*C13*), 129.85 (*C14*+*C18*), 130.22 (d, J_{PC} = 11.0 Hz, *C21*), 130.57 (*C31*), 131.36 (*C19*+*C23*), 132.05 (*C11*+*C15*), 132.85 (d, J_{PC} = 2.8 Hz, *C25*), 133.25 (*C12*), 133.40 (d, J_{PC} = 7.6 Hz, *C16*), 133.59 (d, J_{PC} = 10.0 Hz, *C17*), 133.66 (*C25*), 135.46 (d, J_{PC} = 10.3 Hz, *C20*), 137.47 (*C1*), 146.99 (d, J_{PC} = 2.2 Hz, *C6*) 164.30 (*C28*).

^{31}P{^{1}H} NMR (162 MHz, THF-d$_8$): δ 30.98 (dd, $^{1}J_{RhP2}$ = 111.6 Hz, $^{2}J_{P1P2}$ = 44.8 Hz, *P2*), 37.97 (d, $^{2}J_{RhP3}$ = 9.5 Hz, *P3*), 182.28 (dd, $^{1}J_{RhP1}$ = 165.3 Hz, $^{2}J_{P1P2}$ = 43.8 Hz, *P1*).

Elemental analysis: Anal. Calcd. For $C_{59}H_{64}Br_2N_2O_1P_3Rh_1$: C, 60.42; H, 5.50; N, 2.39. Found: C, 60.81; H, 5.7; N, 2.38.

2. NMR spectra of the isolated products

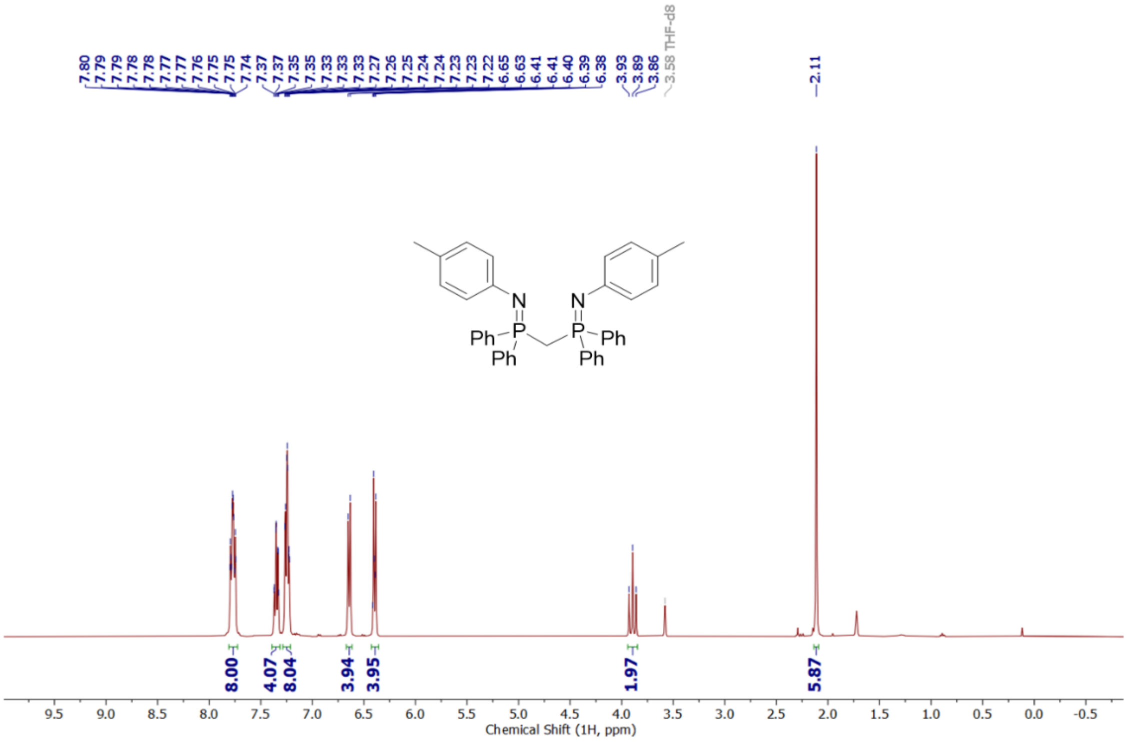

Figure S1. ^{1}H NMR spectrum of ligand **L1** (400 MHz, THF-d_8).

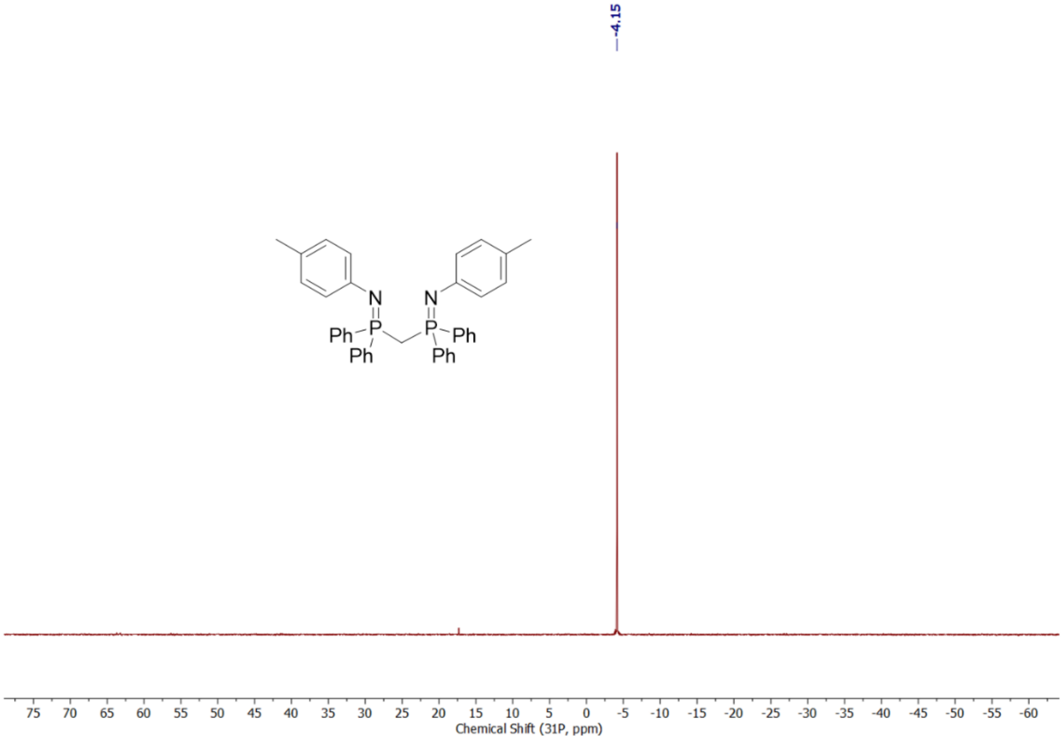

Figure S2. $^{31}P\{^{1}H\}$ NMR spectrum of ligand **L1** (162 MHz, THF-d_8).

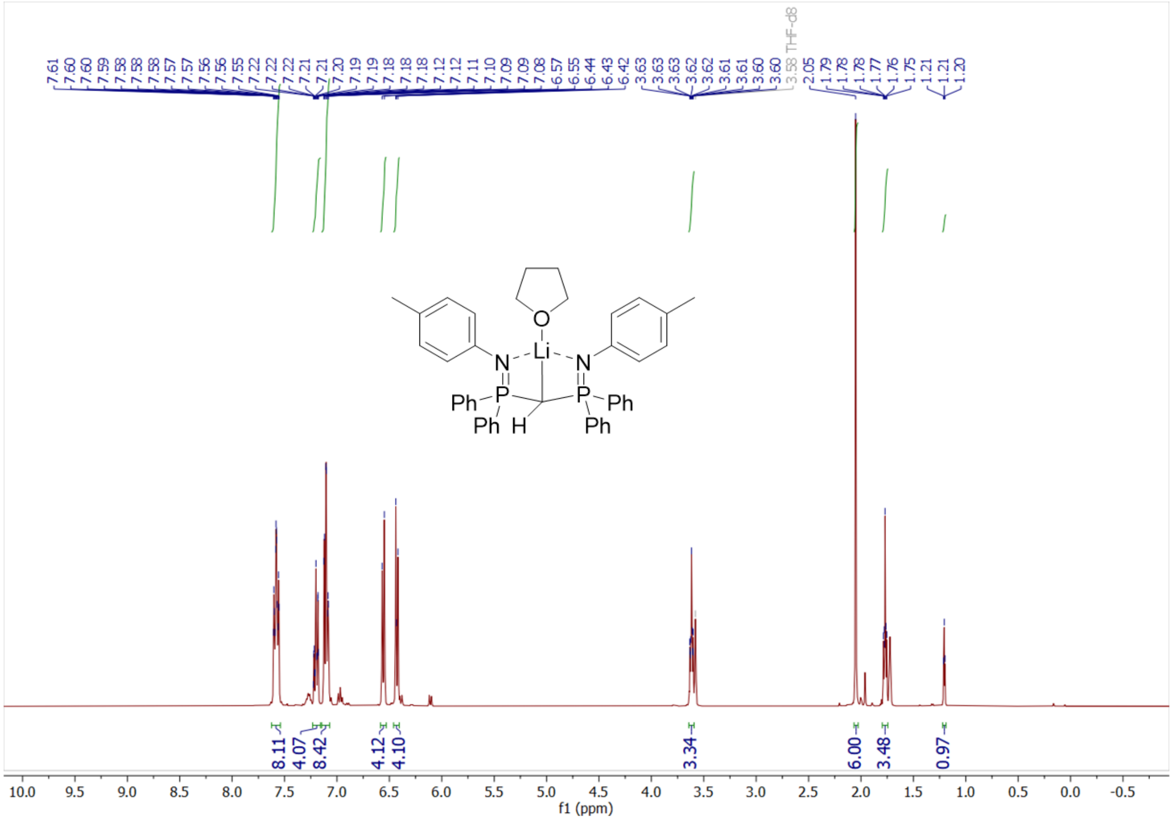

Figure S3. ^{1}H NMR spectrum of ligand **L1-Li** (400 MHz, THF-d_8).

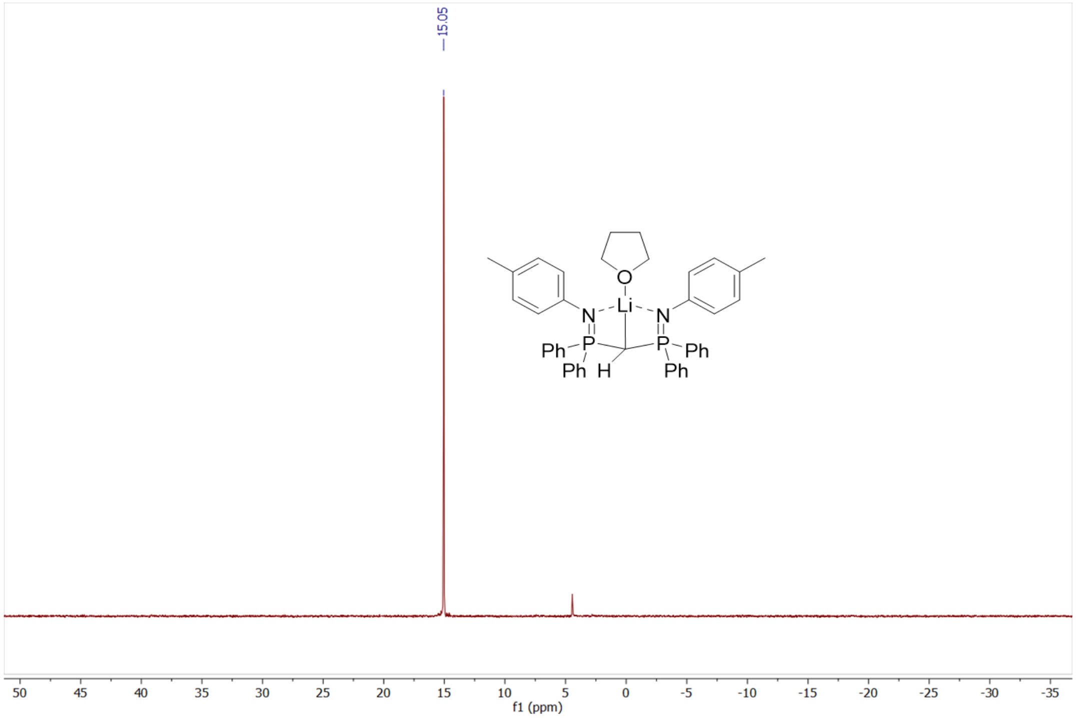

Figure S4. $^{31}P\{^{1}H\}$ NMR spectrum of ligand **L1-Li** (162 MHz, THF-d_8).

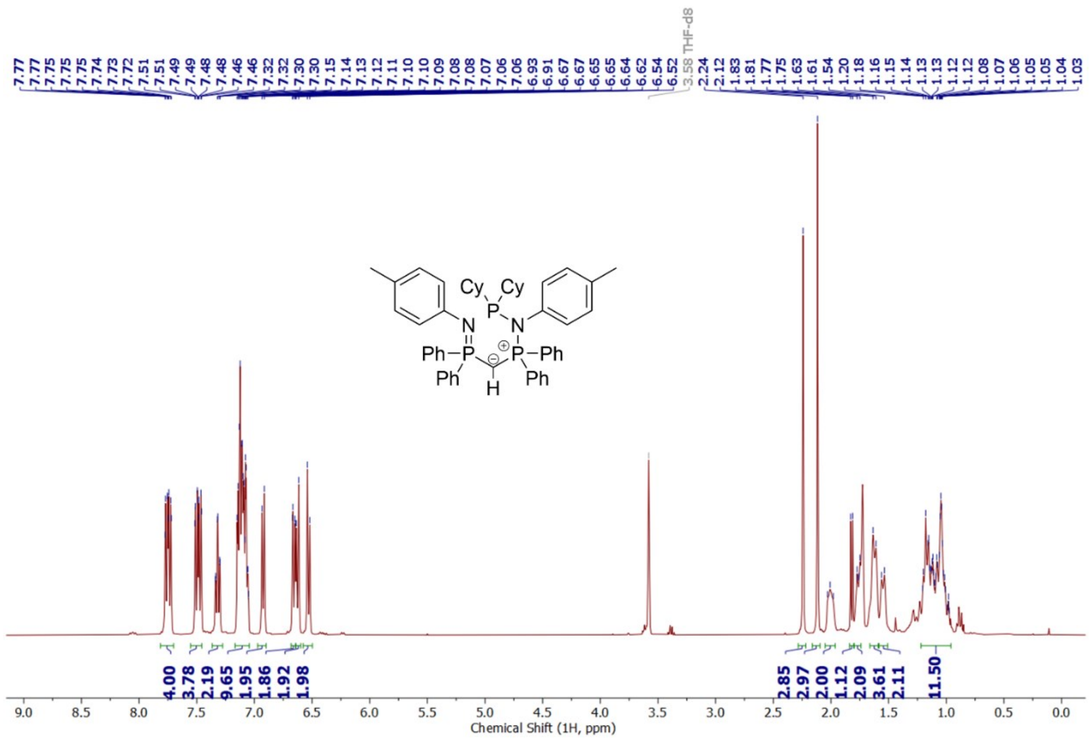

Figure S5 ^{1}H NMR spectrum of ^{Cy}PC(H)N Ligand **L2** (400 MHz, THF-d^{8}).

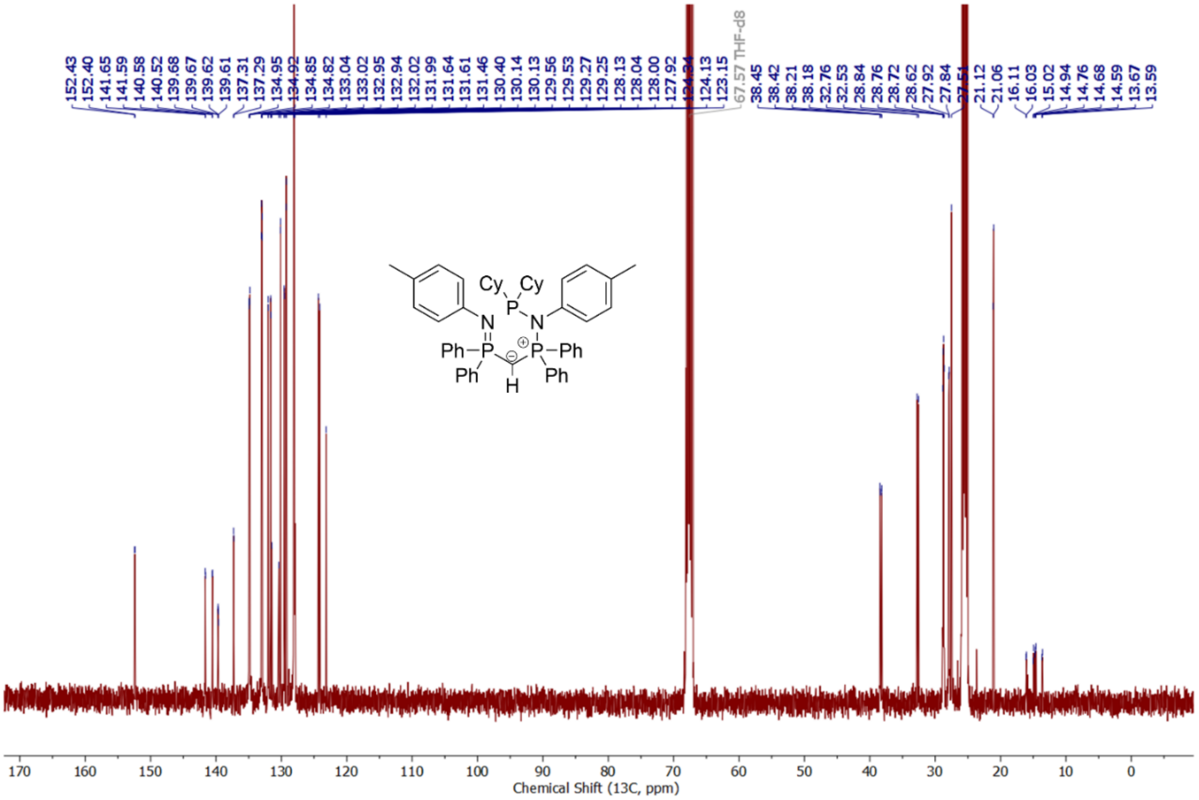

Figure S6. $^{13}C\{^{1}H\}$ NMR spectrum of ^{Cy}PC(H)N Ligand **L2** (101 MHz, THF-d8).

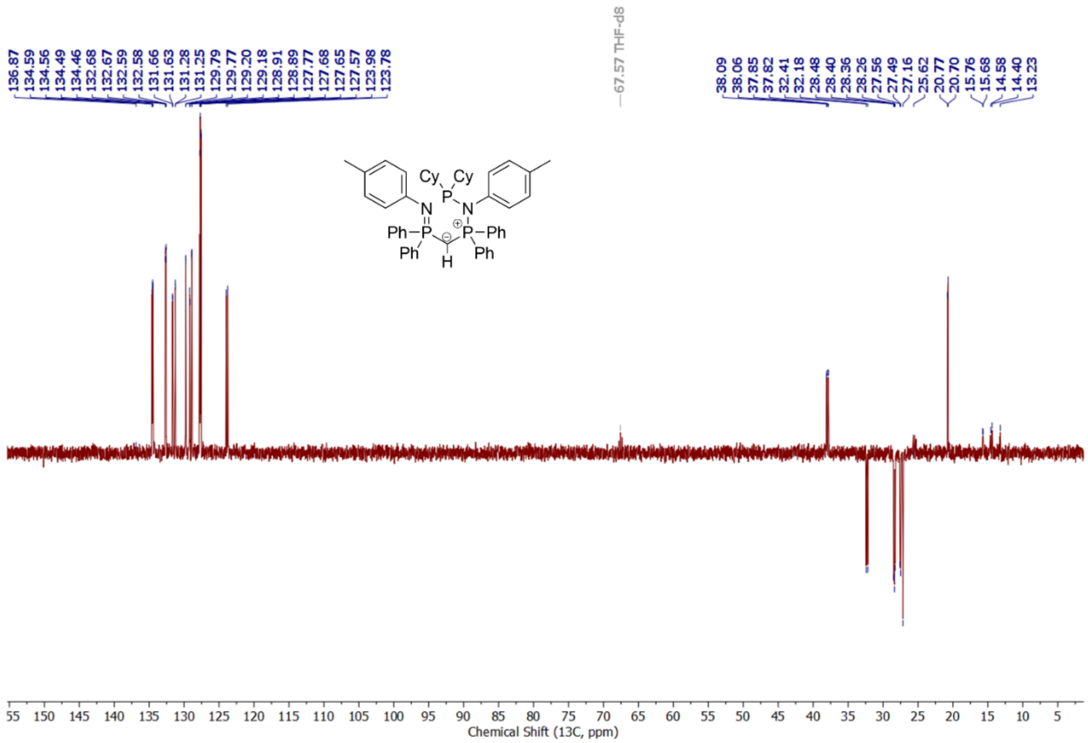

Figure S7. DEPT-135 NMR spectrum of ^{Cy}PC(H)N Ligand **L2** (101 MHz, THF-d8).

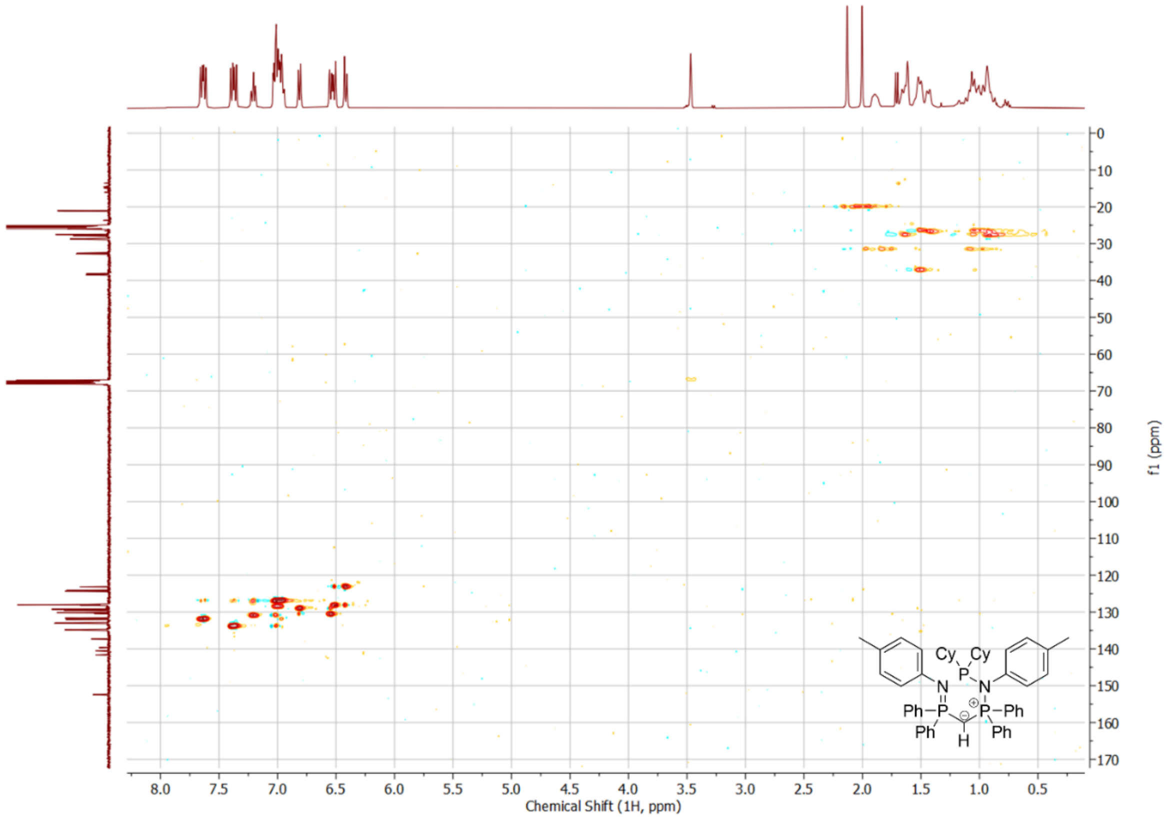

Figure S8. ^{1}H-$^{13}C\{^{1}H\}$ HSQC NMR spectrum of ^{Cy}PC(H)N Ligand **L2** (THF-d_8).

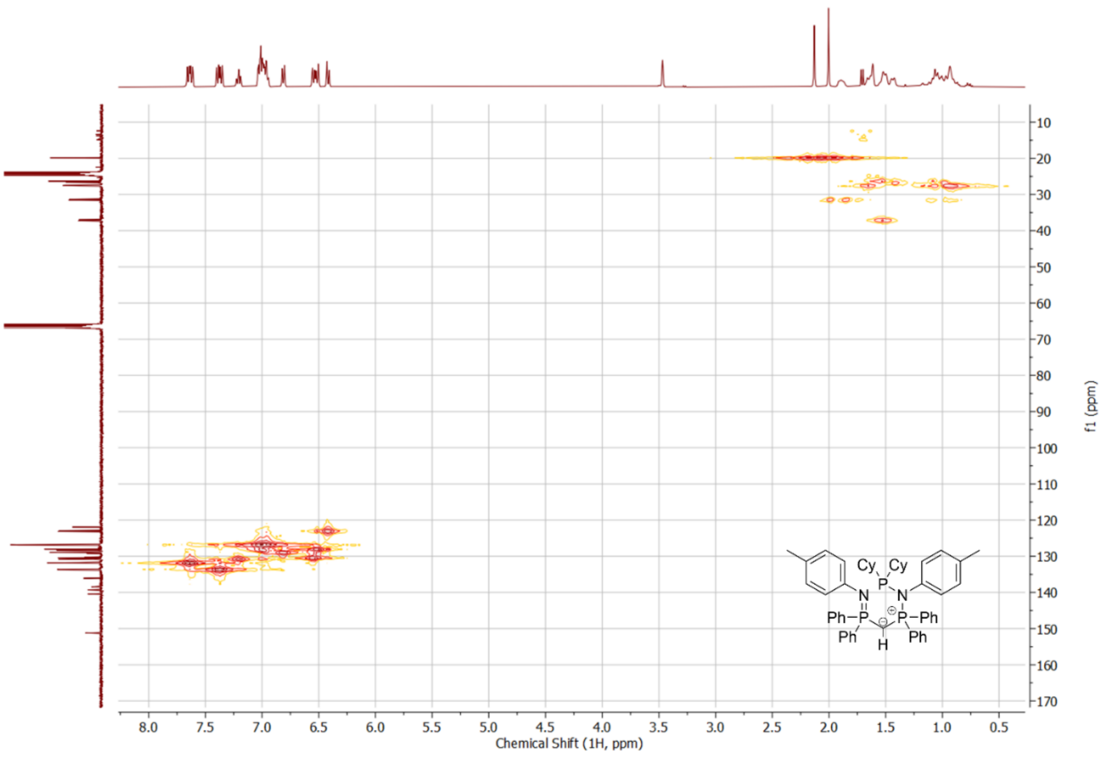

Figure S9. ^{1}H-$^{13}C\{^{1}H\}$ HMQC NMR spectrum of ^{Cy}PC(H)N Ligand **L2** (THF-d_8).

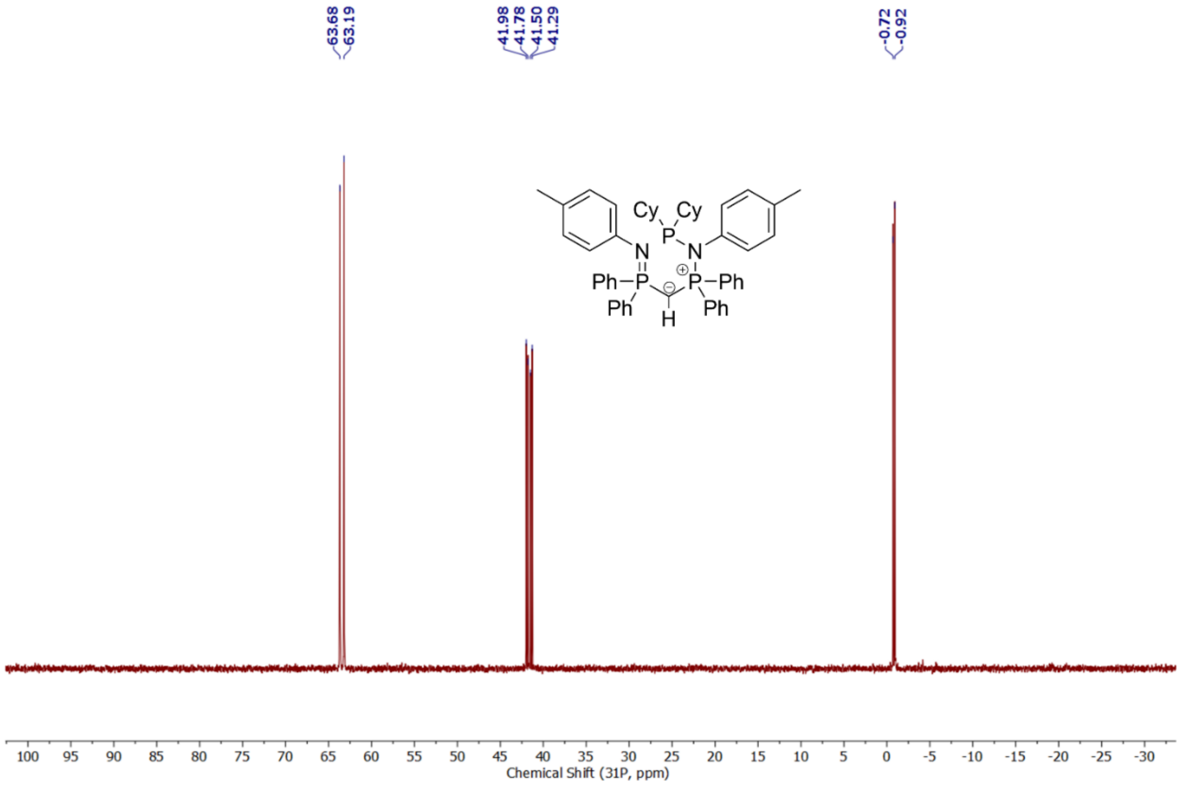

Figure S10. $^{31}P\{^{1}H\}$ NMR spectrum of ^{Cy}PC(H)N Ligand **L2** (162 MHz, THF-d_8).

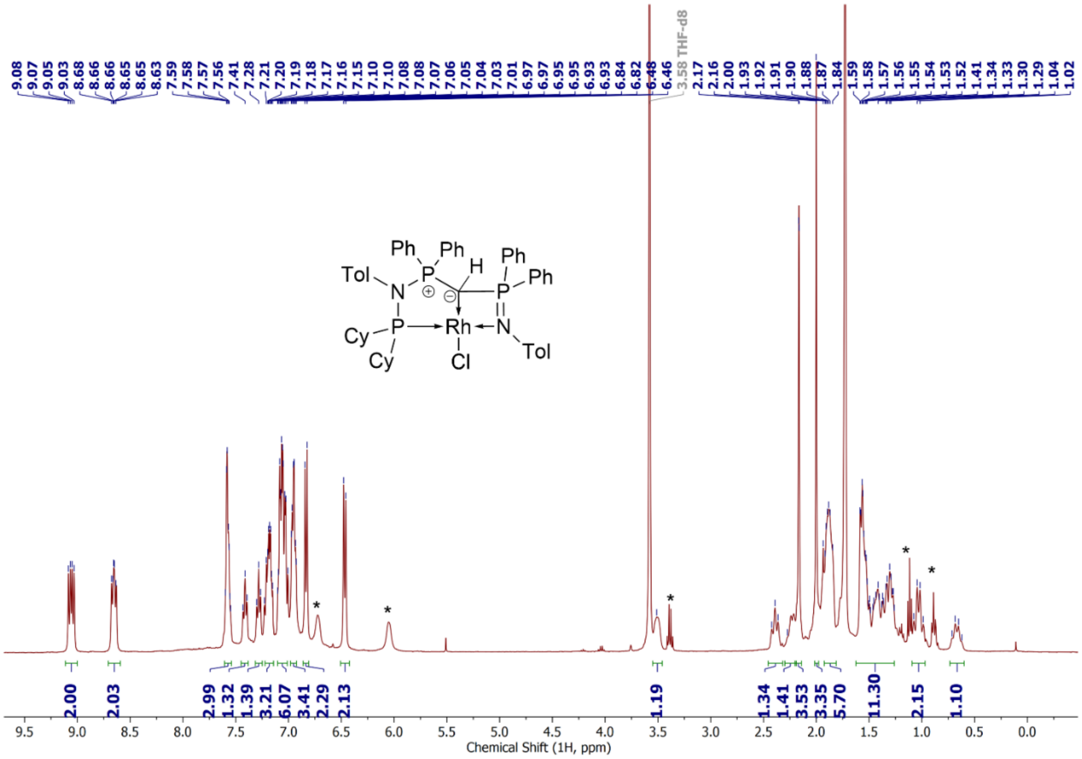

Figure S11. ^{1}H NMR spectrum of complex **1** (400 MHz, THF-d_8). *Traces of solvents and impurities

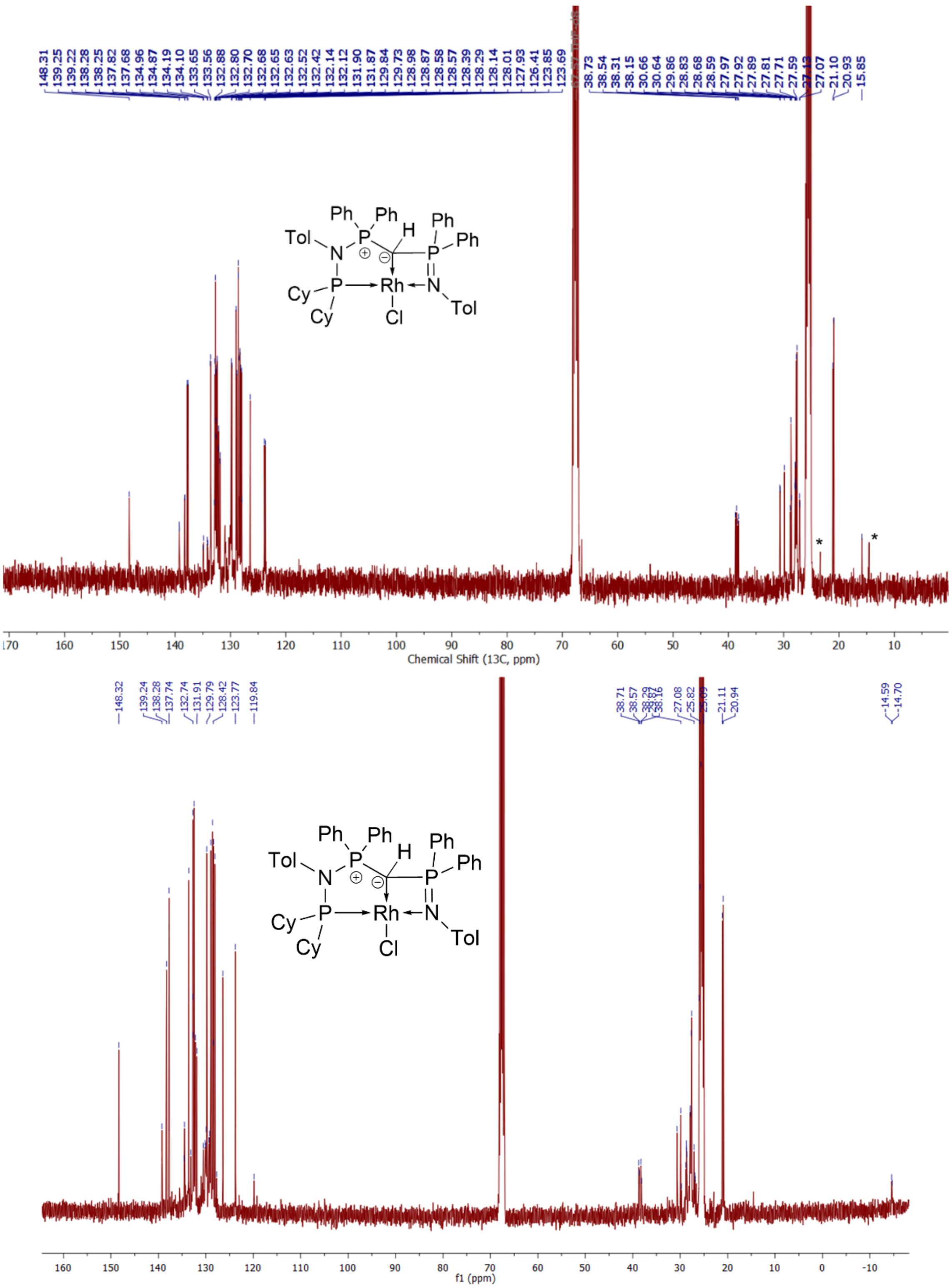

Figure S12. (top) $^{13}C\{^1H\}$ NMR spectrum of complex **1** (101 MHz, THF-d_8) *Traces of pentane. (bottom) $^{13}C\{^1H, ^{31}P\}$ NMR spectrum of complex **1** (101 MHz, THF-d_8) to show the signal of the central carbon atom C27 at -14.65 ppm that could not be observed using $^{13}C\{^1H\}$ NMR.

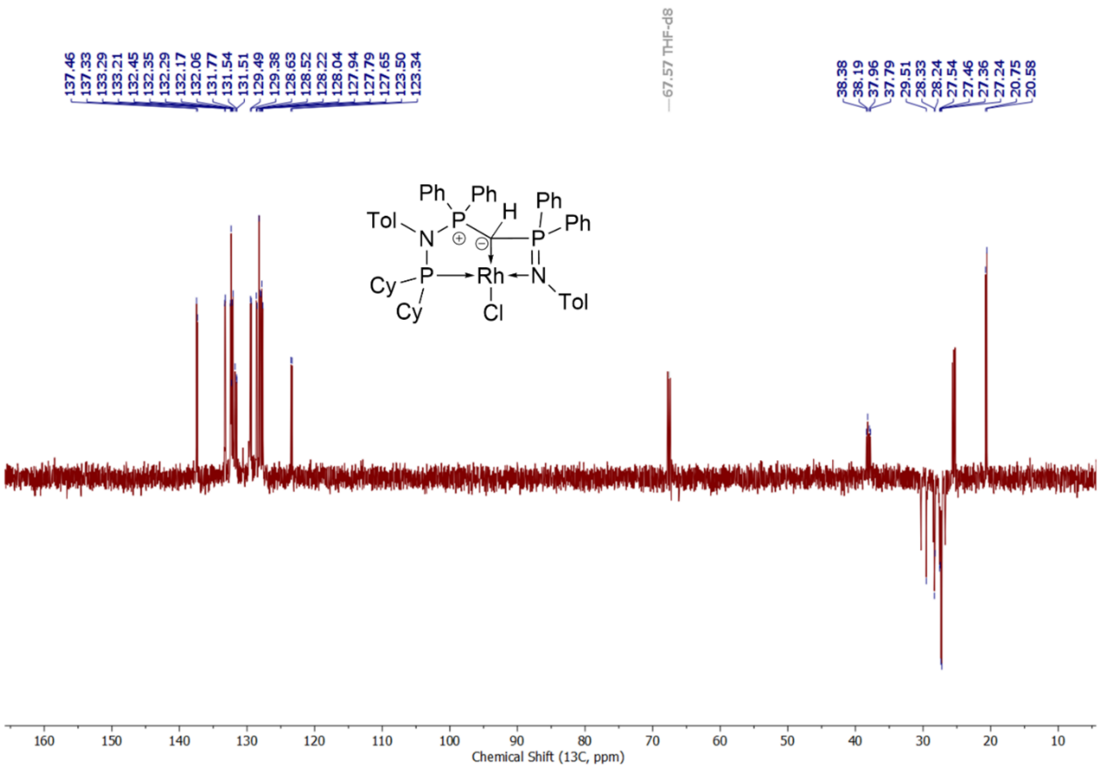

Figure S13. DEPT-135 NMR spectrum of complex **1** (101 MHz, THF-d8).

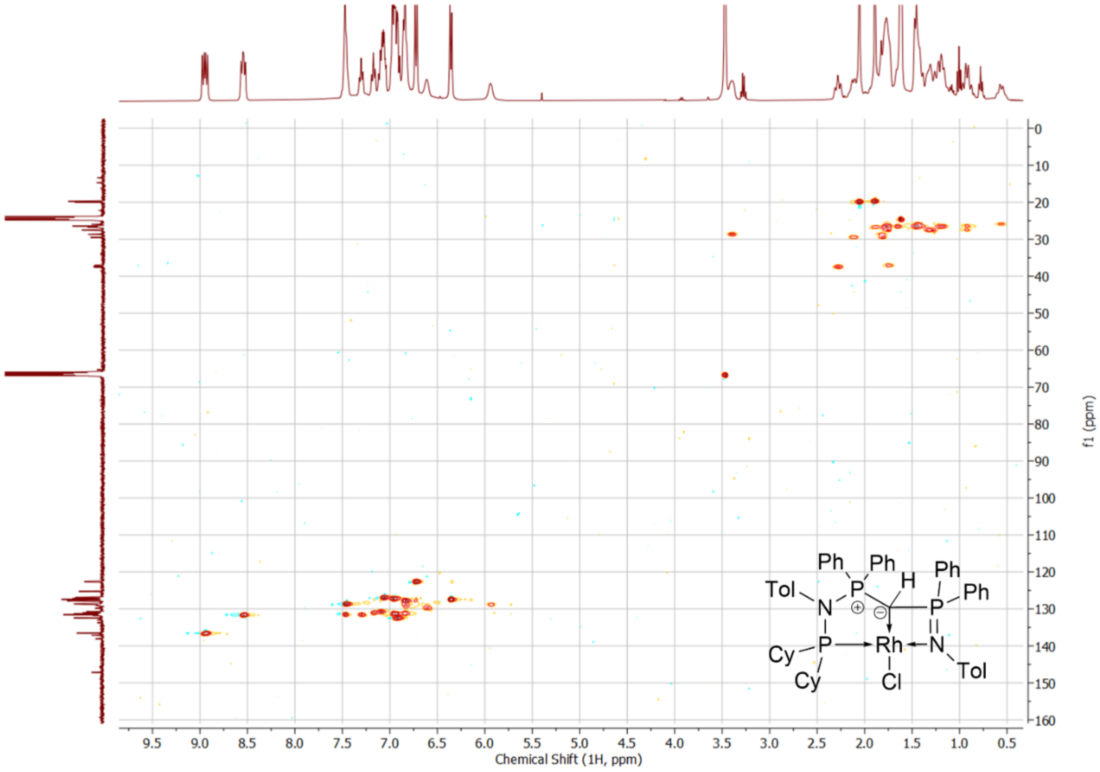

Figure S14. 1H-13C HSQC NMR spectrum of complex **1** (THF-d8).

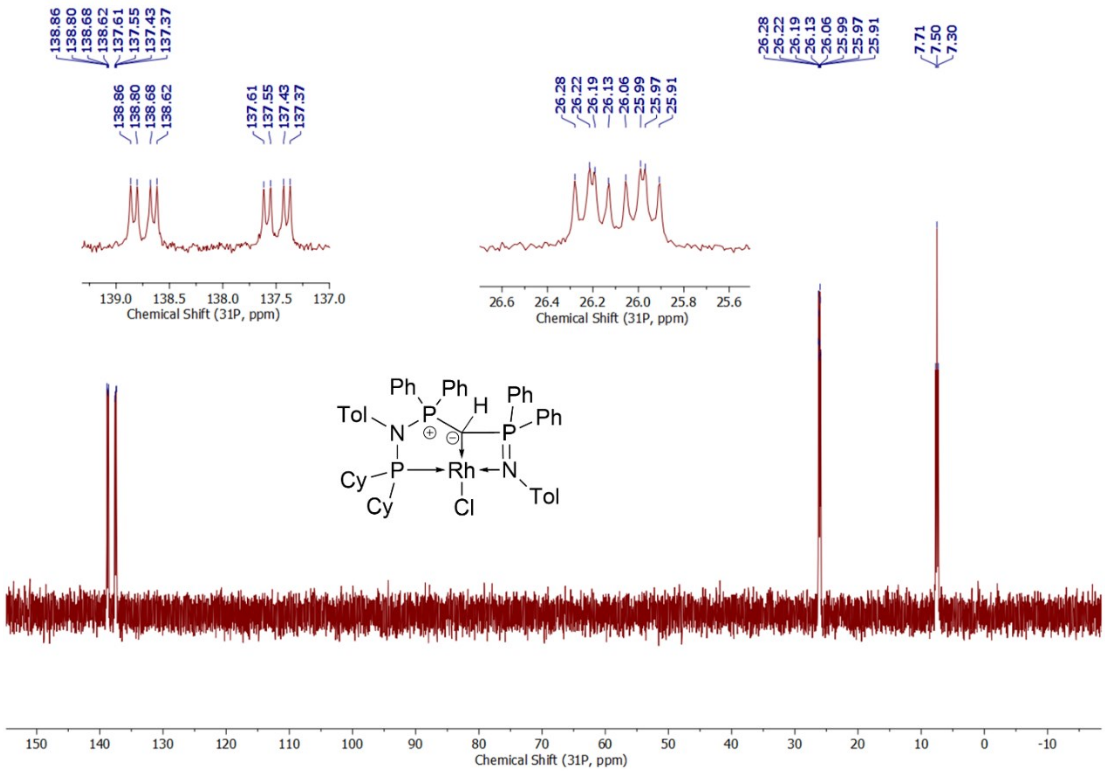

Figure S15. $^{31}P\{^{1}H\}$ NMR spectrum of complex **1** (162 MHz, THF-d8).

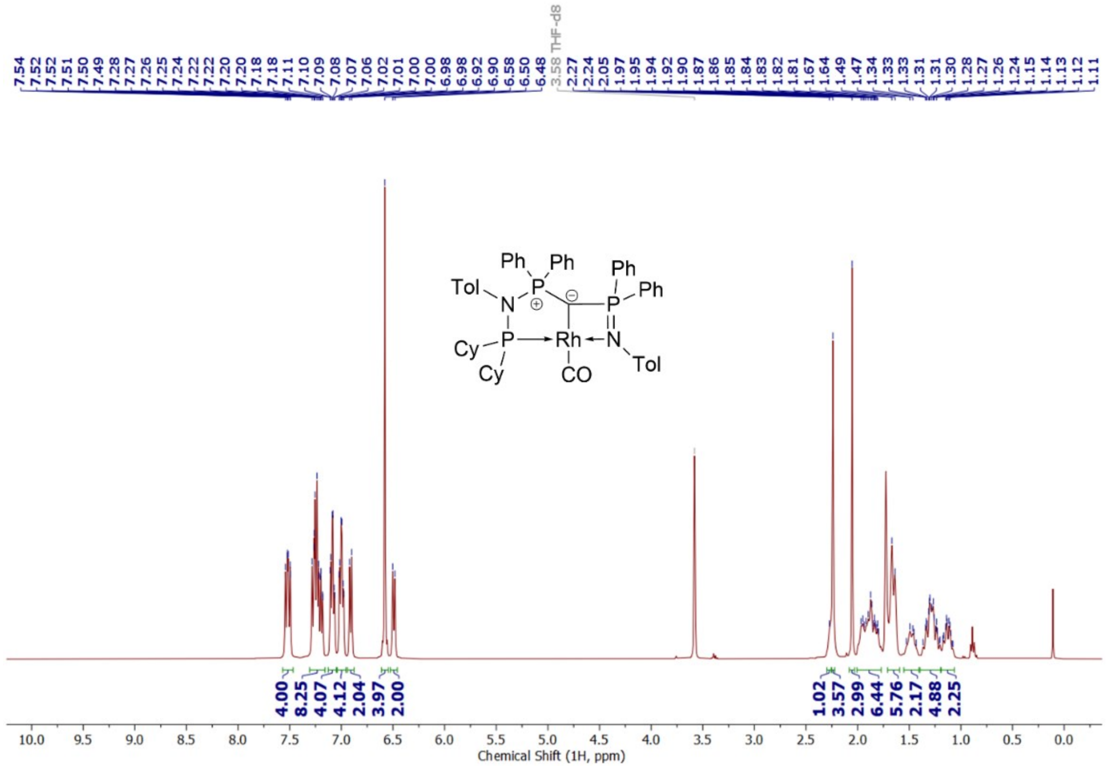

Figure S16. ^{1}H NMR spectrum of complex **2** (400 MHz, THF-d8).

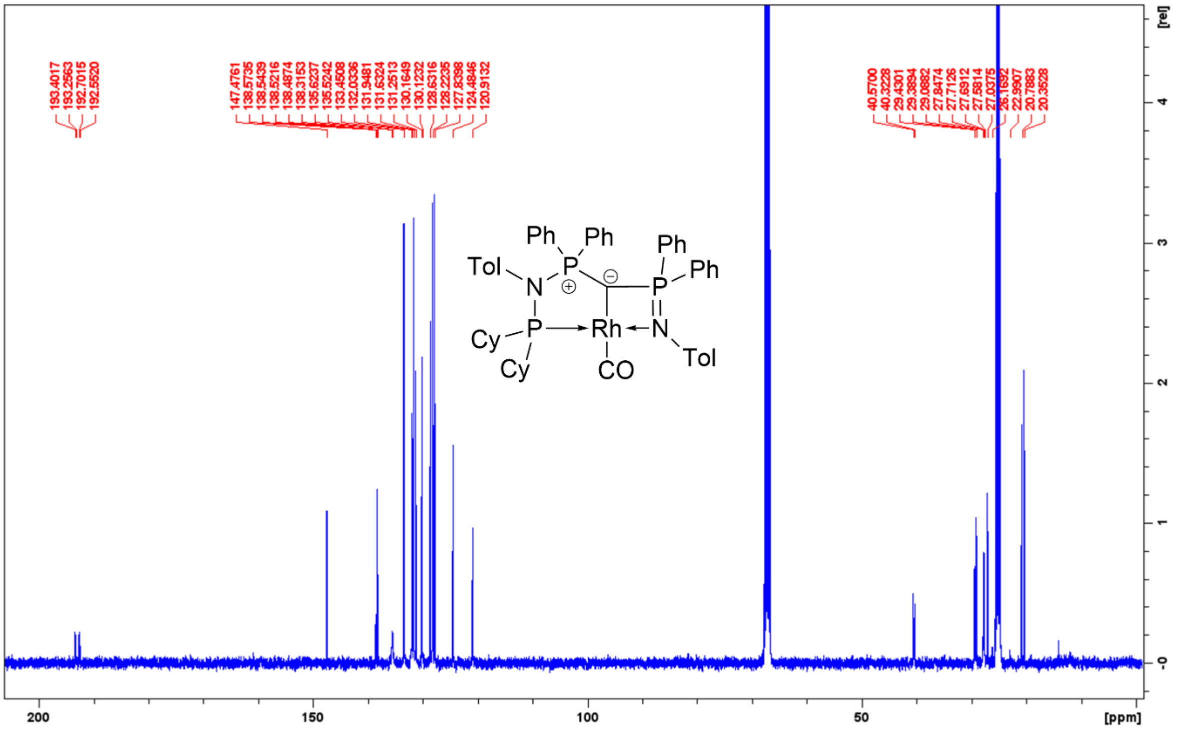

Figure S17. $^{13}C\{^{1}H\}$ NMR spectrum of complex **2** (101 MHz, THF-d_8).

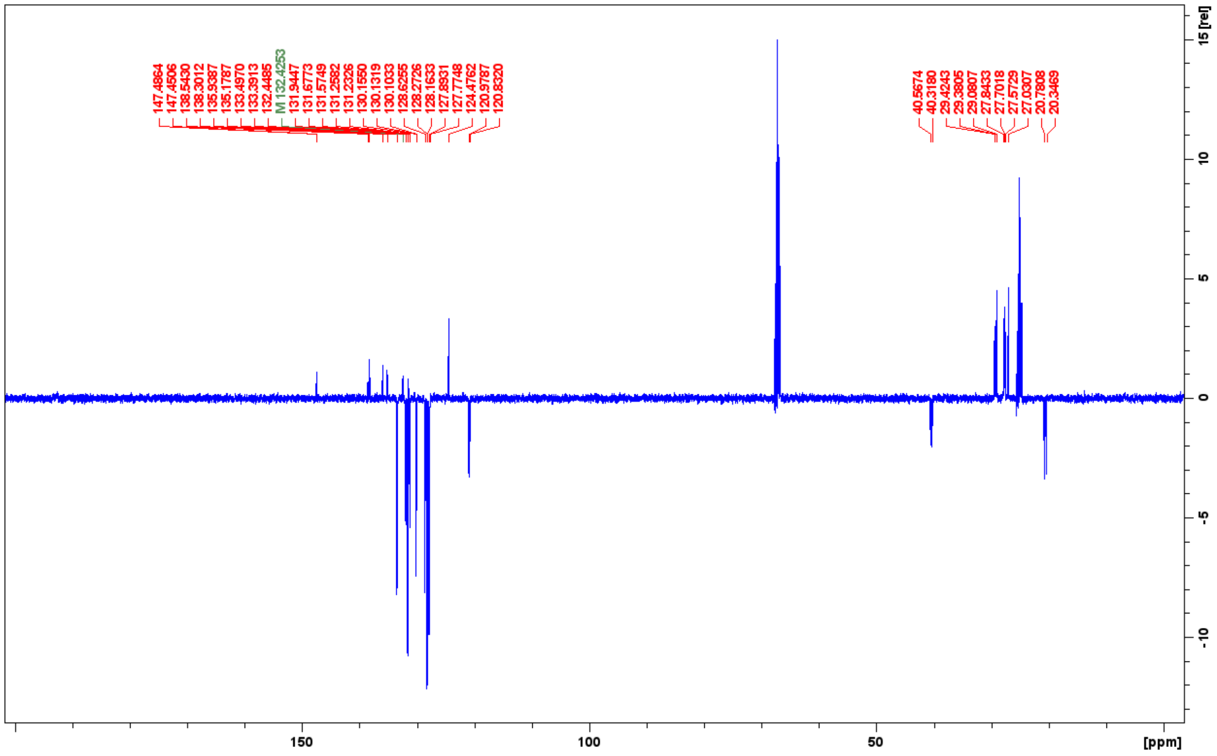

Figure S18. APT NMR spectrum of complex **2** (101 MHz, THF-d_8). Quaternary C and CH_2 are positive, CH and CH_3 are negative.

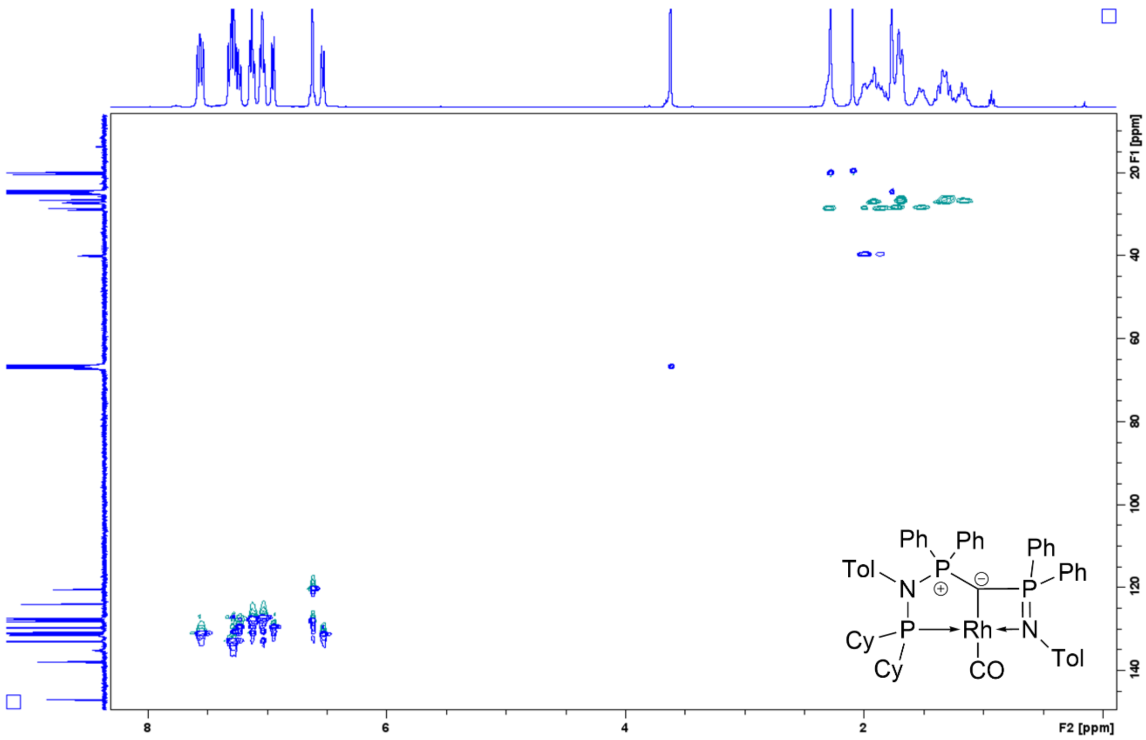

Figure S19. ^{1}H-^{13}C HSQC NMR spectra of complex **2** (THF-d_8).

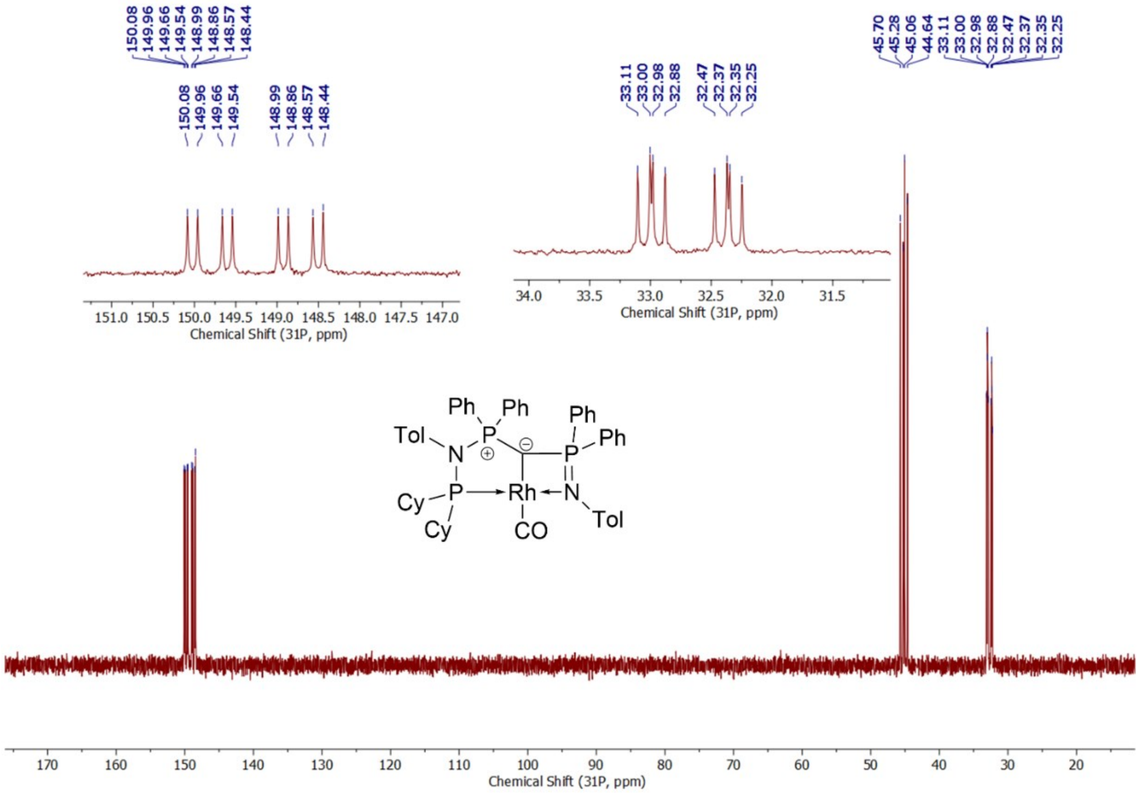

Figure S20. $^{31}P\{^{1}H\}$ NMR spectra of complex **2** (162 MHz, THF-d_8).

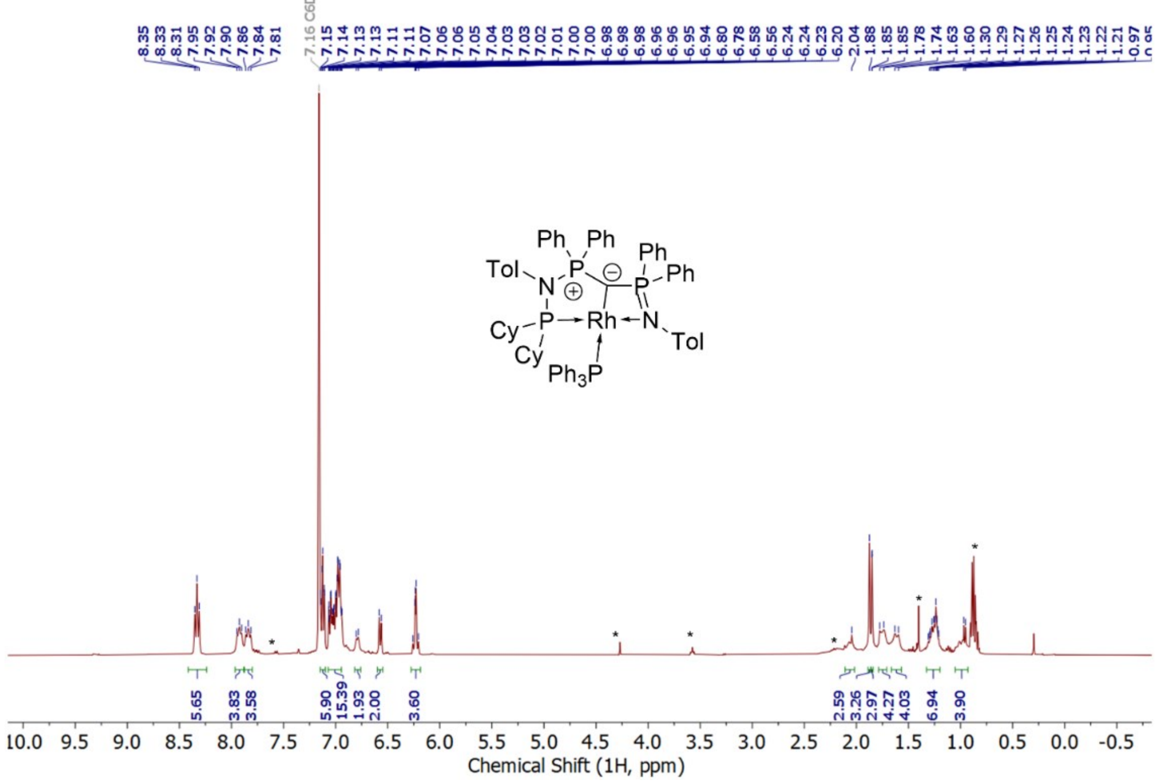

Figure S21. ^{1}H NMR spectrum of complex **3** (400 MHz, C_6D_6). * traces of solvents and an halogenated ligand side-product.

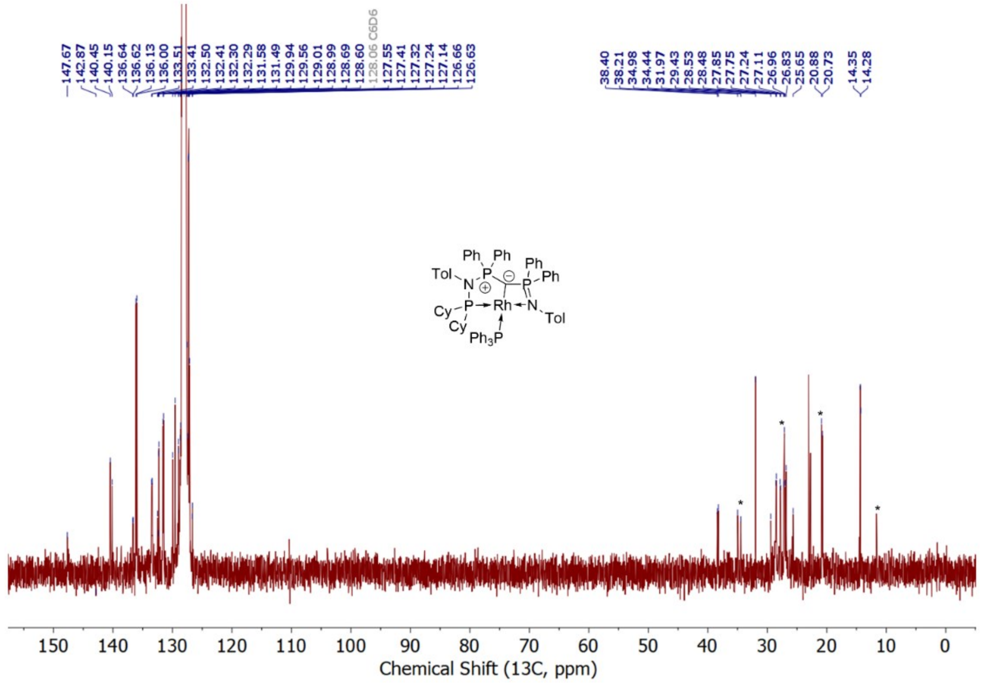

Figure S22. $^{13}C\{^{1}H\}$ NMR spectrum of complex **3** (101 MHz, C_6D_6). *Traces of solvents, and an halogenated ligand side-product.

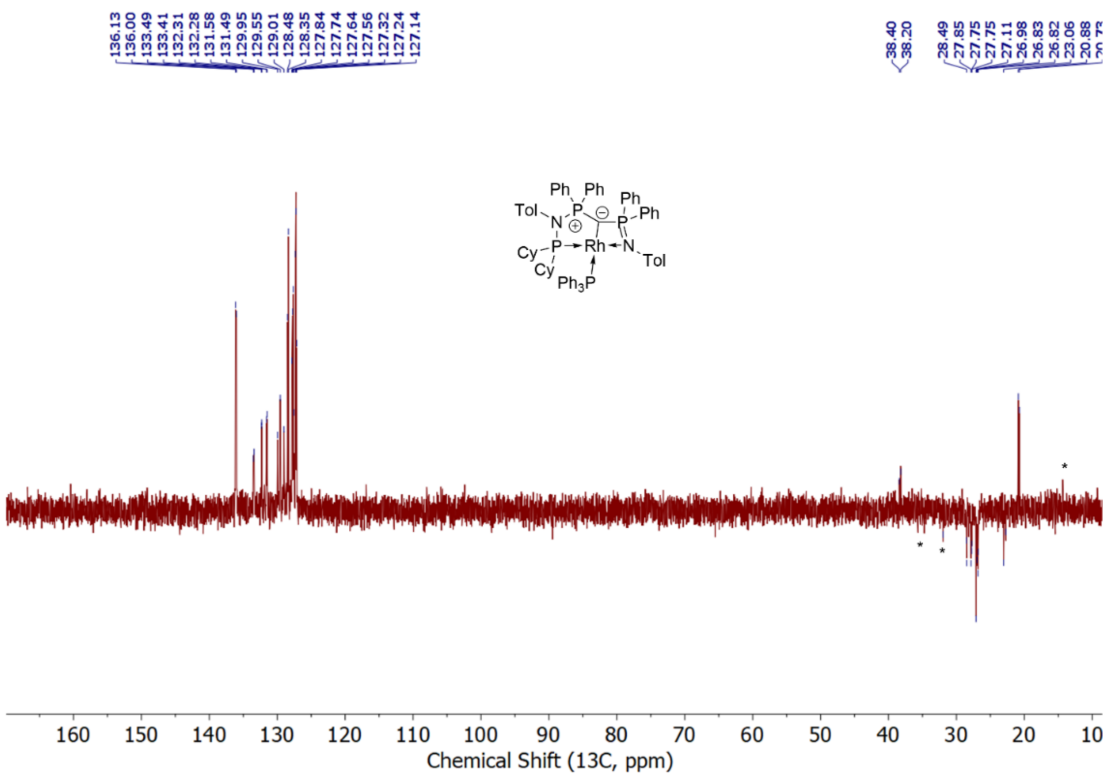

Figure S23. DEPT-135 NMR spectrum of complex **3** (101 MHz, C_6D_6). *Traces of solvents

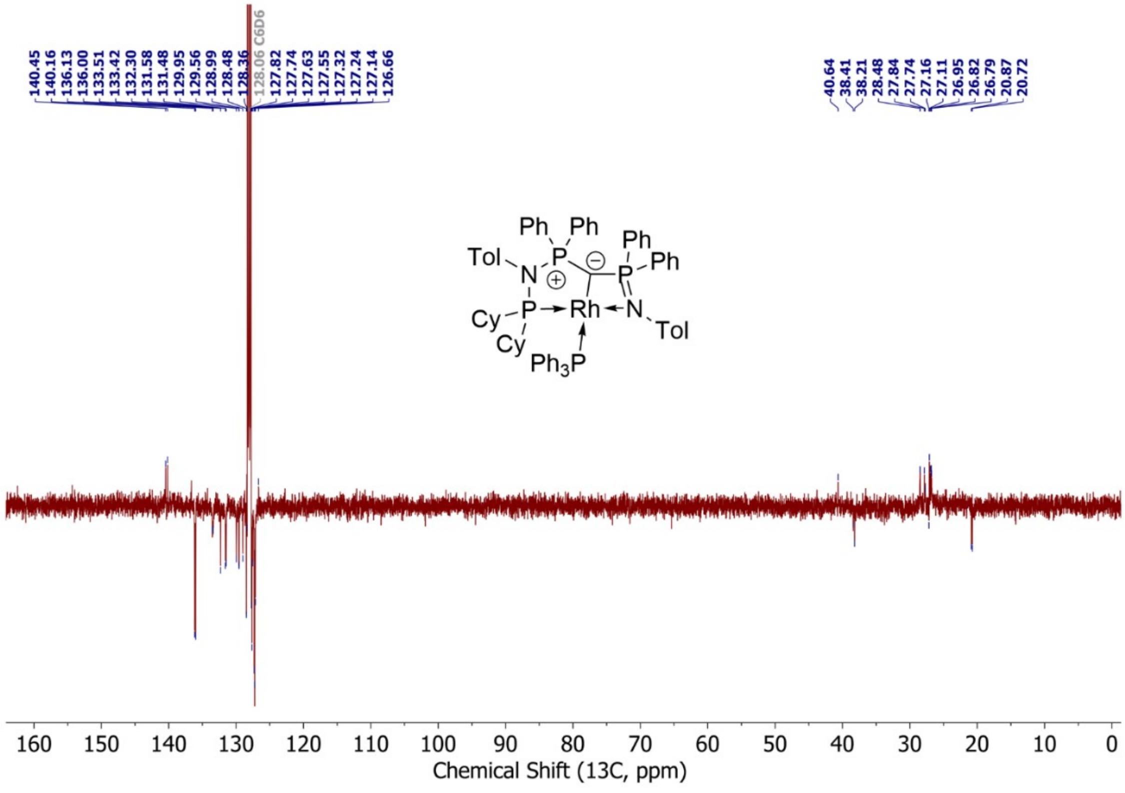

Figure S24. APT NMR spectrum of complex **3** (101 MHz, C_6D_6)

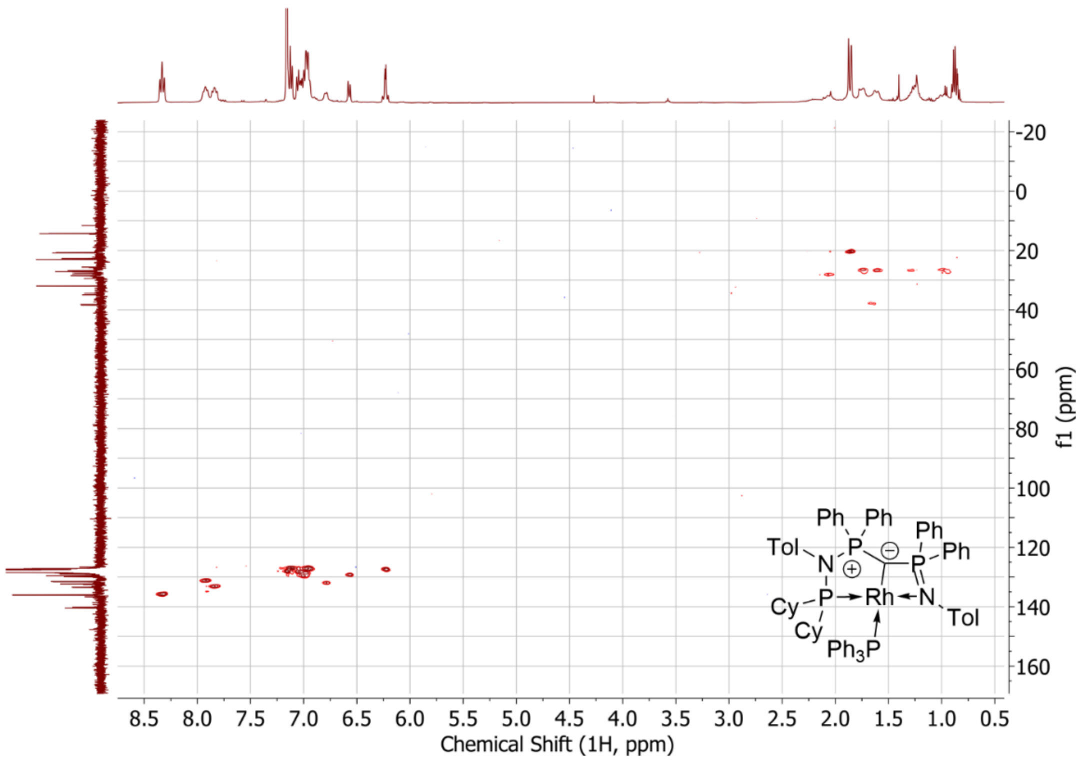

Figure S25. ^{13}C-^{1}H HSQC NMR spectrum of complex **3** (C_6D_6)

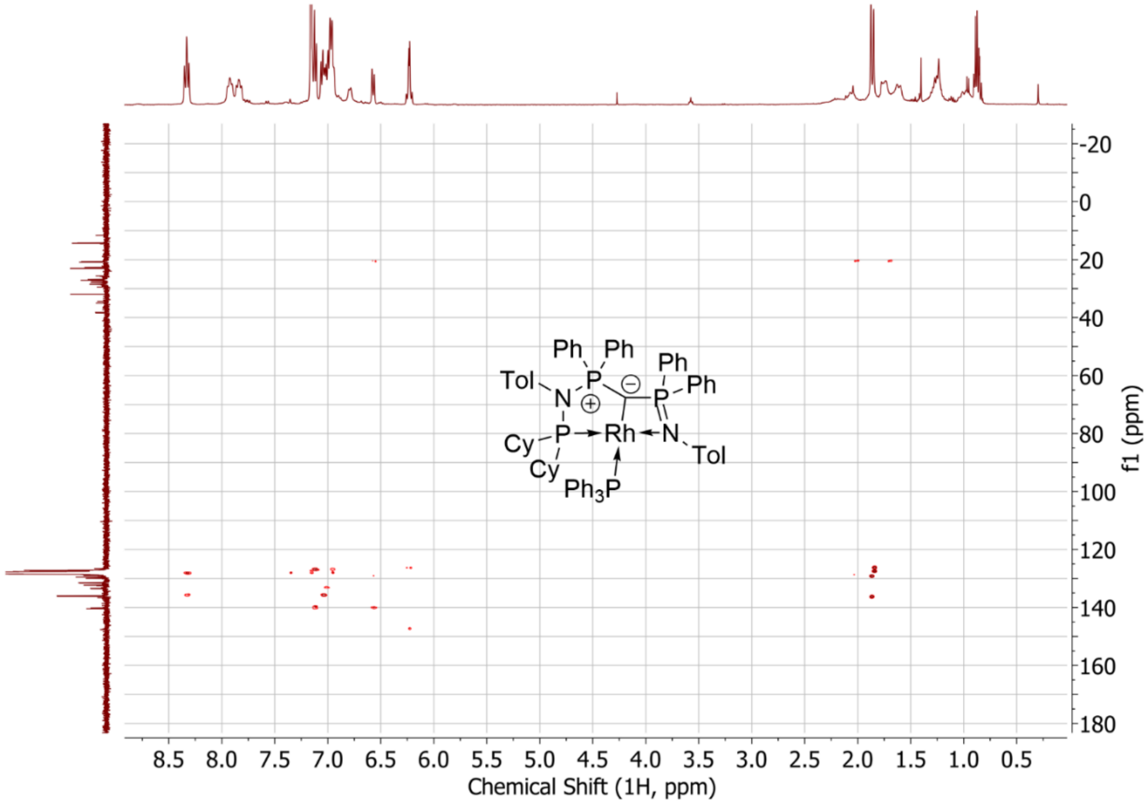

Figure S26. ^{13}C-^{1}H HMBC NMR spectrum of complex **3** (C_6D_6)

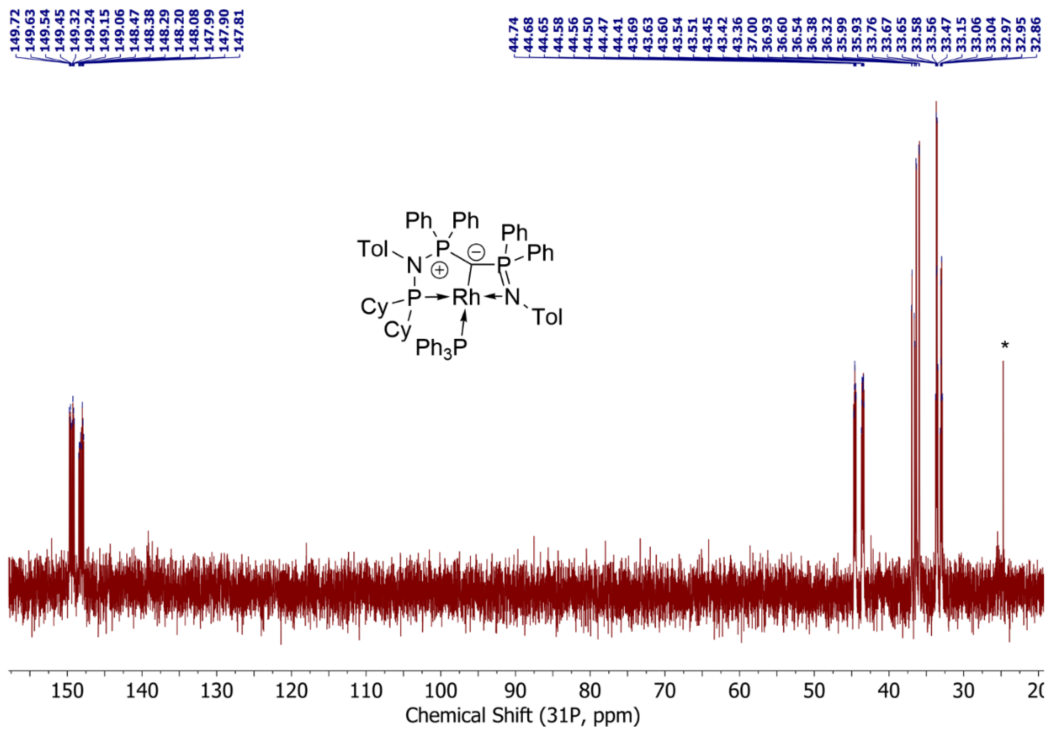

Figure S27. $^{31}P\{^1H\}$ NMR spectrum of complex **3** (162 MHz, C_6D_6). * Traces of BIPM-Cl

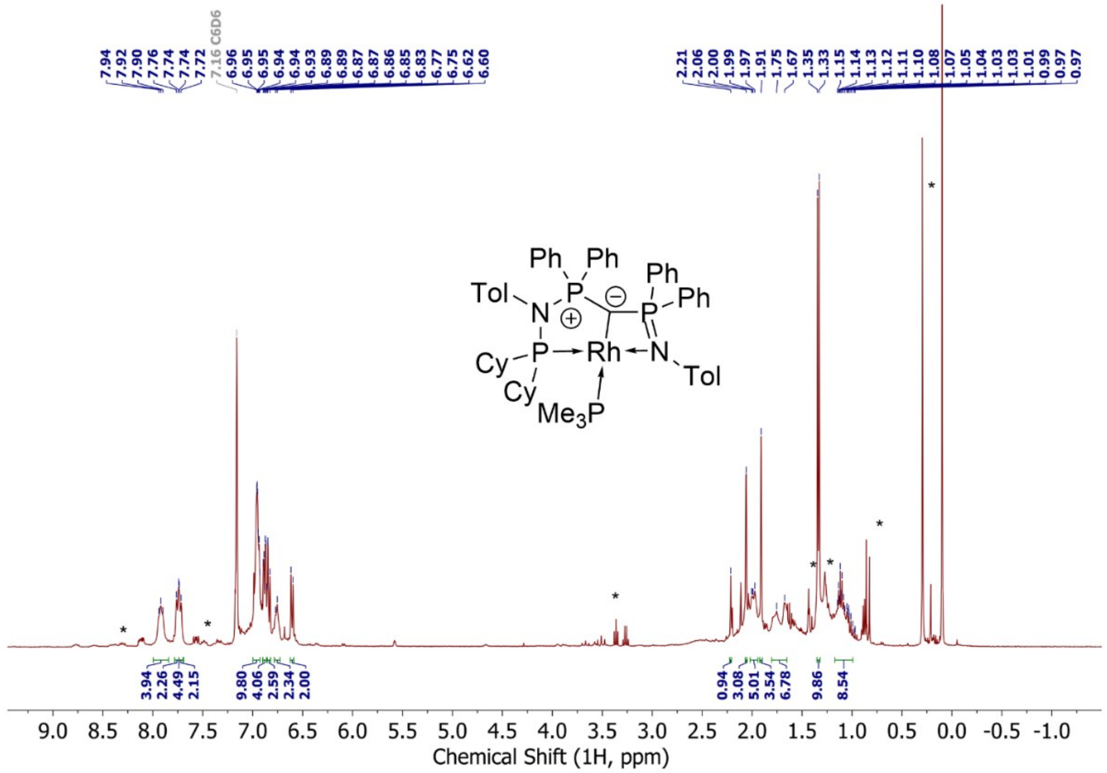

Figure S28. ^1H NMR spectrum of complex **4** (400 MHz, C_6D_6). * Traces of solvents and decomposition products

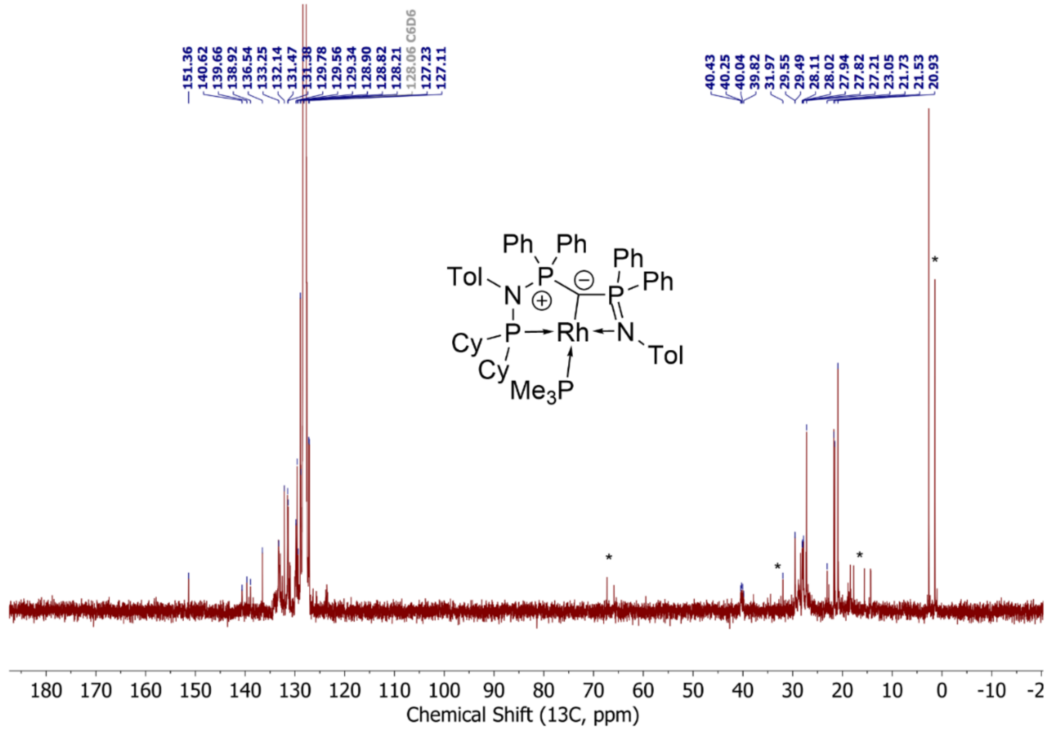

Figure S29. $^{13}C\{^{1}H\}$ NMR spectrum of complex **4** (101 MHz, C_6D_6). *Traces solvent and decomposition products

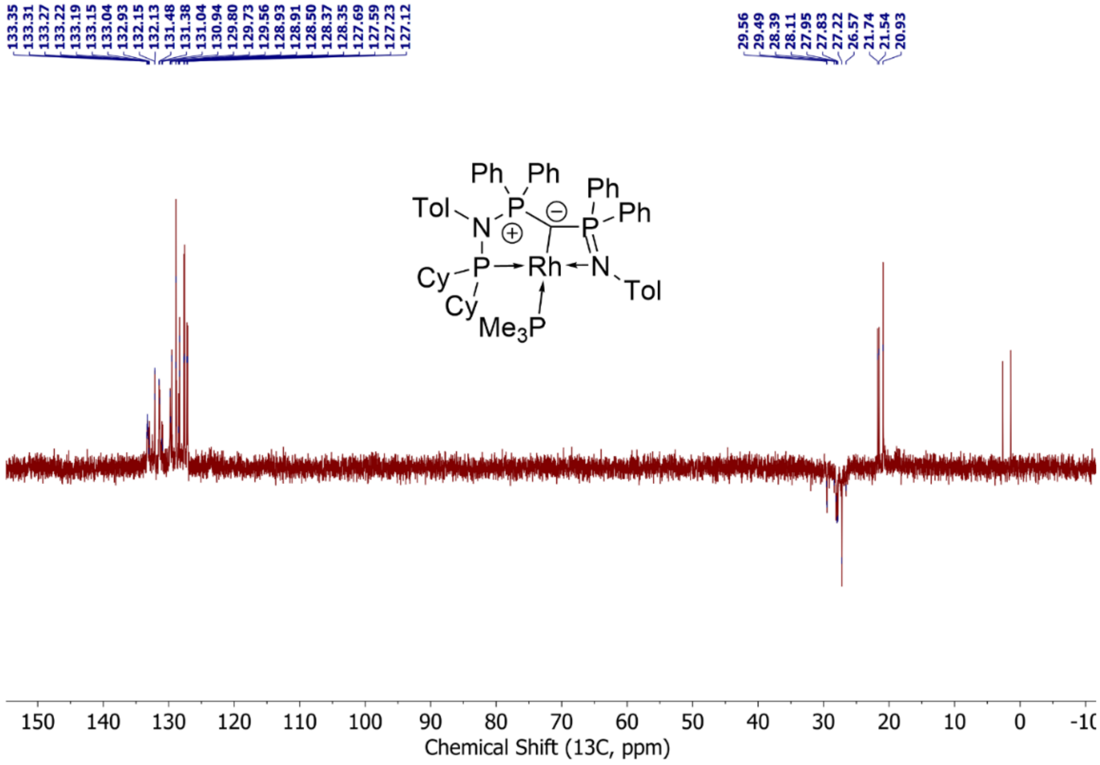

Figure S30. DEPT-135 NMR spectrum of complex **4** (101 MHz, C_6D_6).

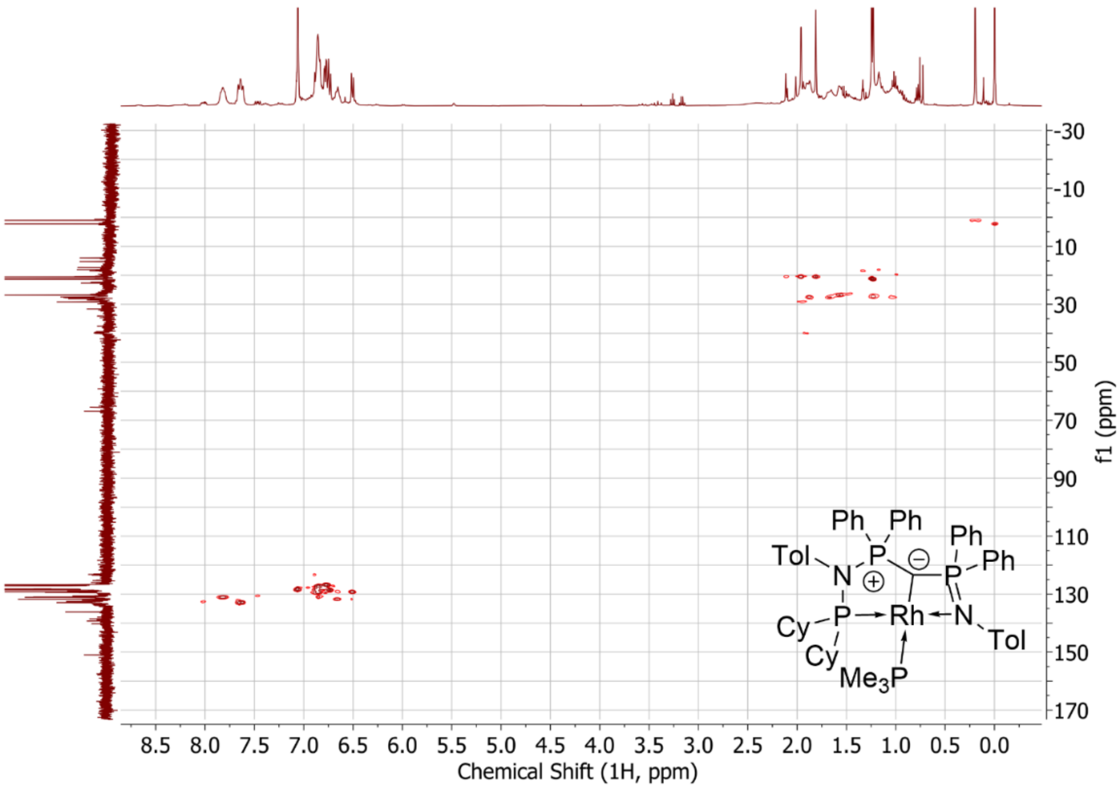

Figure S31. ^{1}H-^{13}C HSQC NMR spectrum of complex **4** (C_6D_6).

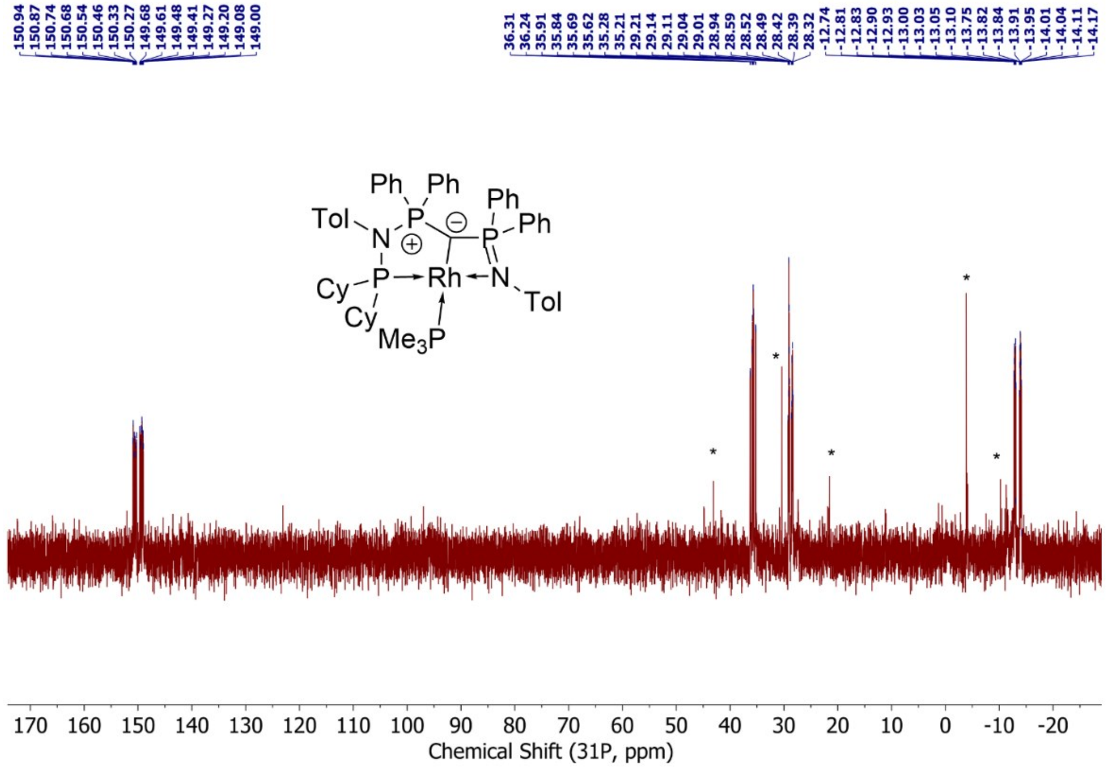

Figure S32. $^{31}P\{^{1}H\}$ NMR spectrum of complex **4** (400 MHz, C_6D_6). *Traces of decomposition products

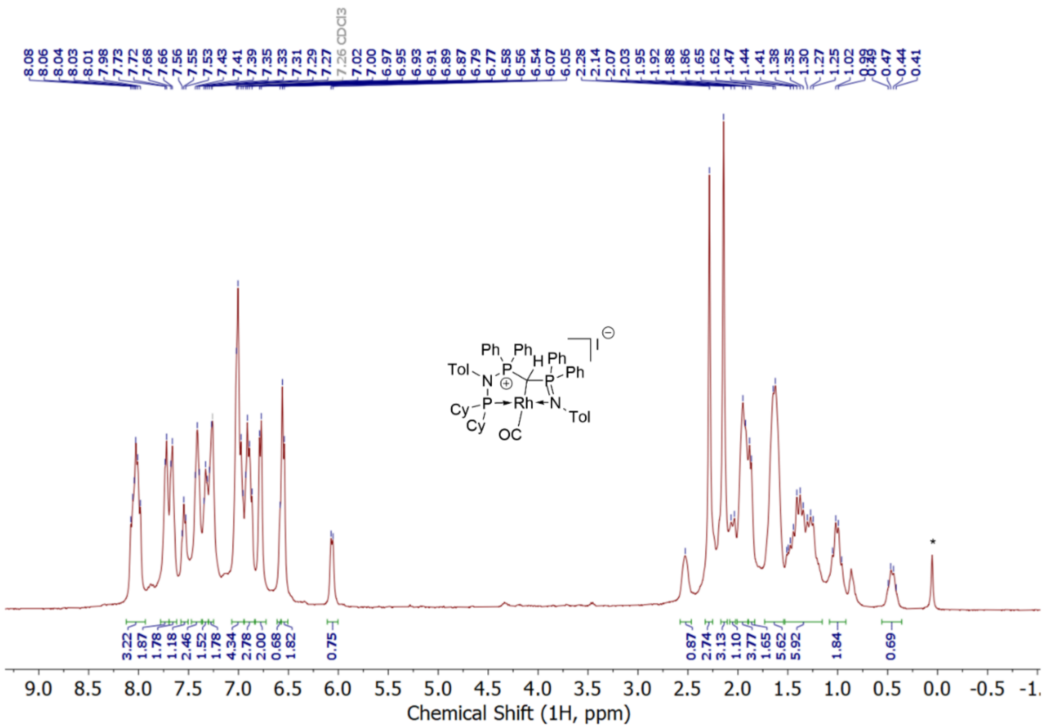

Figure S33. ^{1}H NMR spectrum of complex **5** (400 MHz, $CDCl_3$). *Traces of Si grease

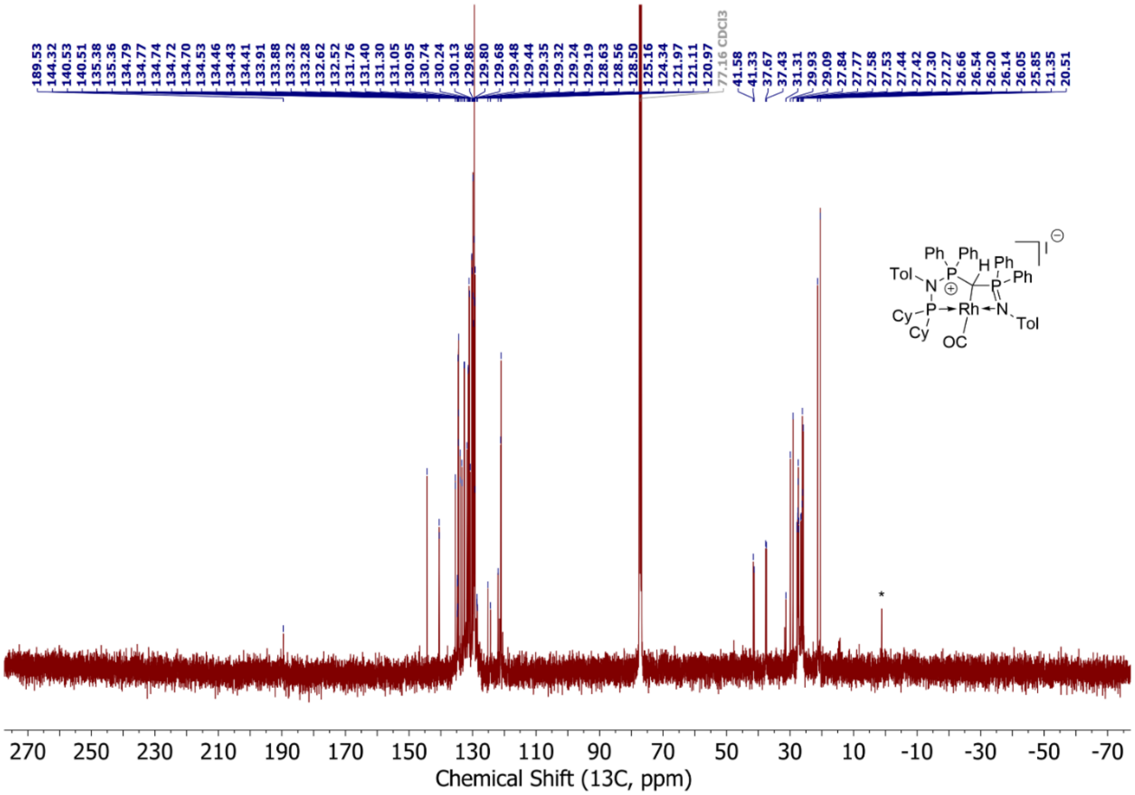

Figure S34. $^{13}C\{^{1}H\}$ NMR spectrum of complex **5** (101 MHz, $CDCl_3$). *Traces of Si grease

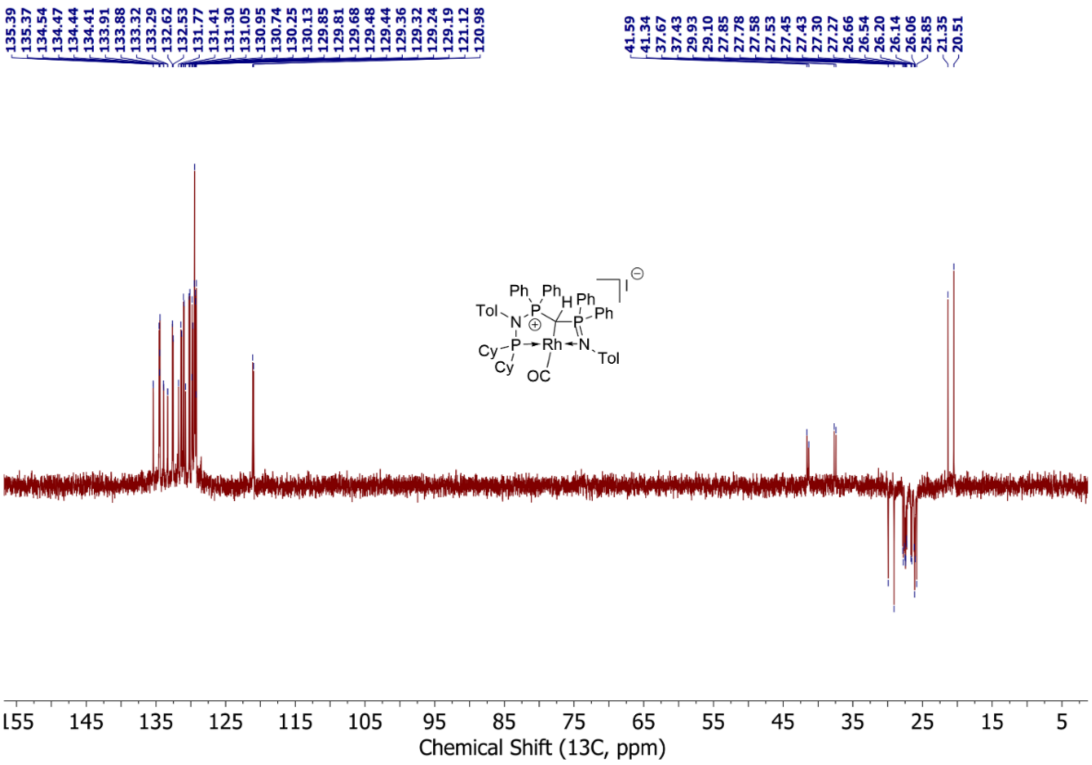

Figure S35. DEPT-135 NMR spectrum of complex **5** (101 MHz, $CDCl_3$)

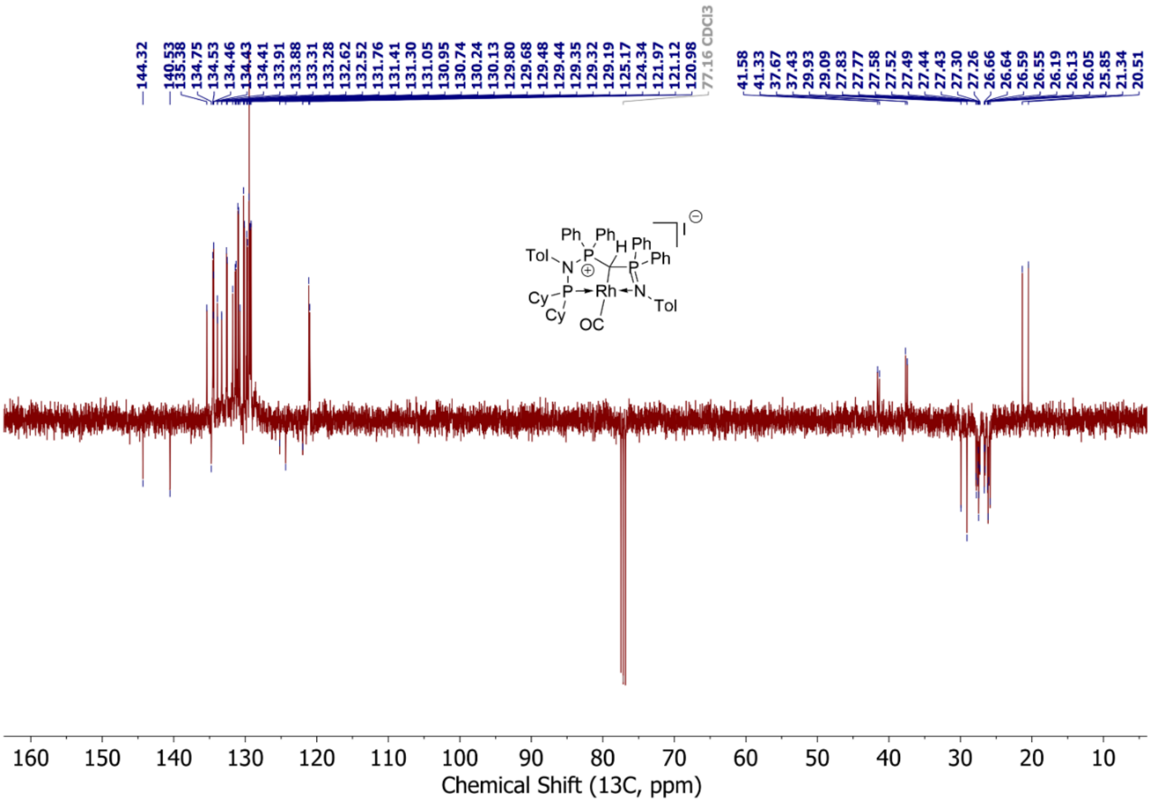

Figure S36. APT NMR spectrum of complex **5** (101 MHz, $CDCl_3$)

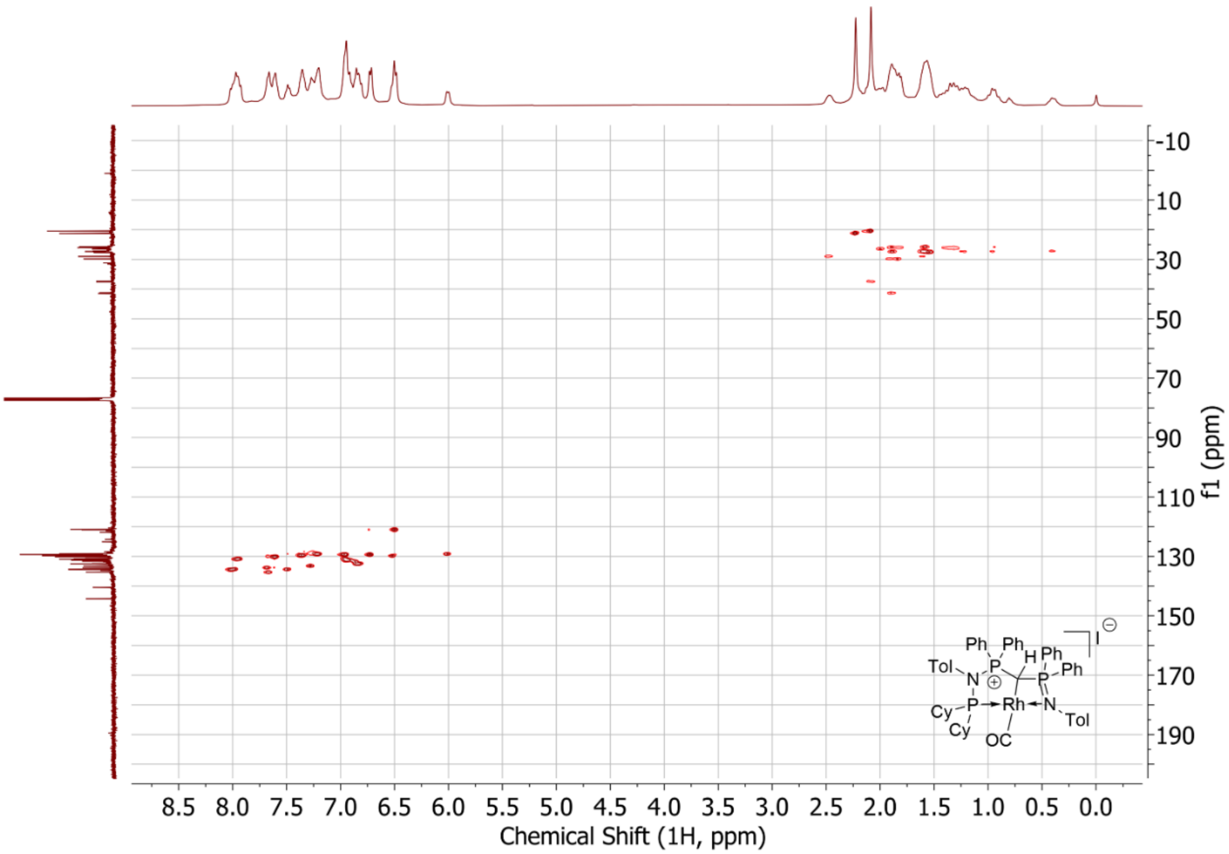

Figure S37. ^{13}C-^{1}H HSQC NMR spectrum of complex **5** ($CDCl_3$)

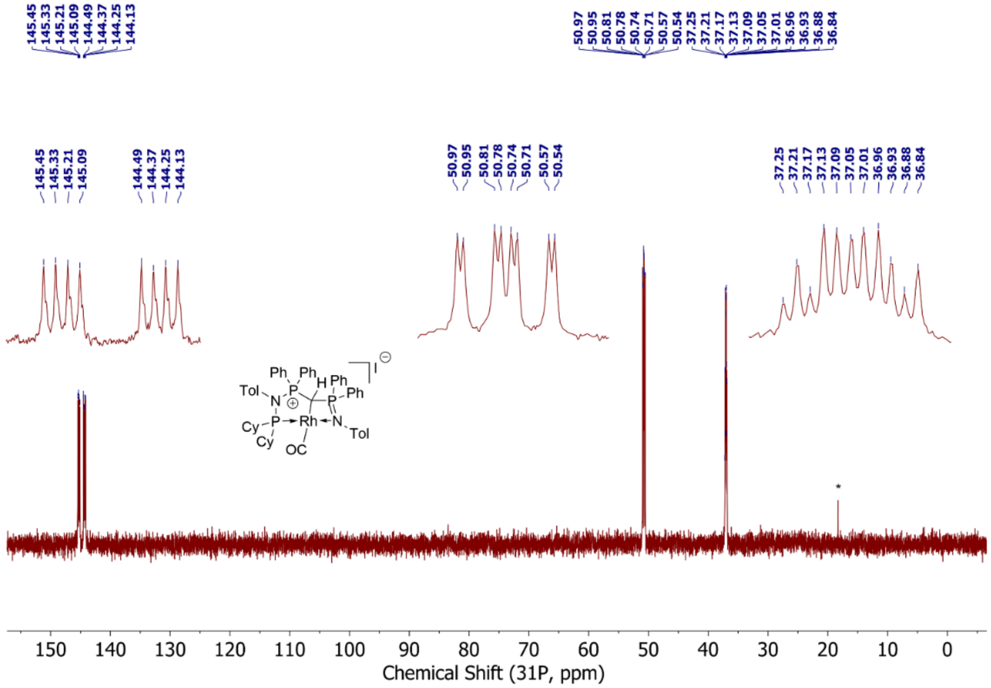

Figure S38. $^{31}P\{^{1}H\}$ NMR spectrum of complex **5** (162 MHz, $CDCl_3$). *Traces of BIPM-I ligand

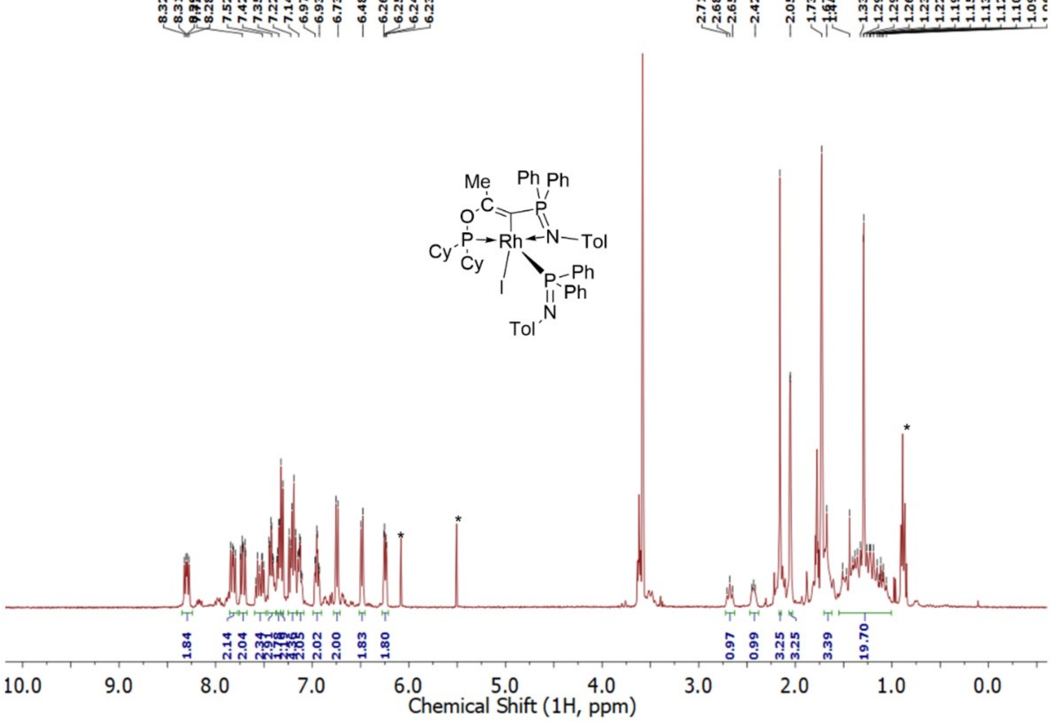

Figure S39. ^{1}H NMR spectrum of complex **6** (400 MHz, THF-d_8). *Traces of solvent and unidentified product.

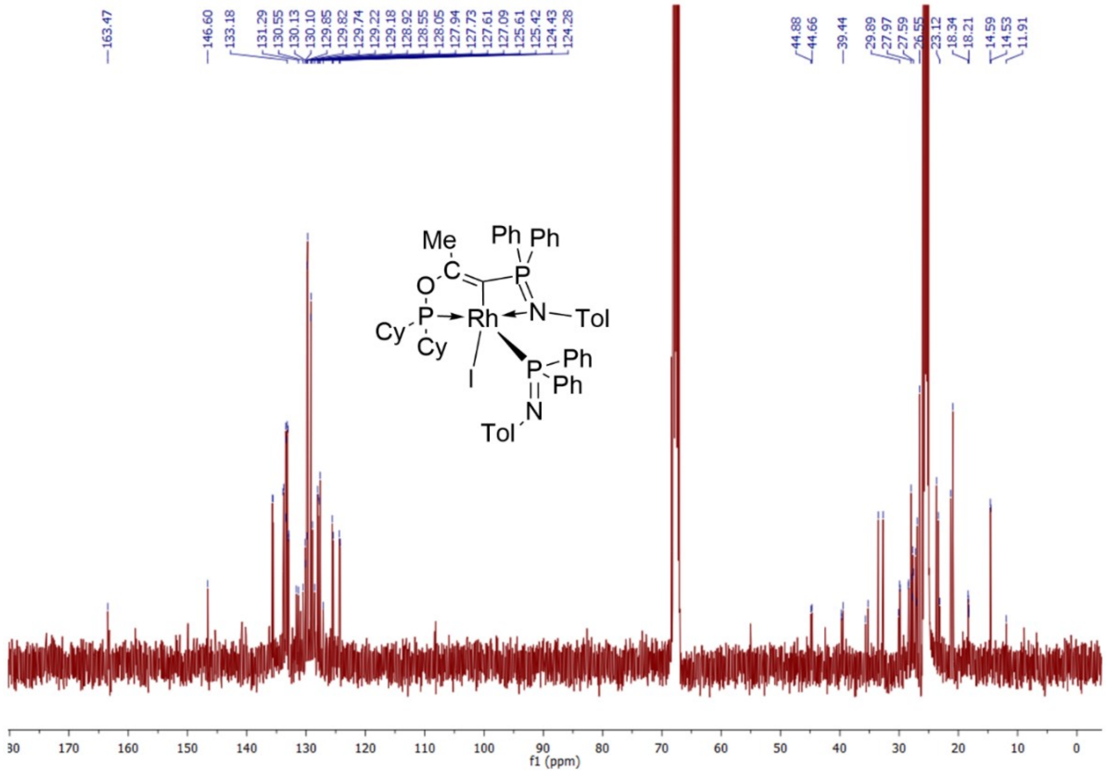

Figure S40. $^{13}C\{^{1}H\}$ NMR spectrum of complex **6** (101 MHz, THF-d_8)

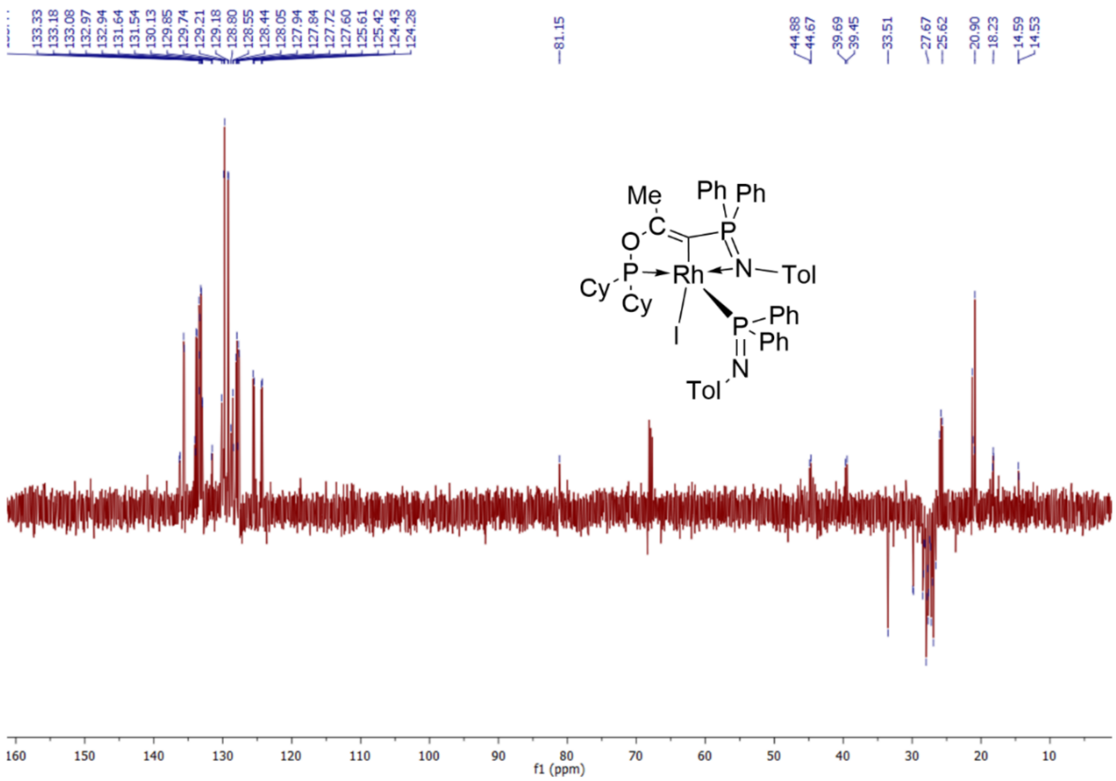

Figure S41. DEPT-135 NMR spectrum of complex **6** (101 MHz, THF-d8)

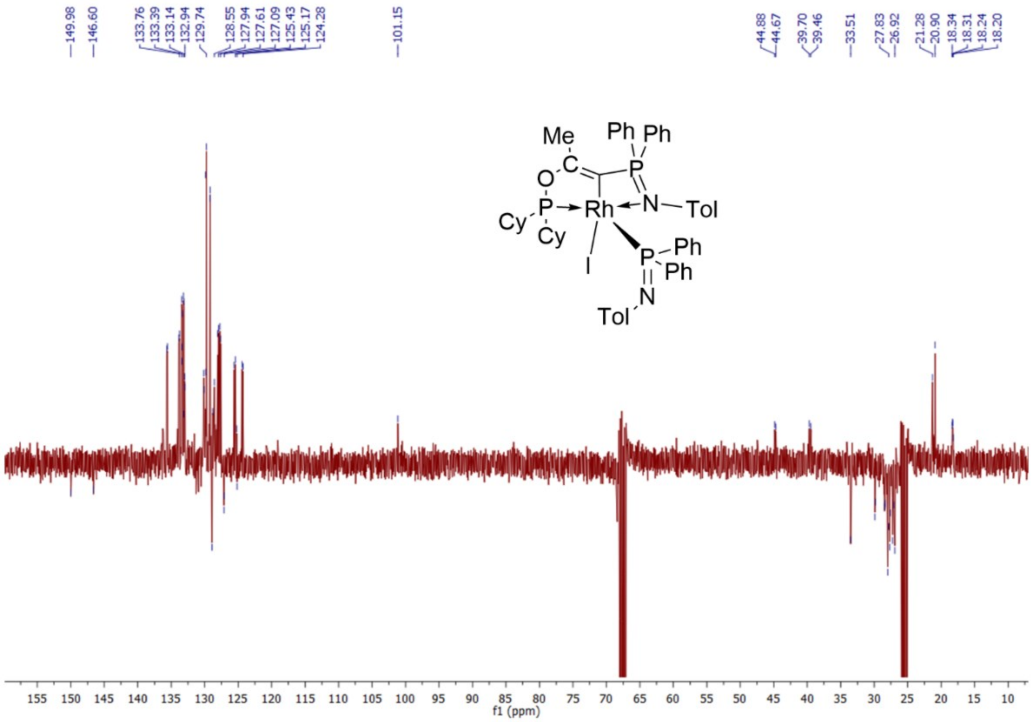

Figure S42. APT NMR spectrum of complex **6** (101 MHz, THF-d8). CH/CH_3 signals are positive, C/CH_2 signals are negative

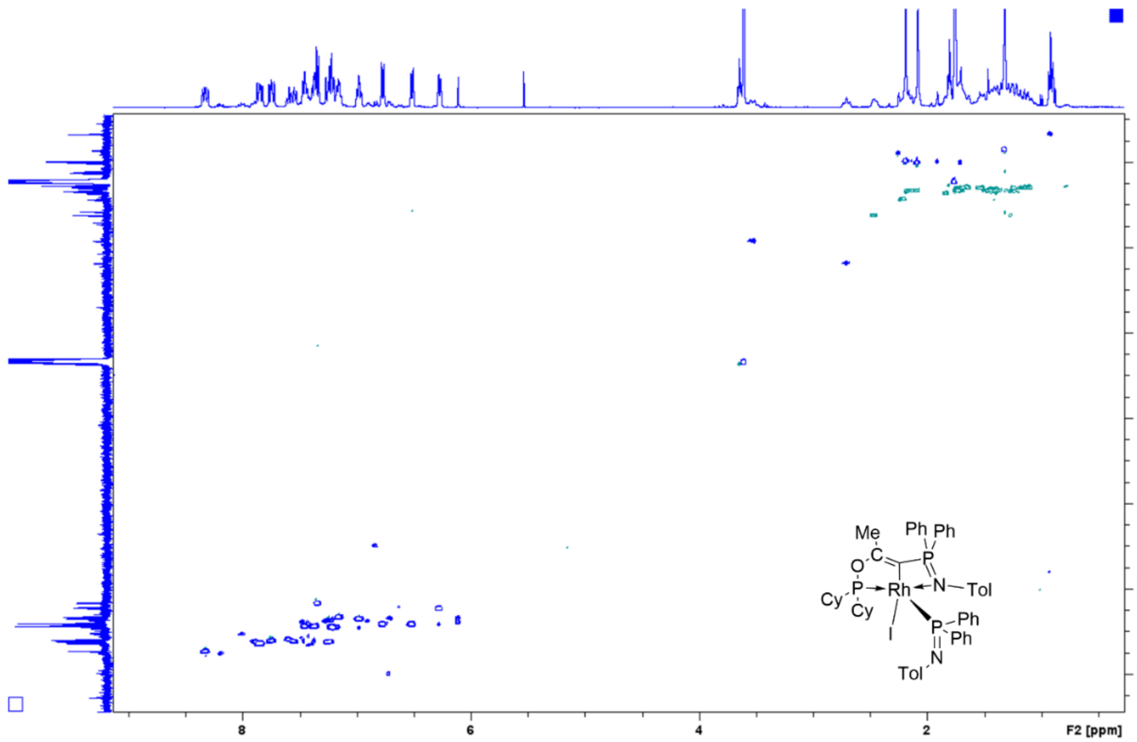

Figure S43. ^{13}C-^{1}H HSQC NMR spectrum of complex **6** (THF-d_8)

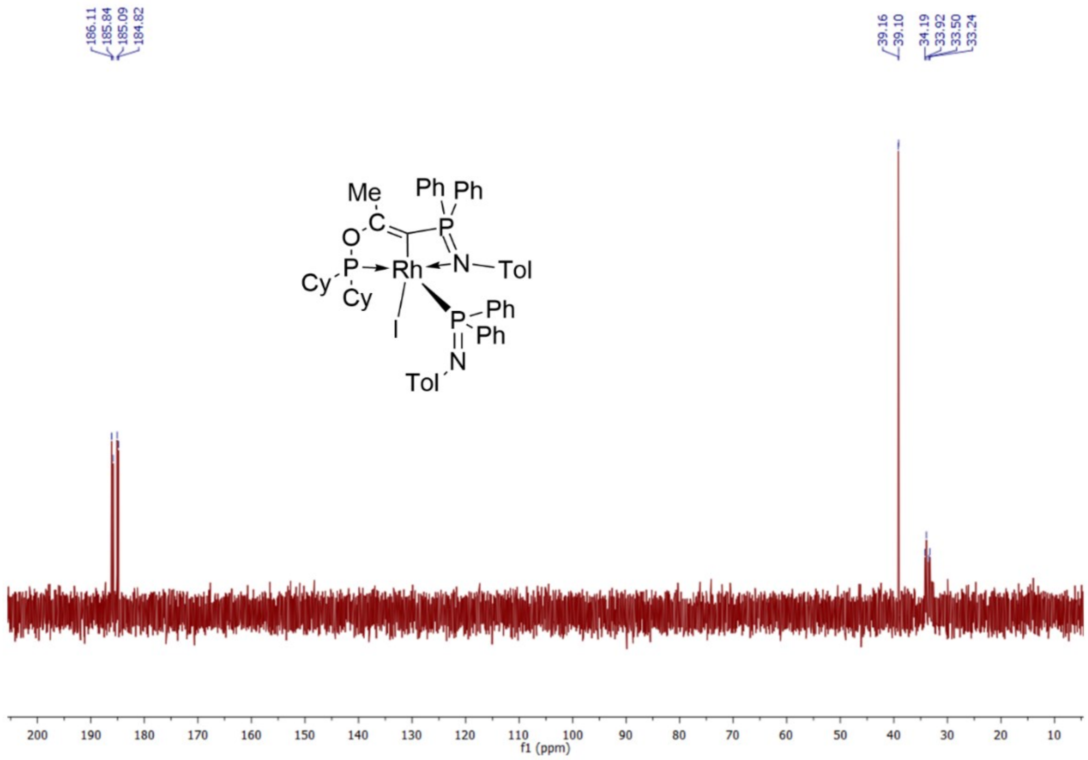

Figure S44. $^{31}P\{^{1}H\}$ NMR spectrum of complex **6** (162 MHz, THF-d_8)

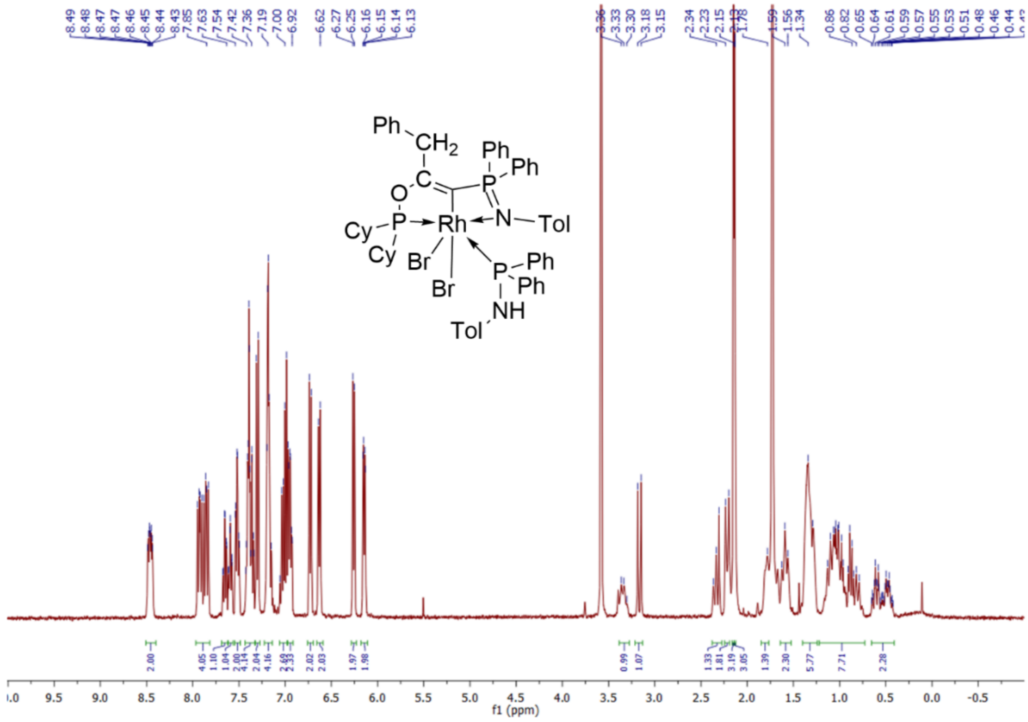

Figure S45. ^{1}H NMR spectrum of complex **8** (400 MHz, THF-d_8)

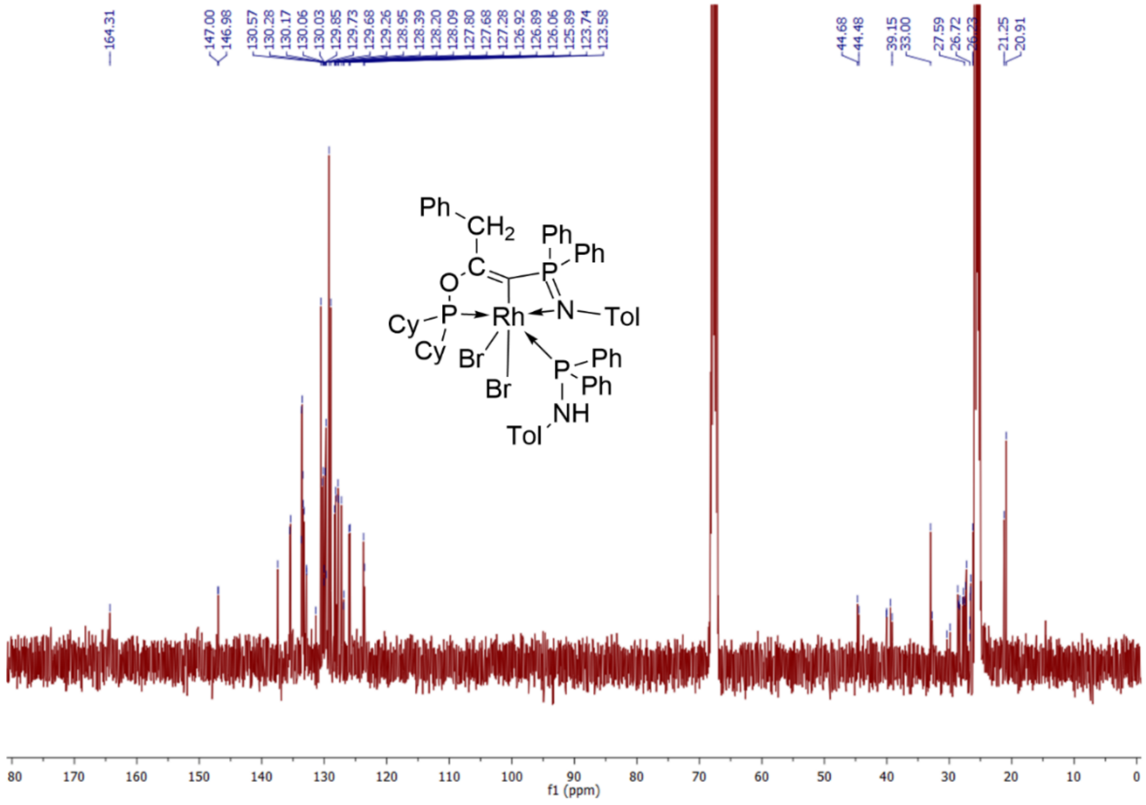

Figure S46. $^{13}C\{^{1}H\}$ NMR spectrum of complex **8** (101 MHz, THF-d_8)

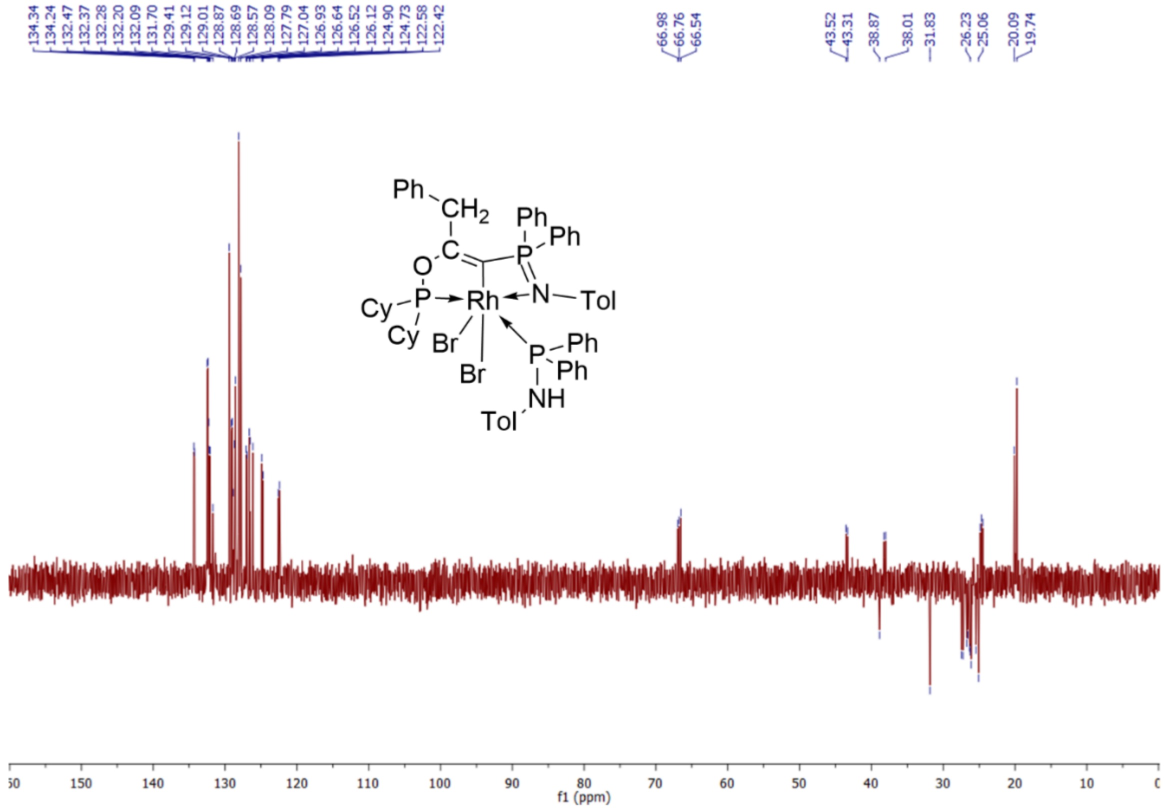

Figure S47. DEPT-135 NMR spectrum of complex **8** (101 MHz, THF-d_8)

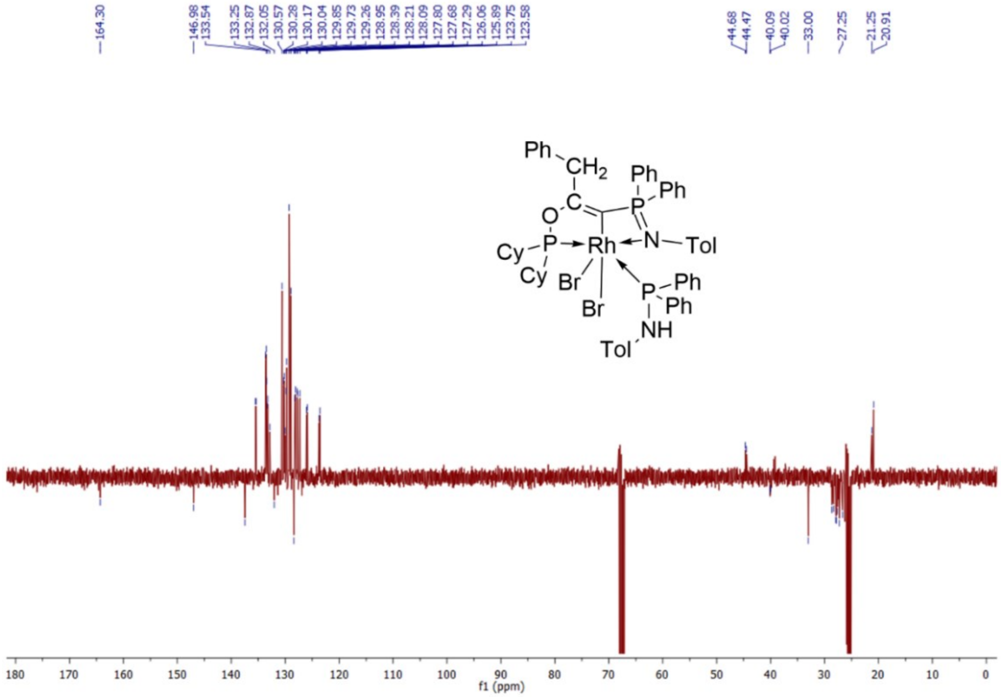

Figure S48. APT NMR spectrum of complex **8** (101 MHz, THF-d_8). CH/CH_3 signals are positive, C/CH_2 signals are negative.

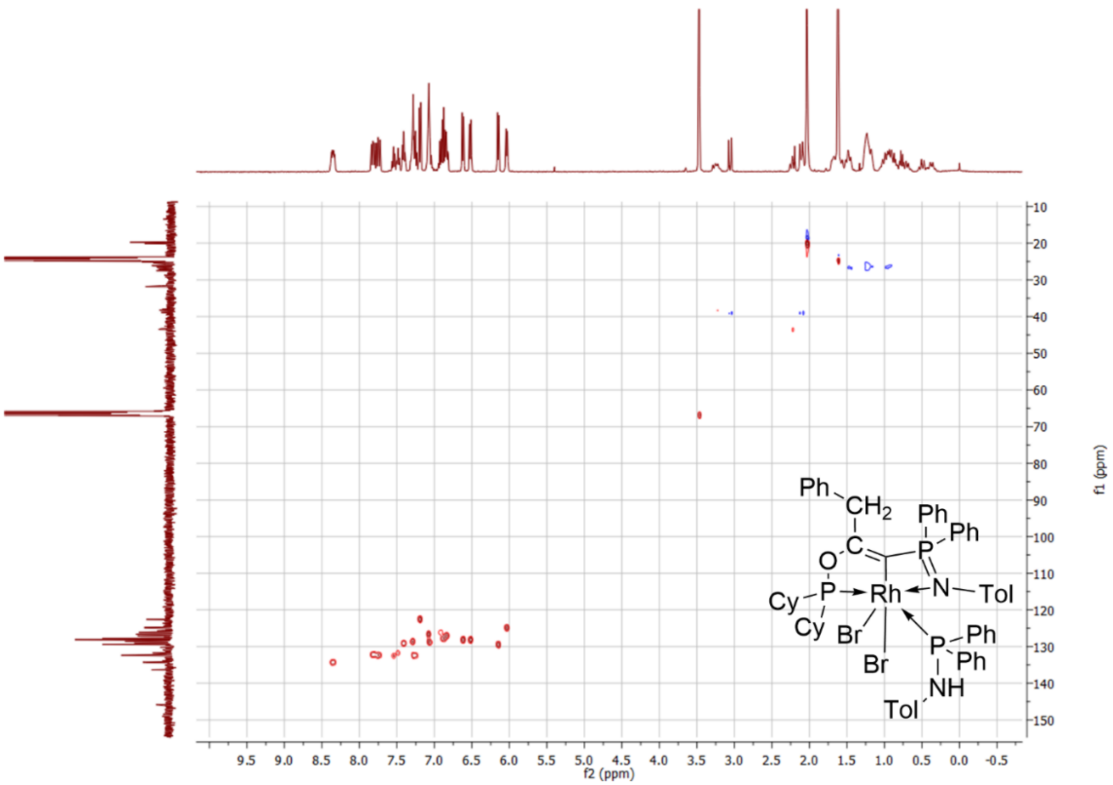

Figure S49. ^{13}C-^{1}H HSQC NMR spectrum of complex **8** (THF-d_8)

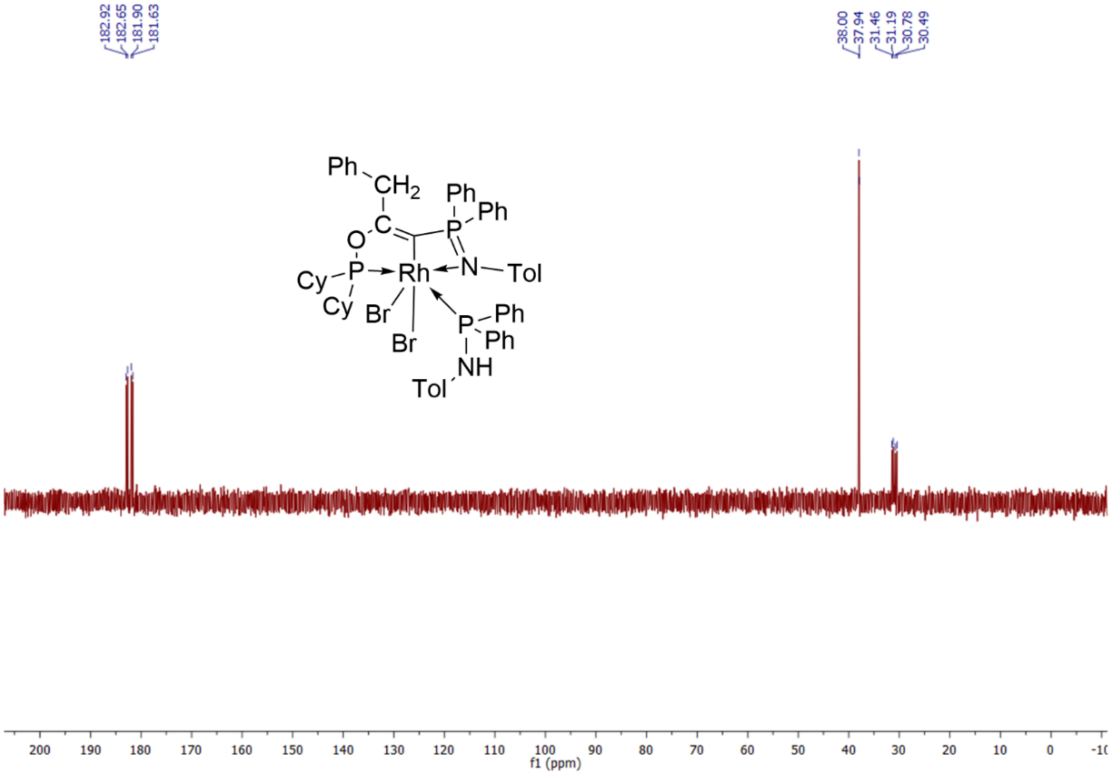

Figure S50. $^{31}P\{^{1}H\}$ NMR spectrum of complex **8** (162 MHz, THF-d_8)

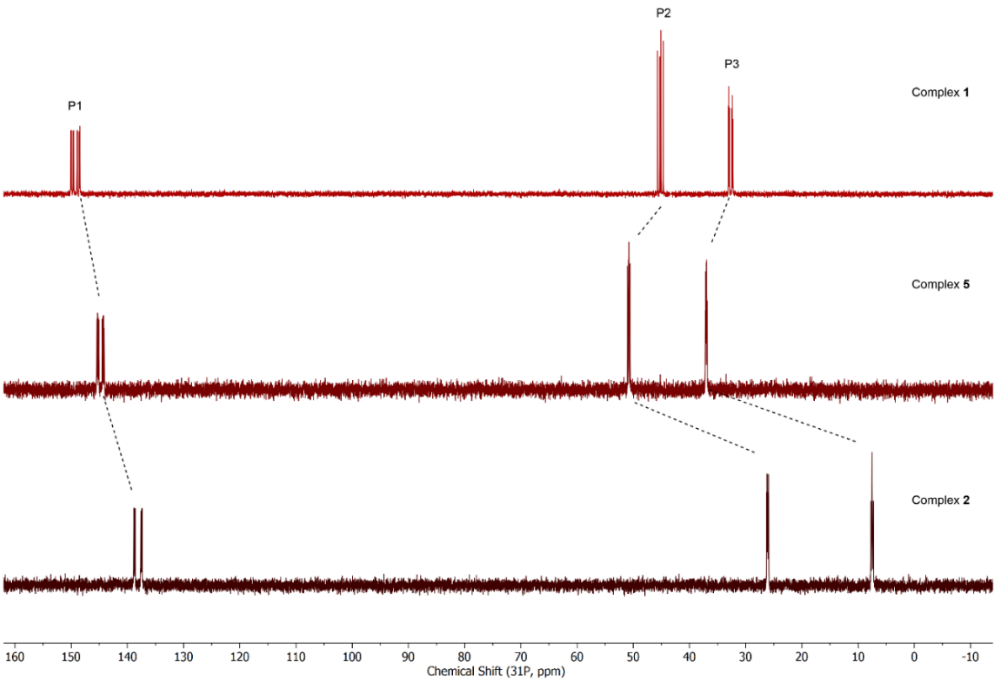

Figure S51. Comparison of the $^{31}P\{^{1}H\}$ NMR spectra of complexes **1**, **2**, and **5** (162 MHz, THF-d_8 (**1**, **2**), and $CDCl_3$ (**5**)). Top spectrum is complex **1** (red), middle is complex **5** (burgundy) and bottom is complex **2** (black).

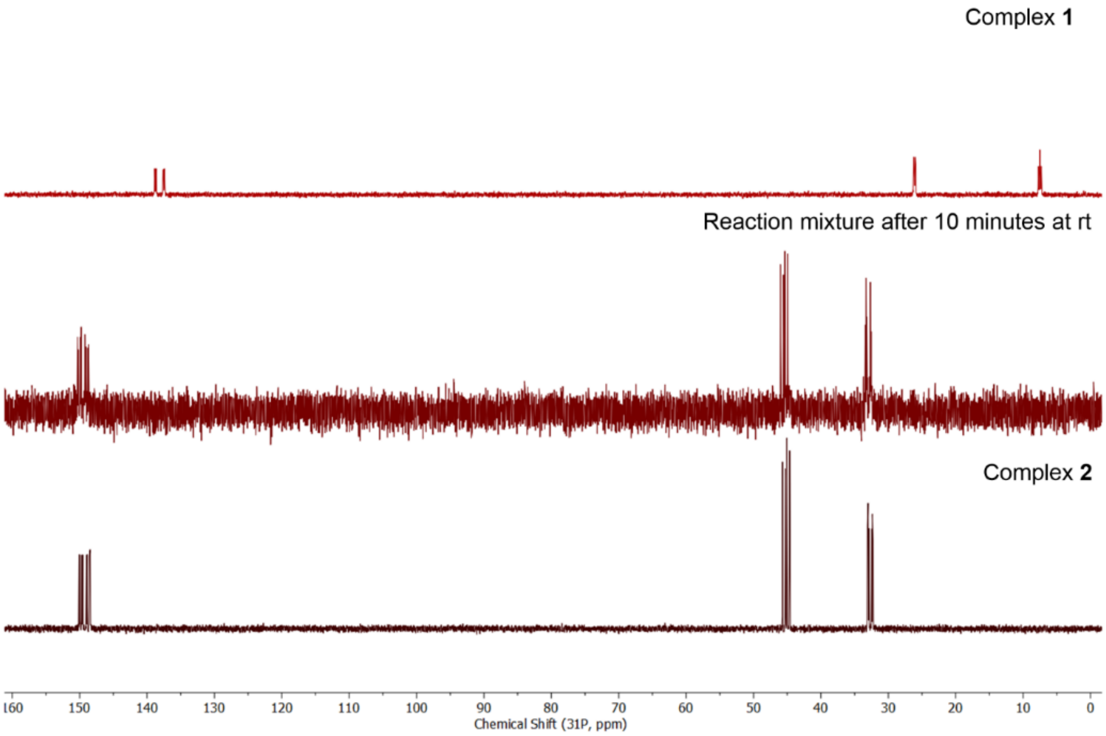

Figure S52. Stacked $^{31}P\{^{1}H\}$ NMR spectrum of the reaction between **1** and CO in the presence of 1.5 equivalents of NaHMDS to form complex **2** (162 MHz, THF-d_8).

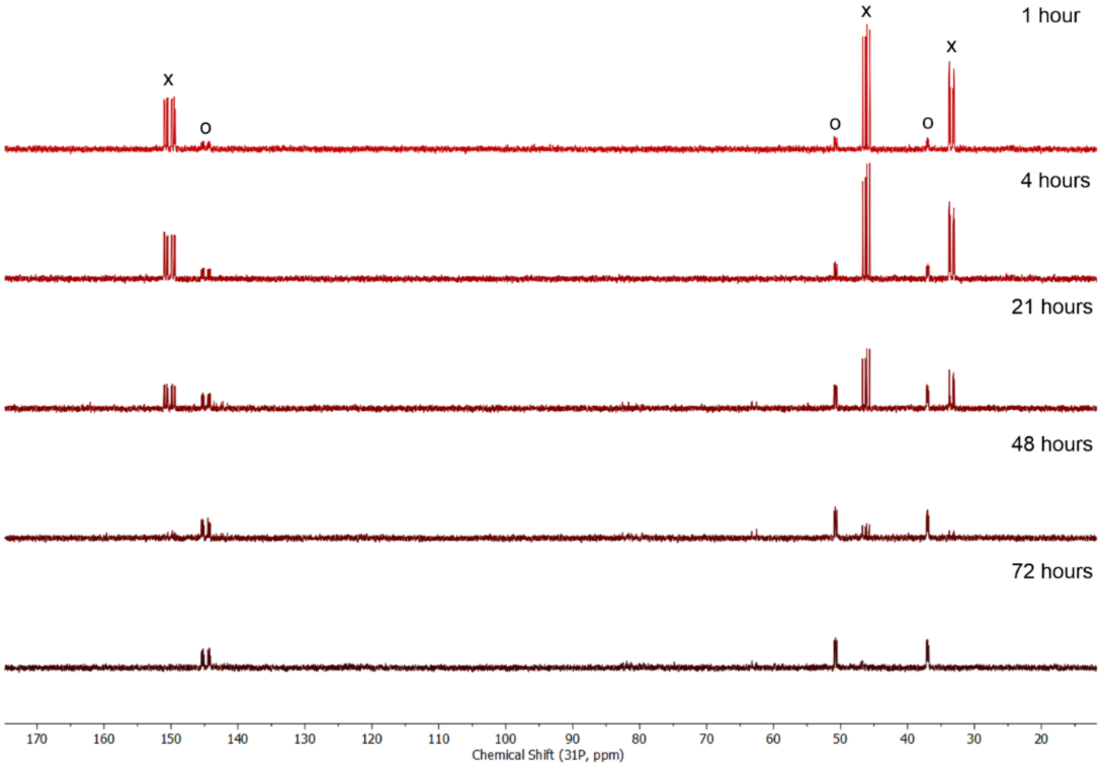

Figure S53. Stacked $^{31}P\{^{1}H\}$ NMR spectrum (162 MHz, $CDCl_3$) taken at different time of the reaction between **2** (denoted by an X) and 10 eq. isopropyl iodide over 72 hours at room temperature to form complex **5** (denoted by an O).

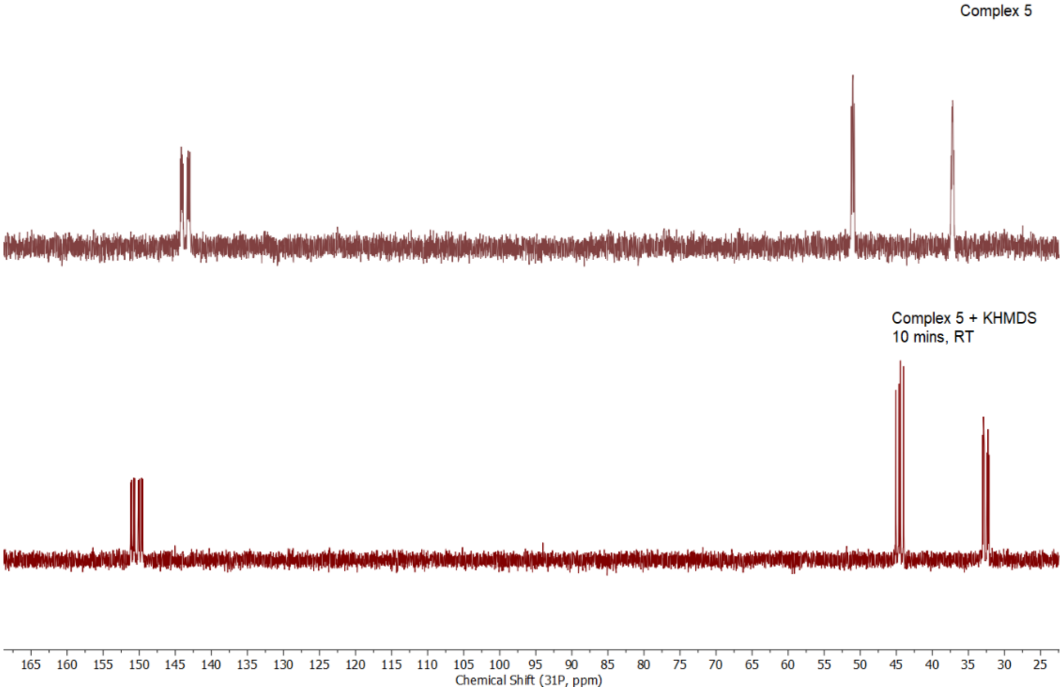

Figure S54. Stacked $^{31}P\{^{1}H\}$ NMR spectrum (162 MHz, C_6D_6) of complex **5** before addition of KHMDS (top) and 10 minutes at room temperature after addition of KHMDS (bottom).

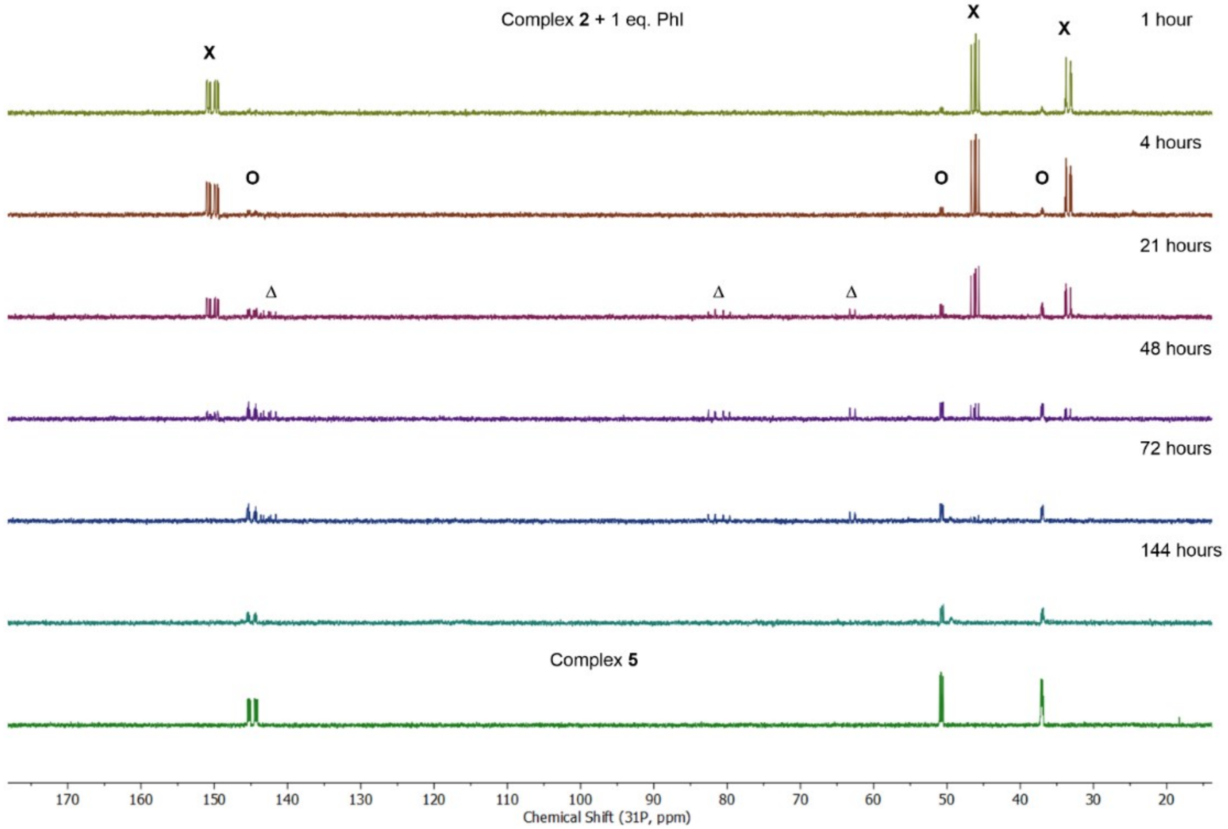

Figure S55. Stacked $^{31}P\{^{1}H\}$ NMR spectrum (162 MHz, $CDCl_3$) taken at different time of the reaction between **2** (denoted by an X) and 1 eq. iodobenzene over 144 hours at room temperature to form complex **5** (denoted by an O), and some unidentified product (denoted by a □).

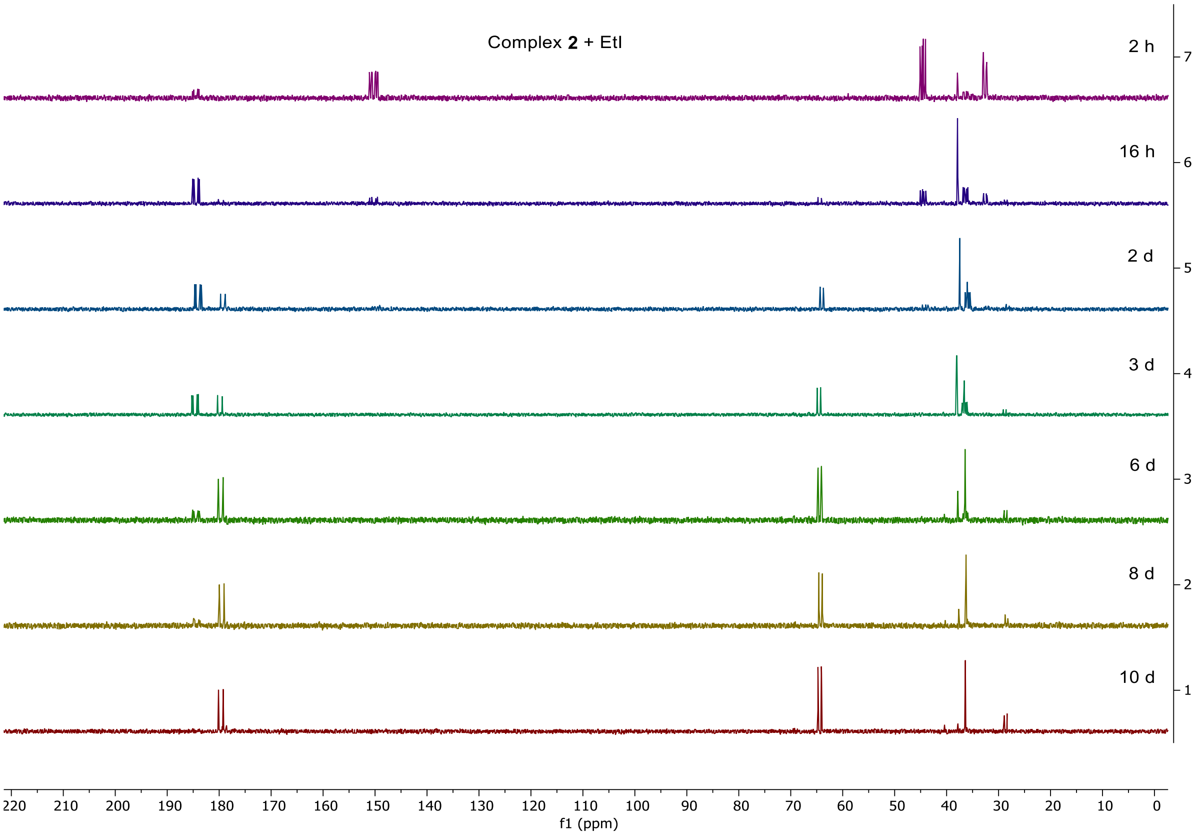

Figure S56. Stacked $^{31}P\{^{1}H\}$ NMR spectrum (162 MHz, $CDCl_3$) taken at different time of the reaction between complex **2** and EtI.

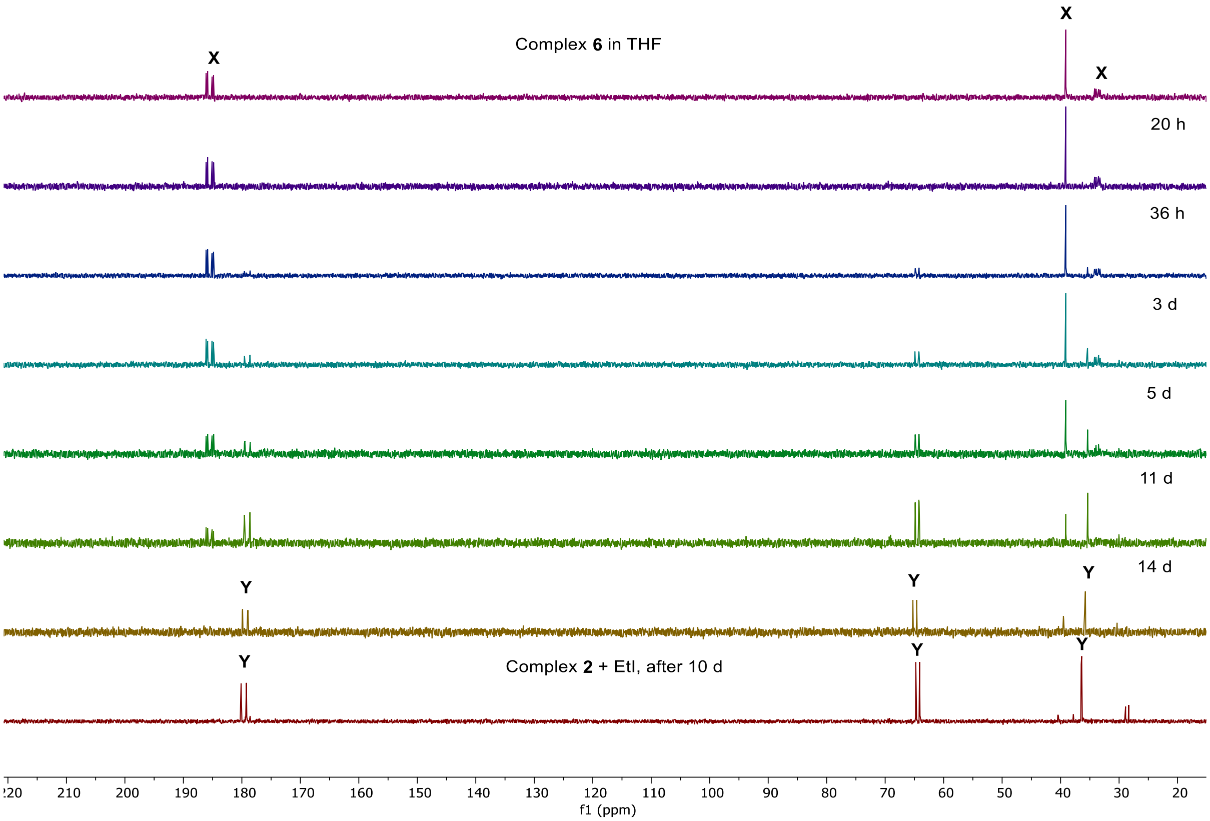

Figure S57. Stacked $^{31}P\{^{1}H\}$ NMR spectrum (162 MHz, $CDCl_3$) taken at different time of complex **6** in THF (denoted by **X**) which converts to the C-H activation product, complex **9** (denoted by Y) with time similar to the reaction between complex **2** and EtI after 10 d.

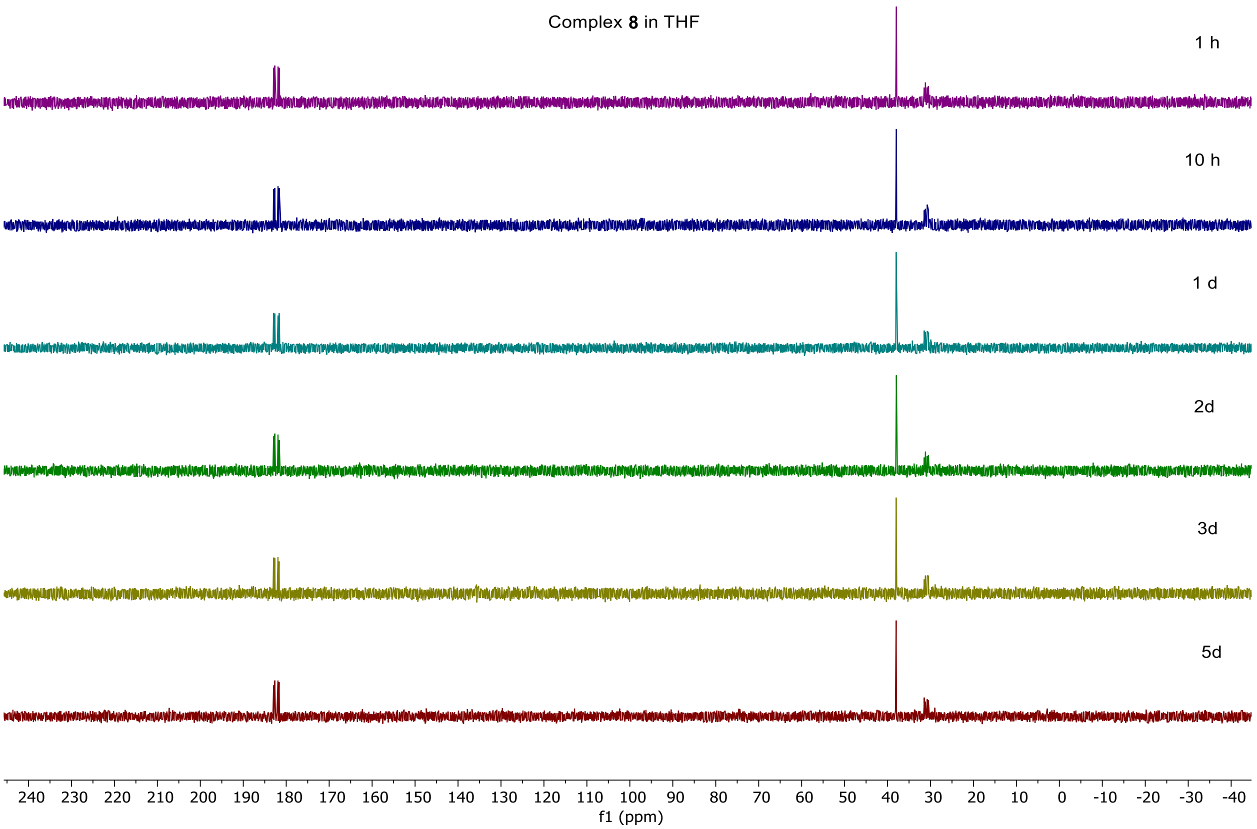

Figure S58. Stacked $^{31}P\{^{1}H\}$ NMR spectrum (162 MHz, $CDCl_3$) taken at different time of complex **8** in THF.

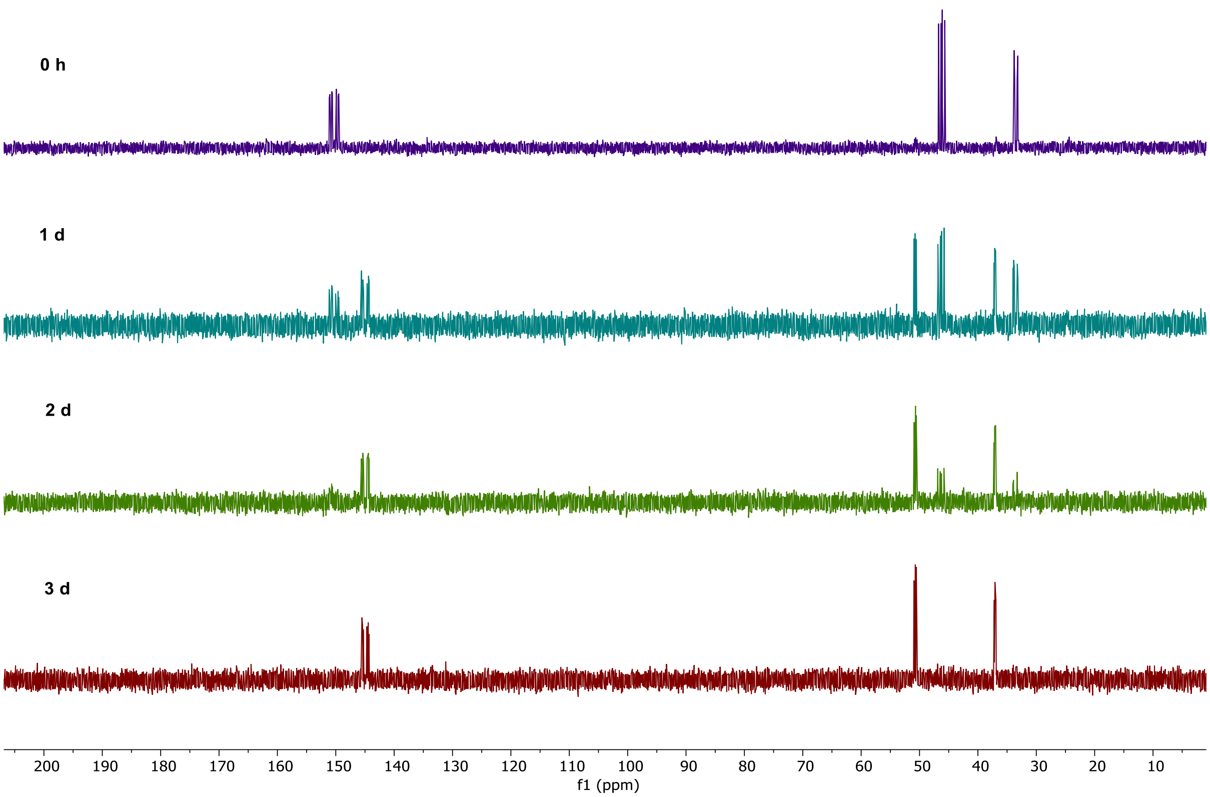

Figure S59. Stacked $^{31}P\{^{1}H\}$ NMR spectrum (162 MHz, $CHCl_3$) of the reaction of complex **2** with $CHCl_3$ taken at different times.

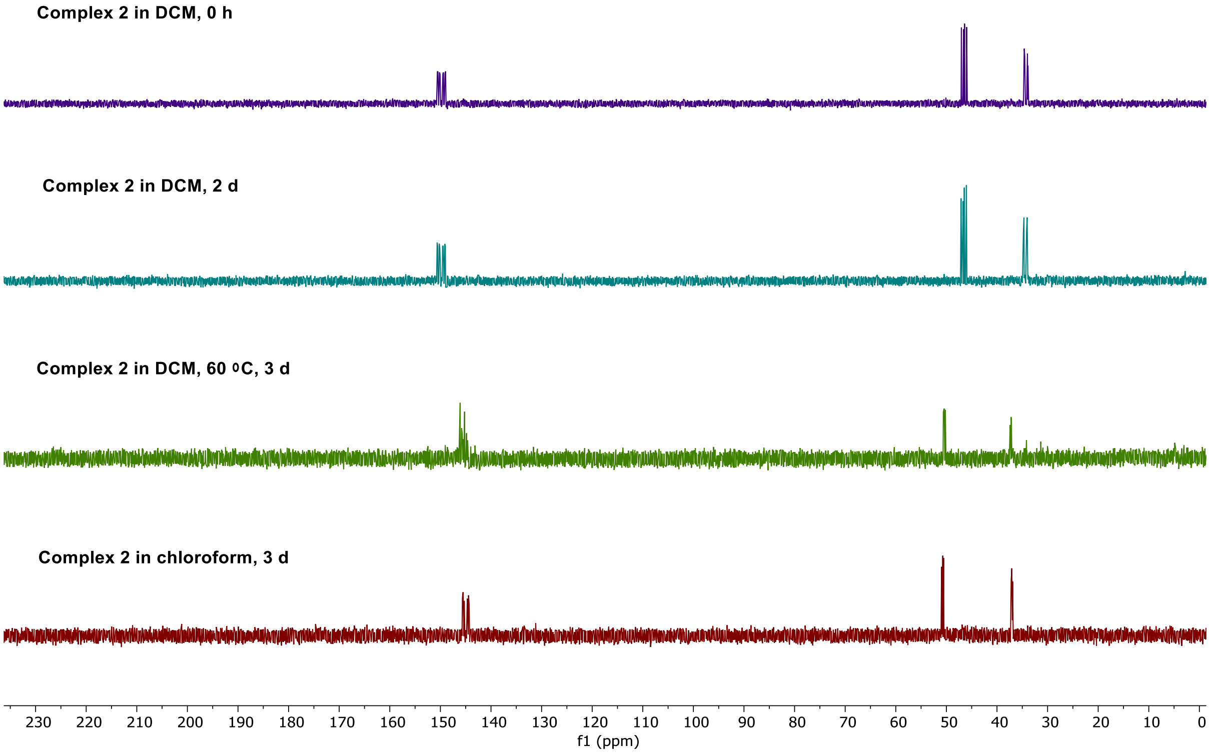

Figure S60. Stacked $^{31}P\{^{1}H\}$ NMR spectrum (162 MHz, CH_2Cl_2) of the reaction of complex **2** with CH_2Cl_2 taken at different time and temperatures.

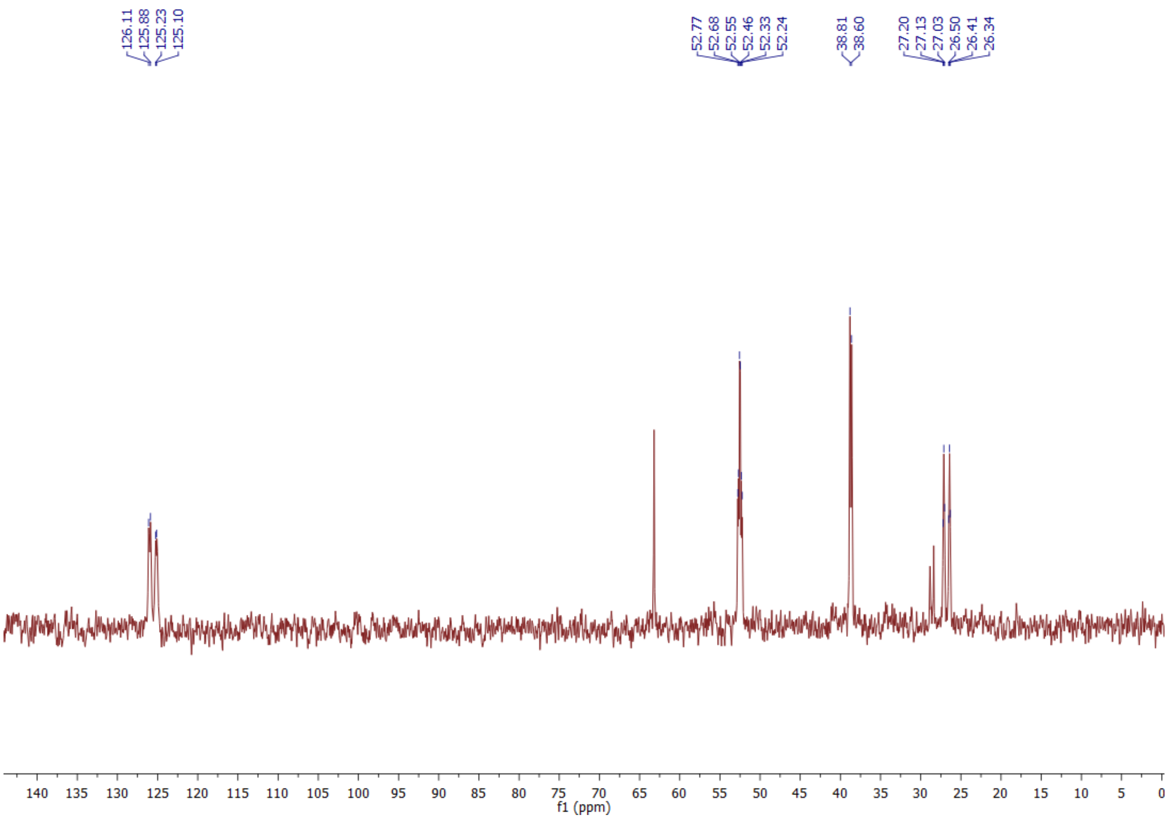

Figure S61. $^{31}P\{^{1}H\}$ NMR spectrum (162 MHz, THF-d_8) of the reaction between complex **3** and HCl (peak at 28 ppm correspond to Ph_3PO).

3. IR spectra

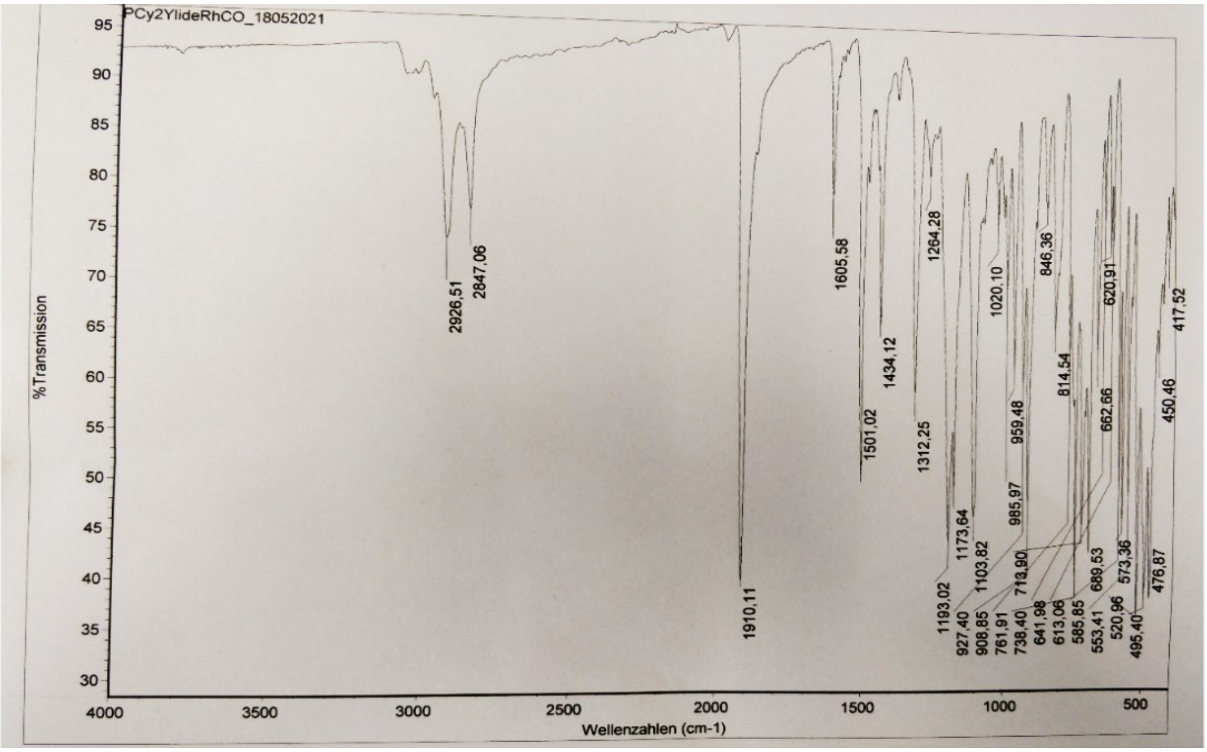

Figure S62. ATR-IR spectrum of complex **2**.

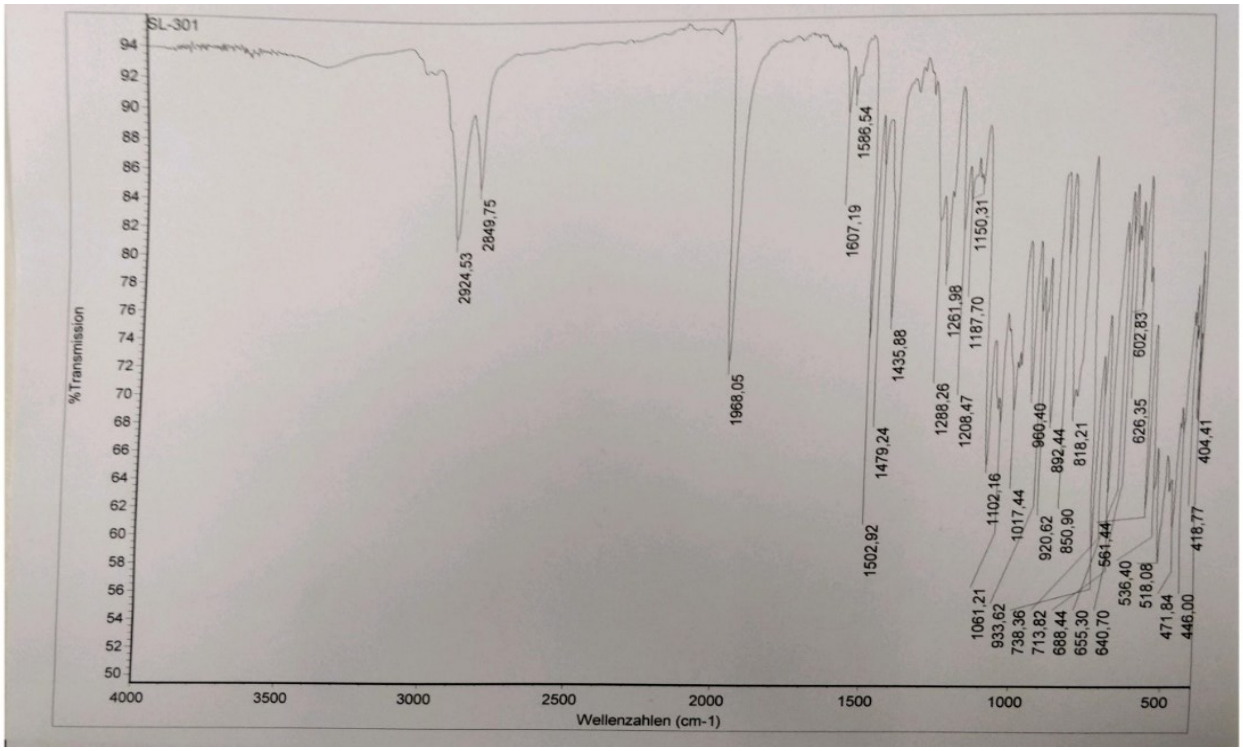

Figure S63. ATR-IR spectrum of complex **5**.

4. Crystal structure analyses

4.1. Crystallographic details

The X-ray diffraction data for the single crystals of **L1-Li**, **L2**, and **1-8** were collected on a Rigaku Synergy instrument (κ-goniometer) with a PILATUS3 R 200K hybrid pixel array detector using Mo*Kα* (0.71073 Å) or Cu*K*α (1.54184 Å) radiation monochromated by means of multilayer optics. The performance mode of a PhotonJet microfocus sealed X-ray tube was 50 kV, 1.0 mA. Suitable crystals of appropriate dimensions were mounted on loops in random orientations. Preliminary unit cell parameters were determined with three sets of a total of 10 narrow frame scans in the case of a Mo-source and six sets of a total of 10 narrow frame scans at two different 2θ positions in the case of a Cu-source. The data were collected according to recommended strategies in an ω-scan mode. Final cell constants were determined by global refinement of reflections from the complete data sets using the Lattice wizard module. Images were indexed and integrated with "smart" background evaluation using the *CrysAlisPro* data reduction package (Rigaku Oxford Diffraction). Analysis of the integrated data did not show any decay. Data were corrected for systematic errors and absorption using the ABSPACK module: Numerical absorption correction based on Gaussian integration over a multifaceted crystal model and empirical absorption correction based on spherical harmonics according to the point group symmetry using equivalent reflections.

The structures were solved by intrinsic phasing using *SHELXT*-2018/2 and refined by the full-matrix least-squares on F^2 using *SHELXL*-2018/3,[3-4] which uses a model of atomic scattering based on spherical atoms. Non-hydrogen atoms were refined anisotropically. The positions of the hydrogen atoms at the carbon bound to the rhodium atoms were determined by the difference Fourier maps, and these atoms were refined isotropically. The other hydrogen atoms were inserted at the calculated positions and refined as riding atoms.

Crystallographic data including structure factors have been deposited with the Cambridge Crystallographic Data Centre as supplementary publication no. CCDC-2220827-2220835 (all ligands structures and complexes **1-8**) and CCDC-2224054 for complex **9**. Copies of the data can be gained free of charge on application to Cambridge Crystallographic Data Centre, 12 Union Road, Cambridge CB2 1EZ, UK; [fax: (+44) 1223-336-033; email: deposit@ccdc.cam.ac.uk]. An *ORTEP III*[5] view of all compounds were drawn with 50% probability displacement ellipsoids and H atoms omitted for clarity.

Table S1. Crystal data collection and refinement parameters for **L1-Li, L2** and **1**.

	**L1-Li**	**L2**	**1**
CCDC number	2220833	2220827	2220835
Empirical formula	$C_{43}H_{43}LiN_2OP_2$	$C_{51}H_{57}N_2P_3$	$C_{55}H_{65}N_2CLOP_3$ Rh
crystal colour	colourless	orange	orange
Formula weight (g/mol)	672.67	790.89	1001.36
F(000)	1424	844	1048
T (K)	100(2)	100(2)	100(2)
wavelength (Å)	1.54184	1.54184	0.71073
space group	*P*-1	*P*2$_1$	*P*-1
a (Å)	11.62871(11)	9.72400(10)	11.69260(10)
b (Å)	14.70630(14)	22.58670(10)	14.4062(2)
c (Å)	22.76166(16)	10.30020(10)	16.2053(2)
α (deg)	81.2531(7)	90	105.0020(10)
β (deg)	77.8491(7)	106.2070(10)	95.7050(10)
γ (deg)	73.0988(8)	90	98.0360(10)
Z	4	2	2
V (Å^3)	3623.80(6)	2172.36(3)	2584.42(5)
ρ_{calcd} (g·cm^{-3})	1.233	1.209	1.287
μ (mm^{-1})	1.359	1.529	0.514
θ range (deg)	3.156 − 77.746	3.914 − 77.700	2.240 − 30.588
Completeness (%)	100	100	99.9
collected reflections; R_σ	115730; 0.0237	43386; 0.0370	77778; 0.0279
independent reflections; R_{int}	15176; 0.0581	8909; 0.0610	14248; 0.0365
Data / restraints / parameters	15176 / 0 / 887	8909 / 1 / 511	14248 / 6 / 675
R1^a; wR2^b [I > 2σ(I)]	0.0444; 0.1180	0.0343; 0.0878	0.0319; 0.0798
R1; wR2 [all data]	0.0475; 0.1206	0.0354; 0.0889	0.0421; 0.0845
GOF	1.027	1.047	1.035
largest diff peak and hole	1.057 and -0.468	0.332 and -0.308	2.593 and -0.709

^{a} $R_1=\Sigma(||F_o|-|F_c||)/\Sigma|F_o|$; ^{b} $wR_2=\{\Sigma[w(F_o^2–F_c^2)^2]/\Sigma[w(F_o^2)^2]\}^{½}$

Table S2. Crystal data collection and refinement parameters for complexes **2**, **4** and **5**.

	**2**	**4**	**5**
CCDC number	2220834	2220829	2220832
Empirical formula	$C_{108}H_{120}N_4O_3P_6Rh_2$	$C_{54}H_{65}N_2P_4Rh$	$2\cdot(C_{52}H_{57}N_2OP_3Rh, I)$+1/toluene
crystal colour	orange	dark red	yellow
Formula weight (g/mol)	1913.71	968.87	2143.49
T (K)	100(2)	100(2)	100(2)
wavelength (Å)	1.54184	0.71073	1.54184
space group	*P*-1	$P2_1$	*P*-1
a (Å)	13.4359(2)	12.2238(2)	17.98950(10)
b (Å)	14.5922(2)	33.5168(4)	19.07560(10)
c (Å)	28.2841(4)	12.31380(10)	19.21910(10)
α (deg)	83.8050(10)	90	89.7830(10)
β (deg)	82.1580(10)	105.0650(10)	67.2340(10)
γ (deg)	65.890(2)	90	62.2950(10)
Z	2	4	2
V ($Å^3$)	5005.91(14)	4871.61(11)	5257.42(7)
ρ_{calcd} ($g\cdot cm^{-3}$)	1.270	1.321	1.354
μ (mm^{-1})	3.966	0.520	8.381
F(000)	2000	2032	2186
θ range (deg)	3.160 − 77.283	2.419 − 33.174	2.553 − 62.498
Completeness (%)	99.9	99.9	100
collected reflections; R_σ	148956; 0.0274	117672; 0.0340	149708; 0.0249
independent reflections; R_{int}	20689; 0.0710	32348; 0.0396	16758; 0.0639
Data / restraints / parameters	20689 / 24 / 1177	32348 / 1 / 1109	16758 / 30 / 1128
$R1^a$; $wR2^b$ [I > 2σ(I)]	0.0443; 0.1194	0.0320; 0.0762	0.0367; 0.0889
R1; wR2 [all data]	0.0468; 0.1216	0.0358; 0.0778	0.0381; 0.0899
GOF	1.023	1.0623	1.037
largest diff peak and hole	1.016 and -1.678	0.694 and -0.525	1.482 and -0.999

[a] $R_1=\Sigma(||F_o|-|F_c||)/\Sigma|F_o|$; [b] $wR_2=\{\Sigma[w(F_o^2–F_c^2)^2]/\Sigma[w(F_o^2)^2]\}^{½}$

Table S3. Crystal data collection and refinement parameters for complexes **6, 7** and **8**.

	**6**	**7**	**8**
CCDC number	2220828	2220831	2220830
Empirical formula	$C_{53}H_{59}IN_2OP_3Rh$	$C_{54}H_{61}IN_2OP_3Rh$	$C_{59}H_{64}Br_2N_2OP_3Rh$
crystal colour	orange	black	orange
Formula weight (g/mol)	1062.74	1076.76	1172.76
T (K)	100(2)	99.9(4)	100(2)
wavelength (Å)	1.54184	1.54184	1.54184
space group	*P*-1	$P2_1$/c	*P*-1
a (Å)	11.5146(2)	11.42870(10)	10.7834(2)
b (Å)	13.8208(2)	25.4664(3)	14.9584(3)
c (Å)	16.3726(3)	39.3056(7)	19.8288(3)
α (deg)	76.3500(10)	90	96.372(2)
β (deg)	86.7050(10)	110.858(2)	103.5600(10)
γ (deg)	79.9730(10)	90	100.505(2)
Z	2	8	2
V ($Å^3$)	2492.92(7)	10690.1(3)	3017.36(10)
ρ_{calcd} ($g\cdot cm^{-3}$)	1.416	1.338	1.291
μ (mm^{-1})	8.832	8.246	4.874
F(000)	1084	4400	1200
θ range (deg)	2.778 − 76.778	2.967 − 76.986	2.322 − 76.832
Completeness (%)	99.9	99.9	99.7
collected reflections; R_σ	75040; 0.0234	144407; 0.0407	81811; 0.0475
independent reflections; R_{int}	10165; 0.0457	21747; 0.0762	12212; 0.1193
Data / restraints / parameters	10165 / 0 / 553	21747 / 0 / 1123	12212 / 0 / 668
R1[a]; wR2[b] [I > 2σ(I)]	0.0235; 0.0606	0.0546; 0.1455	0.0776; 0.2247
R1; wR2 [all data]	0.0254; 0.0615	0.0711; 0.1559	0.0862; 0.2356
GOF	1.089	1.065	1.082
largest diff peak and hole	0.558 and -0.676	1.031 and -1.603	1.996 and -1.824

[a] $R_1=\Sigma(||F_o|-|F_c||)/\Sigma|F_o|$; [b] $wR_2=\{\Sigma[w(F_o^2-F_c^2)^2]/\Sigma[w(F_o^2)^2]\}^{1/2}$

Table S4. Crystal data collection and refinement parameters for complex **9**

	**9**
CCDC number	2224054
Empirical formula	$C_{53}H_{59}IN_2OP_3Rh$
crystal colour	Clear yellow
Formula weight (g/mol)	1062.74; 9756
T (K)	100(2)
wavelength (Å)	1.54184
space group	R -3
a (Å)	43.4288(5)
b (Å)	43.4288(5)
c (Å)	13.10301(16)
α (deg)	90
β (deg)	90
γ (deg)	120
Z	18
V (Å^3)	21402.2(6)
ρ_{calcd} (g·cm^{-3})	1.484
μ (mm^{-1})	9.259
F(000)	9756
θ range (deg)	3.525 − 68.215
Completeness (%)	100
collected reflections; R_σ	68652; 0.0274
independent reflections; R_{int}	68652; 0.0680
Data / restraints / parameters	8700 / 367 / 631
R1[a]; wR2[b] [I > 2σ(I)]	0.0448; 0.1119
R1; wR2 [all data]	0.0491; 0.1145
GOF	1.040
largest diff peak and hole	0.999 and -1.186

[a] $R_1=\Sigma(||F_o|-|F_c||)/\Sigma|F_o|$; [b] $wR_2=\{\Sigma[w(F_o^2–F_c^2)^2]/\Sigma[w(F_o^2)^2]\}^{½}$

4.2. Crystal structure analysis of 1-Li

H1 was found in the difference Fourier map and refined independently.

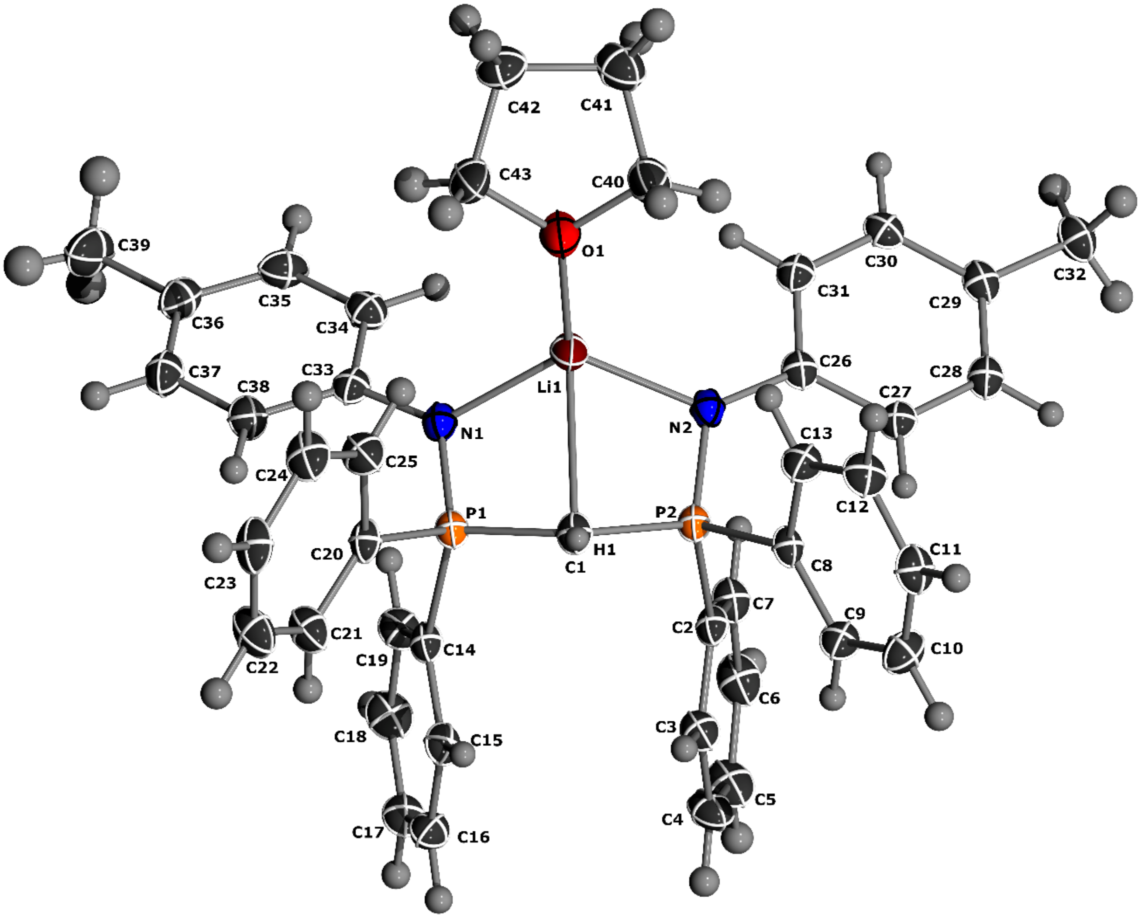

Figure S64 Structural representation of ligand **L1-Li** showing 50 % probability anisotropic displacement ellipsoids of non-hydrogen atoms.

4.3. Crystal structure analysis of L2

H1 was found in the difference Fourier map and refined independently.

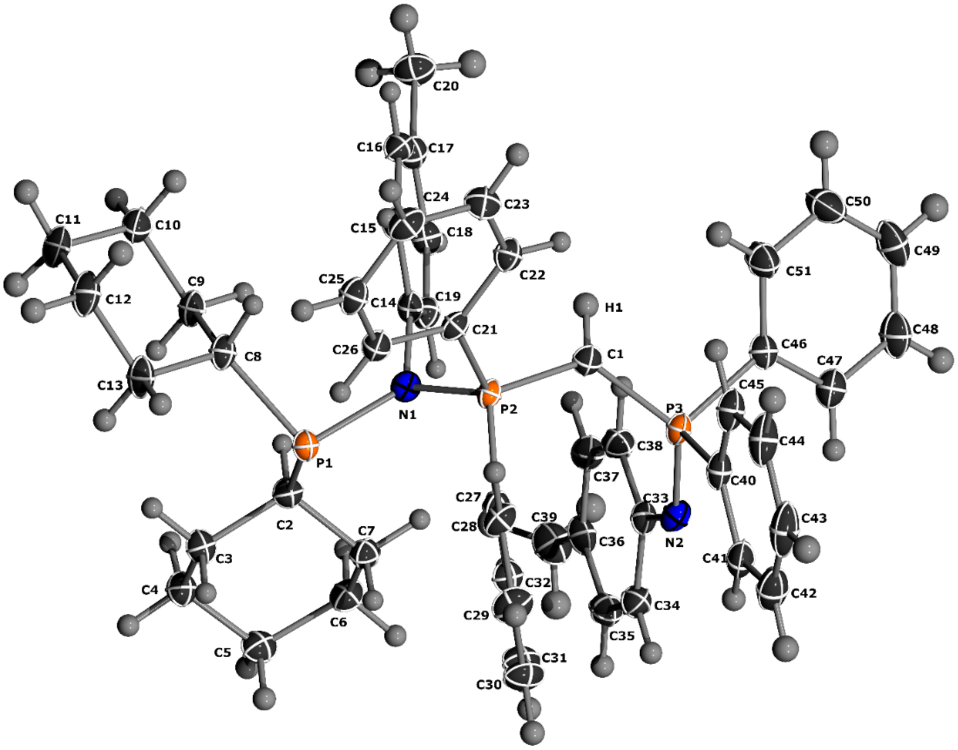

Figure S65. Structural representation of ligand **L2** showing 50 % probability anisotropic displacement ellipsoids of non-hydrogen atoms.

4.4. Crystal structure analysis of complex 1

The crystal structure contains a disordered cyclohexyl group and a disordered THF solvent molecule. The two parts of both disorders were refined using the PART keyword and free variables which refined to 0.795 (cyclohexyl) and 0.734 (THF), respectively. Furthermore, one carbon atom in the disordered THF molecule was refined using the ISOR restraint (0.01).

H1 was found in the difference Fourier map and refined independently.

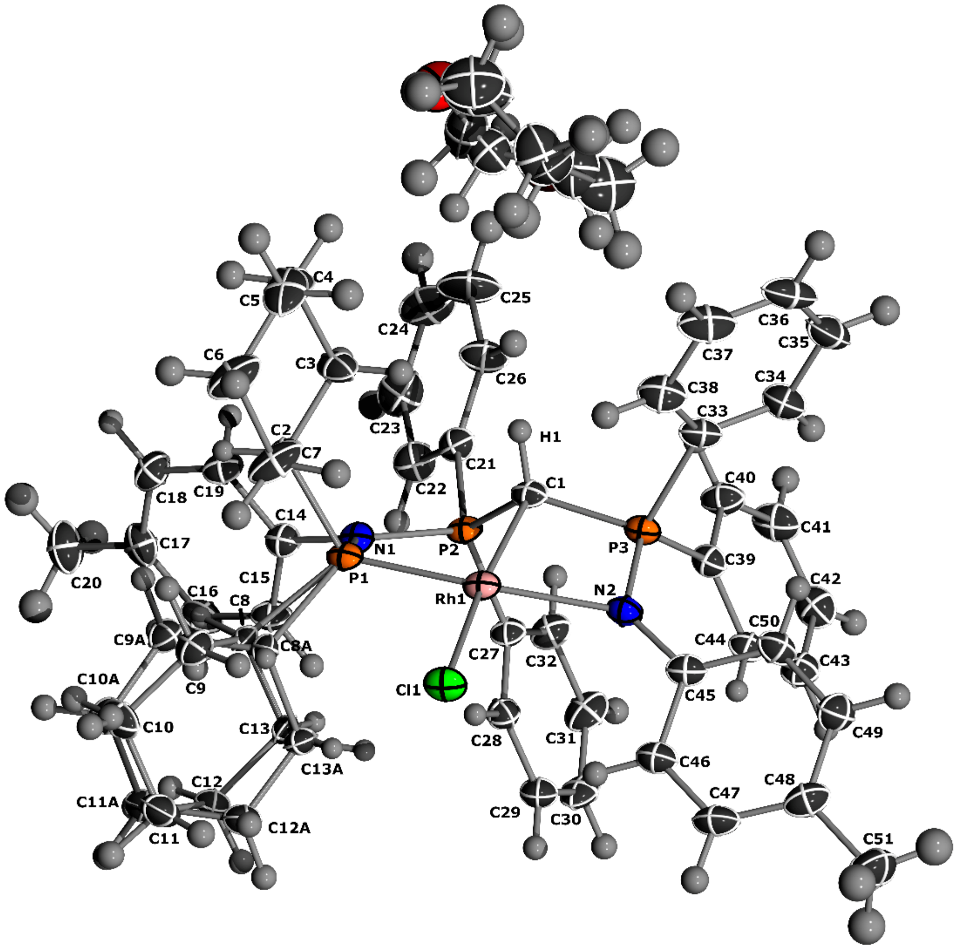

Figure S66. Structural representation of complex **1** with 50 % probability anisotropic displacement ellipsoids of non-hydrogen atoms. Hydrogen atoms, except for H1, as well as solvent molecules have been omitted for clarity. Only the major disordered moiety is shown.

4.5. Crystal structure analysis of complex 2

The crystal structure contains a disordered tolyl group, which was refined using the PART keyword and a free variable which refined to 0.451. SADI and FLAT restraints were used for the refinement of both parts.

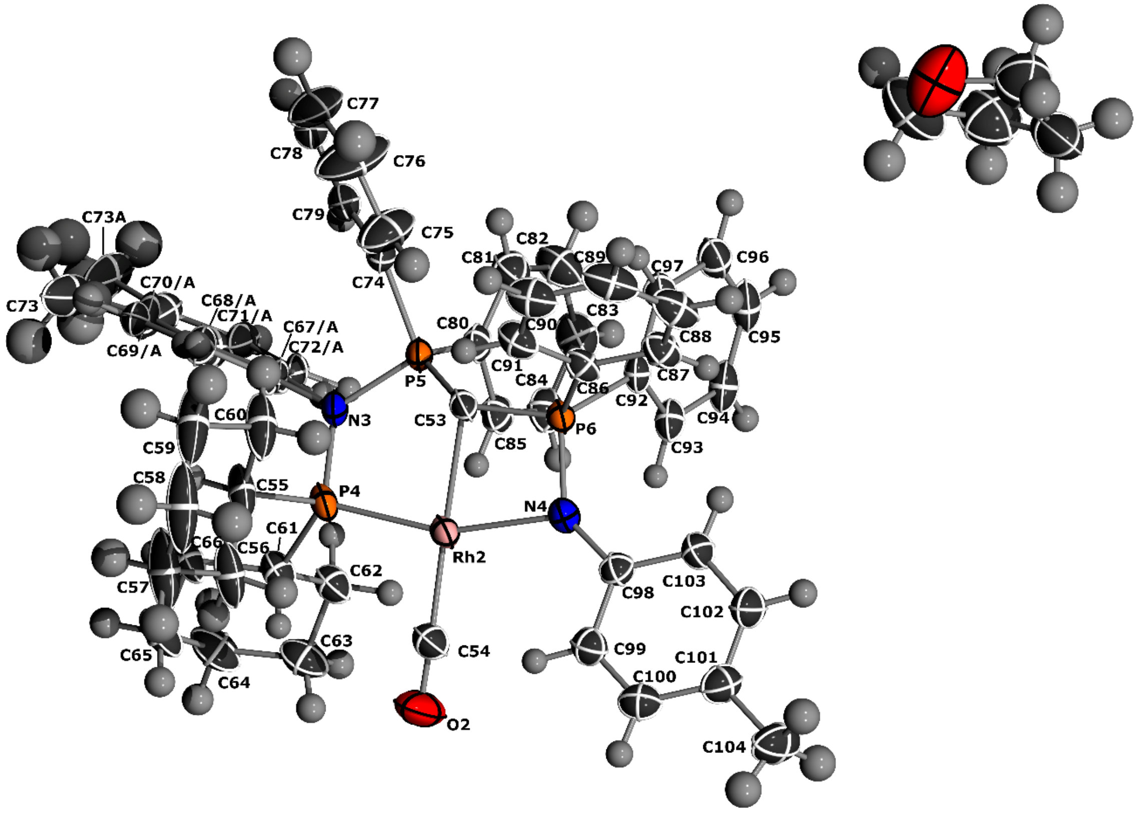

Figure S67. Structural representation of complex **2** showing 50 % probability anisotropic displacement ellipsoids of non-hydrogen atoms. Only one of the two complexes in the asymmetric unit is shown.

4.6. Crystal structure analysis of complex 4

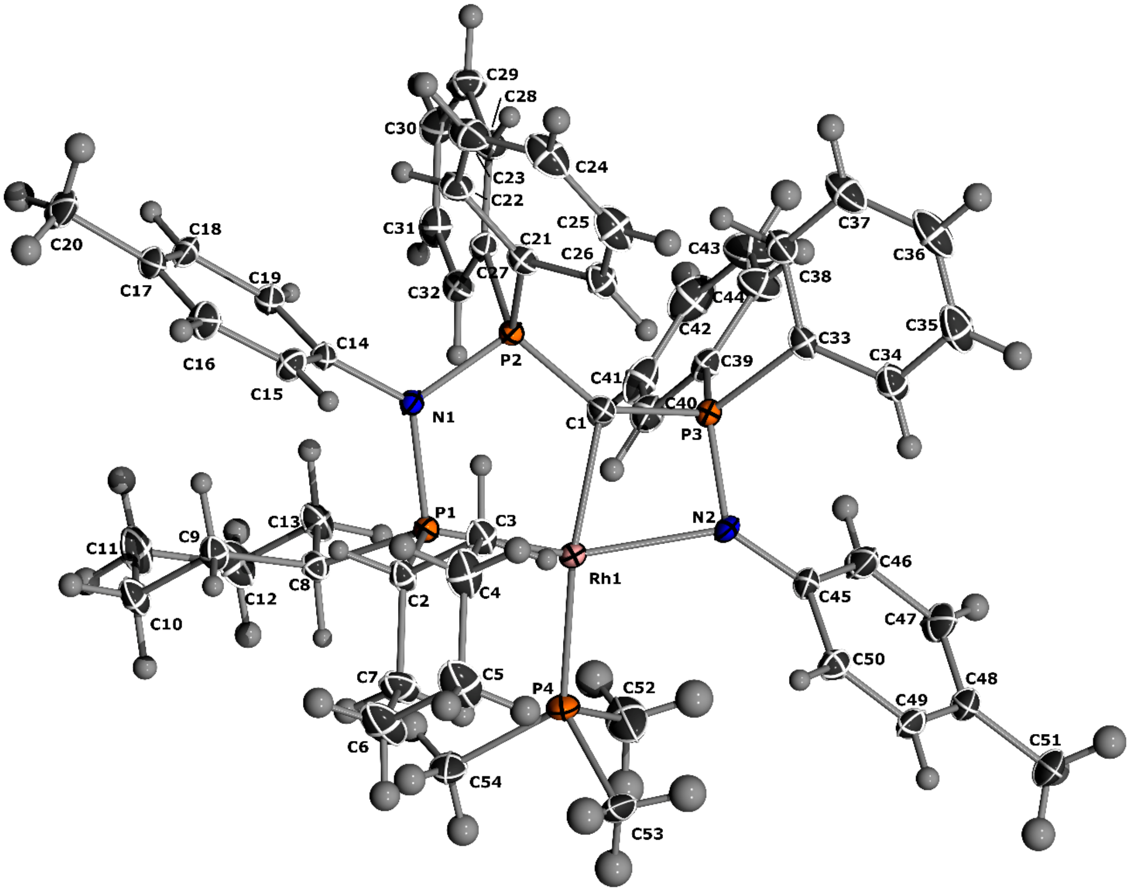

Figure S68. ORTEP diagram showing 50 % probability anisotropic displacement ellipsoids of non-hydrogen atoms for complex **4**. Two molecules are present in the asymmetric unit.

4.7. Crystal structure analysis of complex 5

The asymmetric unit contains two molecules of the Rh complex as well as a toluene solvent molecule on an inversion center. An additional heavily disordered solvent molecule was present in the structure, but couldn't be refined. Therefore, the SQUEEZE option in platon was used to minimize the void connected with the solvent. The toluene solvent on the symmetry centre was refined using the PART -1 keyword and fitted to an ideal solvent using the FRAG option. In the refinement process ISOR and SIMU as well as EADP restraints were used.

H1 and H53 were found in the difference Fourier map and refined independently.

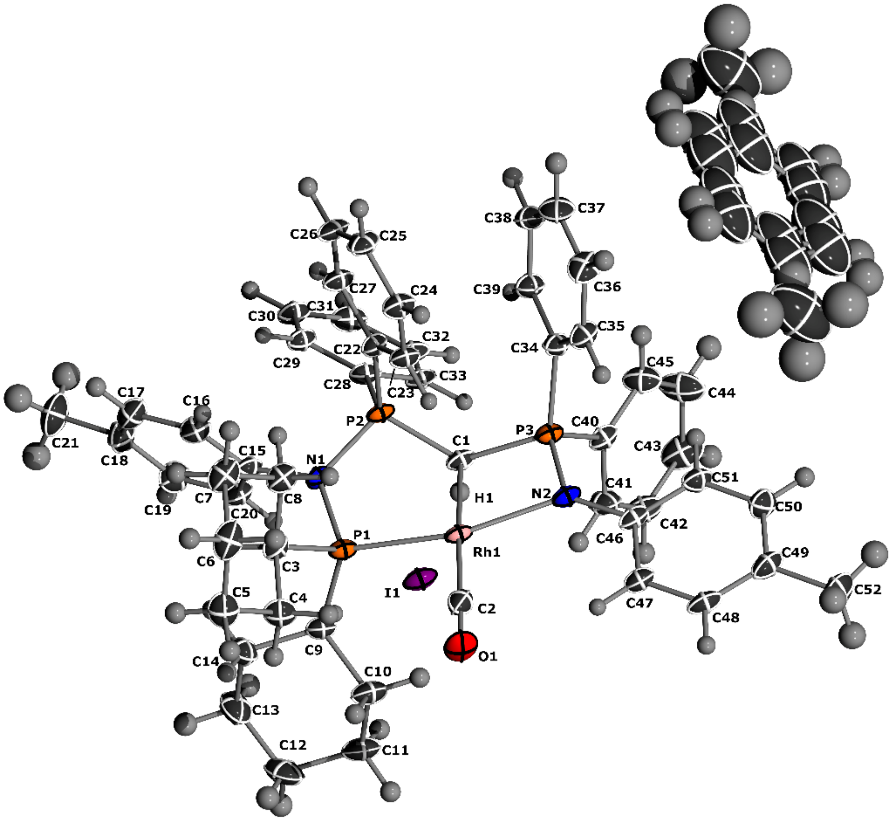

Figure S69. ORTEP diagram showing 50 % probability anisotropic displacement ellipsoids of non-hydrogen atoms for complex **5**.

4.8. Crystal structure analysis of complex 6

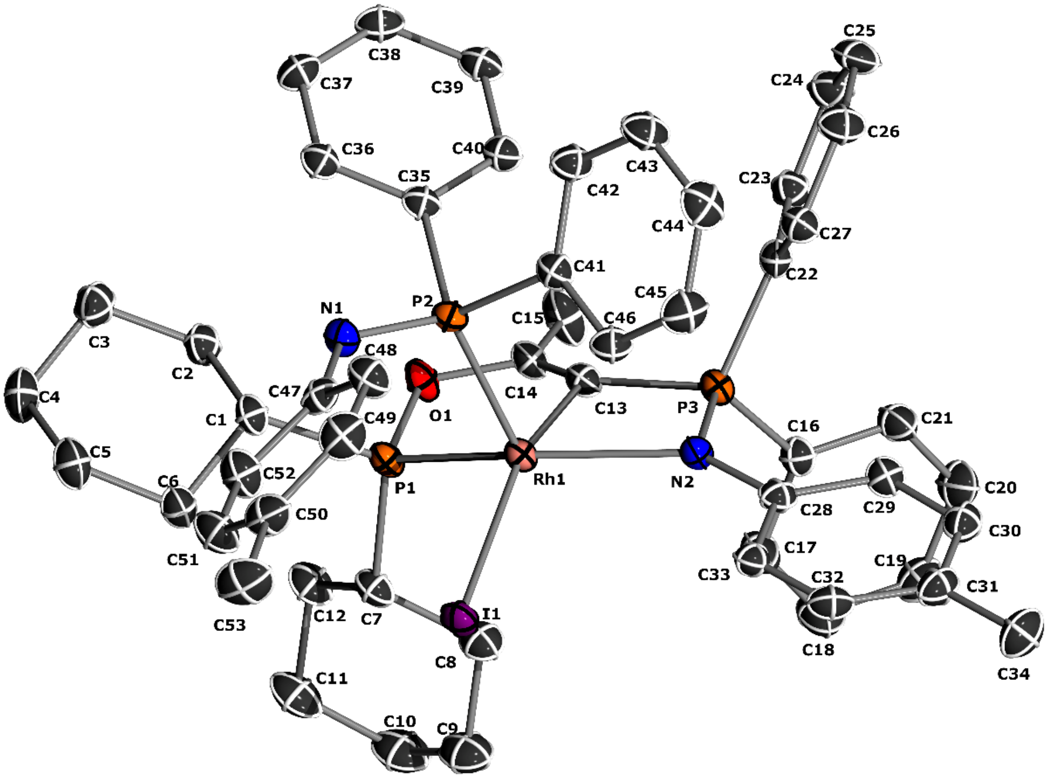

Figure S70. ORTEP diagram showing 50 % probability anisotropic displacement ellipsoids of non-hydrogen atoms for complex **6**.

4.9. Crystal structure analysis of complex 7

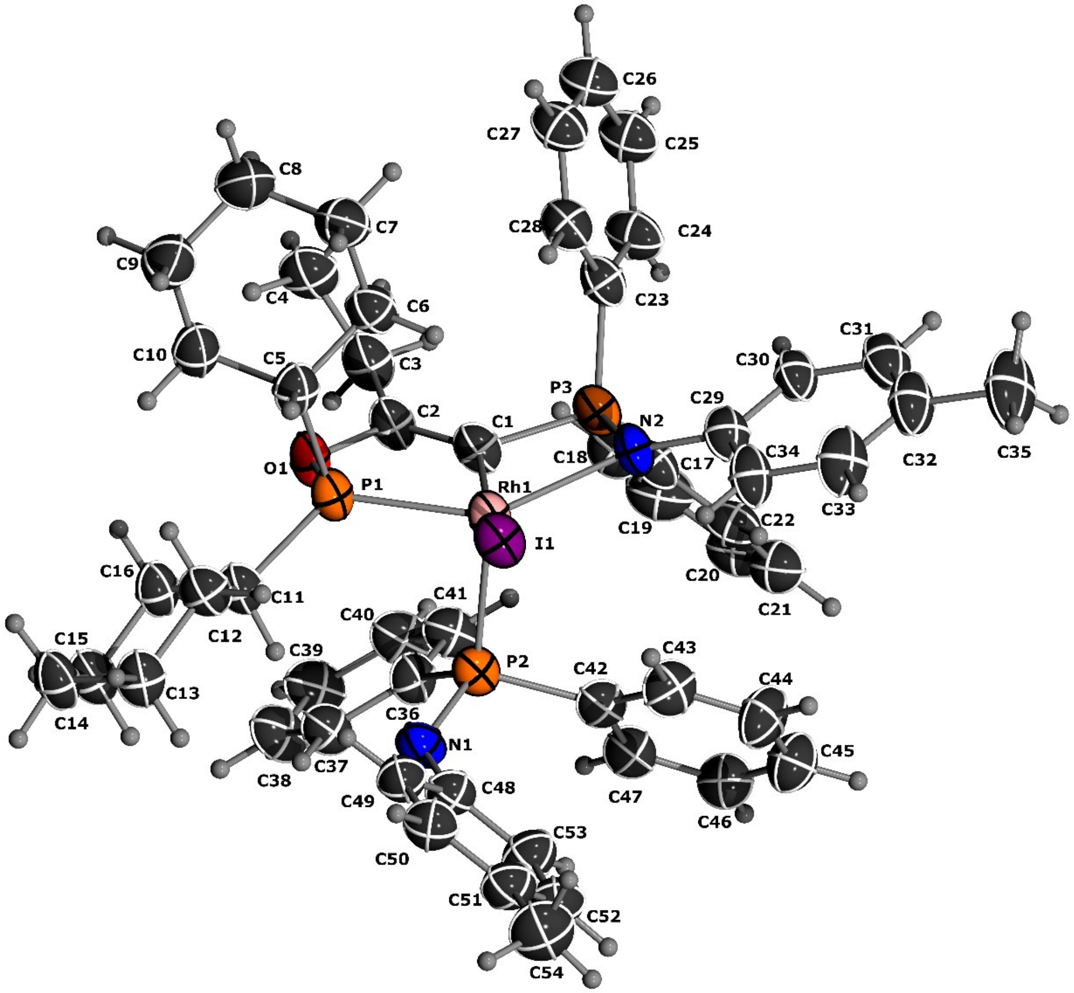

Figure S71. ORTEP diagram showing 50 % probability anisotropic displacement ellipsoids of non-hydrogen atoms for complex **7**. Only one of the two components in the asymmetric unit is shown

4.10. Crystal structure analysis of complex 8

The structure contains a disordered benzyl and cyclohexyl group. The two parts of both disorders were refined using the PART keyword and free variables which refined to 0.584 (benzyl) and 0.564 (cyclohexyl), respectively.

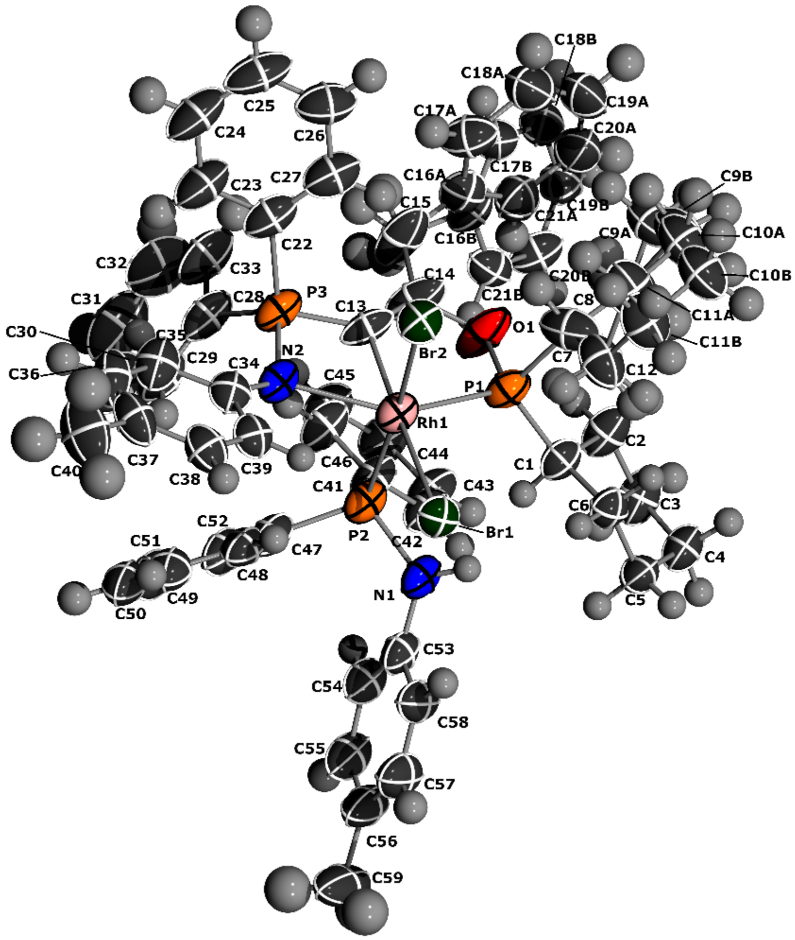

Figure S72. ORTEP diagram showing 50 % probability anisotropic displacement ellipsoids of non-hydrogen atoms for complex **8.**

4.11. Crystal structure analysis of complex 9.

The structure contains a disordered tolyl group that was refined using PART keyword and free variables which refined to 0.833.

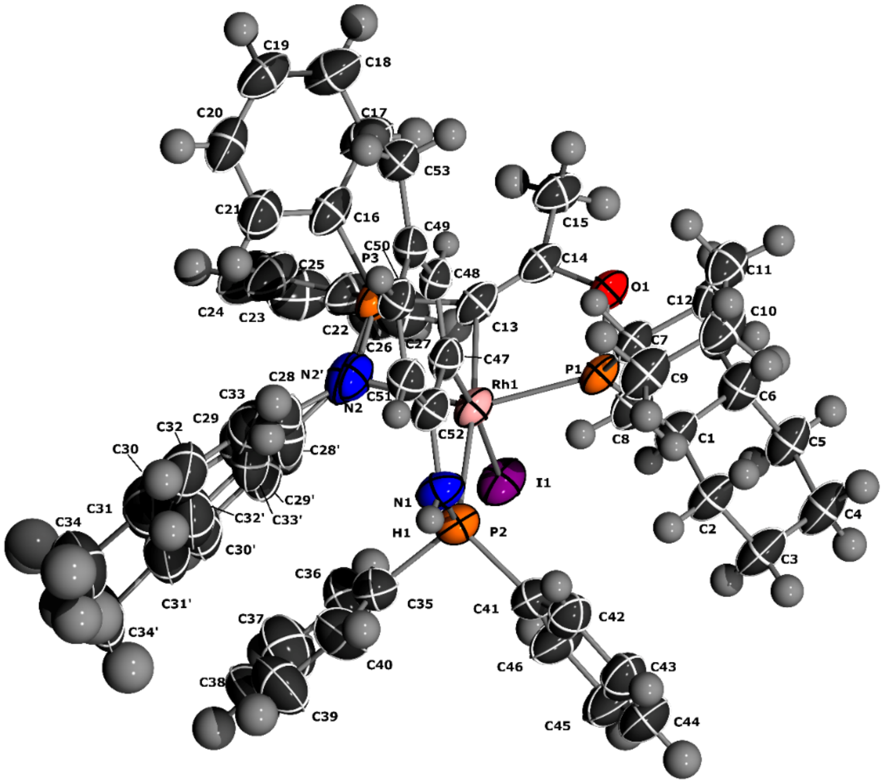

Figure S73. ORTEP diagram showing 50 % probability anisotropic displacement ellipsoids of non-hydrogen atoms for complex **9.**

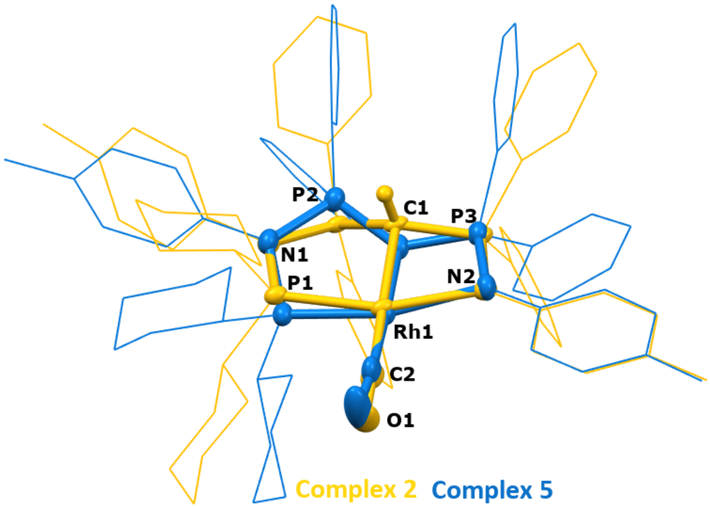

Figure S74. Comparison of the structure of complex **2** and **5.**

5. Computational studies

5.1. General information and results

All calculations were performed without symmetry restrictions. Starting coordinates were obtained from the crystal structure analyses. Geometry optimizations and NBO analysis (Ref) were carried out with the Gaussian 16 (revision C.01)[6] program package, while the Bond order analysis was done with the Multiwfn program version 3.6.[7] The geometry optimization for the ligands and rhodium complexes were performed with the PW6B95D3 functional,[8] through the def-2TZV(f) basis set for C, H, N, P, O atoms and LANL2DZ for Rh, Cl, Br, and I. The metrical parameters of the energy-optimized geometries are in good agreement with those determined by X-ray diffraction. Harmonic vibrational frequency analyses were performed at the same level of theory as the optimizations to determine the nature of the structure. The vibrational frequency analysis showed no imaginary frequencies. Population analysis and NBO analysis were performed with the same functional but using the DEF2TZVP triplet-zeta basis set and the LANL2TZ(f) ECP for Rh, Cl, Br, and I.

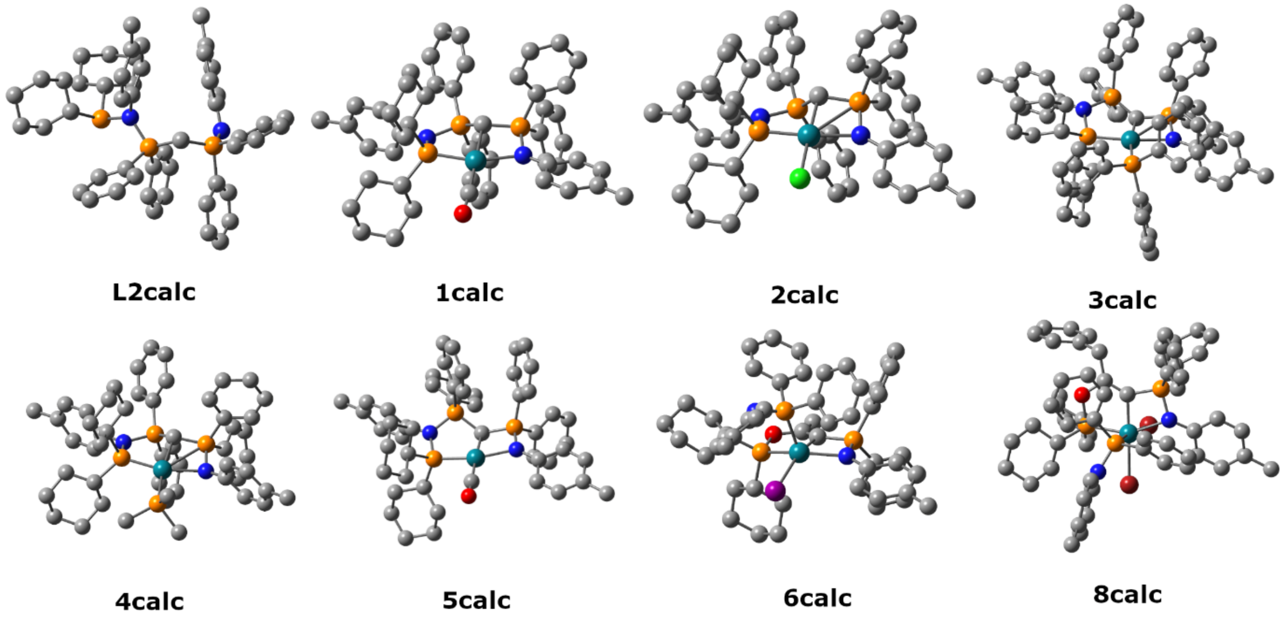

Figure S75. Optimized geometries for all calculated complexes. The optimizations were performed at the PW6B95-D3/def2svp level of theory for C, H, N, P, O atoms, and LANL2DZ for Rh, Cl, Br, and I.

Table S5 – Calculated bond distances (Å) and angles (°) for ligand $\mathbf{L2_{calc}}$.

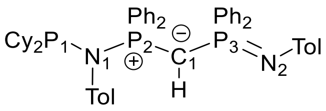

L2

	P1-N1	P2-N1	P2-C1	P3-C1	P3-N2	P2-C1-P3
$\mathbf{L2_{calc}}$	1.765	1.714	1.688	1.746	1.596	122.35

The calculations were done at the PW6B95-D3/def2svp level of theory for C, H, N, P atoms

Table S6 – Calculated bond distances (Å) and angles (°) for complexes $\mathbf{1_{calc}}$ to $\mathbf{5_{calc}}$.

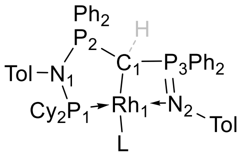

L = Cl (+C_1-H, **1**), CO (**2**), PPh_3 (**3**), PMe_3 (**4**), CO (+C_1-H, **5**)

	Rh1-P1	Rh1-N2	C1-Rh1	Rh1-L	P2-C1	P3-C1	C1-Rh1-L	P1-Rh1-N2	$\tau_{4'}$ [a]
$\mathbf{1}_{calc}$	2.173	2.215	2.124	2.453	1.738	1.771	176.50	161.91	0.105
$\mathbf{2}_{calc}$	2.223	2.163	2.109	1.867	1.661	1.698	178.95	157.37	0.099
$\mathbf{3_{calc}}$	2.206	2.207	2.092	2.266	1.654	1.696	169.51	157.52	0.194
$\mathbf{4_{calc}}$	2.203	2.200	2.120	2.269	1.657	1.690	170.07	158.51	0.184
$\mathbf{5_{calc}}$	2.250	2.139	2.157	1.877	1.742	1.783	175.48	161.41	0.117

The calculations were done at the PW6B95-D3/def2svp level of theory for C, H, N, P, O atoms, and LANL2DZ as ECP for Rh, Cl, and I.

Table S7 – Calculated bond distances (Å) and angles (°) for complexes $\mathbf{6_{calc}}$ and $\mathbf{7_{calc}}$

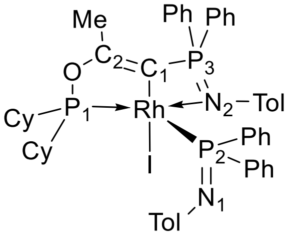

Complex $\mathbf{6_{calc}}$

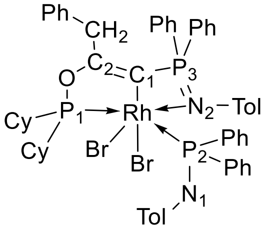

Complex $\mathbf{8_{calc}}$

	Rh1-P1	Rh1-N2	C1-Rh1	Rh1-L	P2-N1	P3-C1	Rh1-P2	C1-C3	τ_5 [a]
$\mathbf{6}_{calc}$	2.223	2.259	2.004	2.750	1.583	1.749	2.301	1.342	0.207
$\mathbf{8}_{calc}$	2.271	2.254	2.008	Br1:2.669	1.691	1.756	2.332	1.338	-
				Br2:2.600					

The calculations were done at the PW6B95-D3/def2svp level of theory for C, H, N, P, O atoms, and LANL2DZ as ECP for Rh, Br, and I.

Table S8. Comparison of the Wiberg bond order in Lowdin orthogonalized basis[a] for **L2calc** and complexes **1calc**-**8calc**

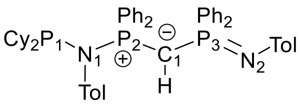

L2calc

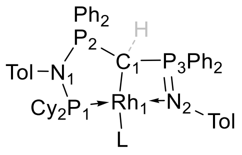

L = Cl (**1calc**), CO (**2calc**), PPh3 (**3calc**), PMe3 (**4calc**)
(P4 from PPh3 and PMe3 groups)

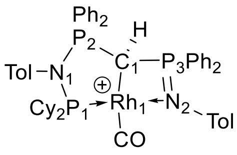

5calc

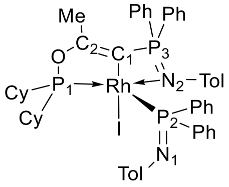

Complex **6calc**

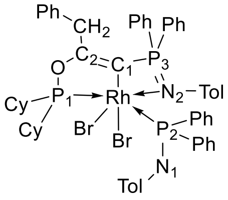

Complex **8calc**

	P2-C1	P3-C1	C1-Rh1	Rh1-L	Rh1-P1	Rh1-N2	P2-N1	P1-N1	P3-N2
**L2calc**	1.466	1.268	-	-	-	-	1.185	1.181	1.470
**1calc**	1.244	1.134	0.781	1.014	1.378	0.479	1.231	1.046	1.565
**2calc**	1.532	1.363	0.745	1.929	1.257	0.578	1.146	1.117	1.444
**3calc**	1.550	1.365	0.800	1.171	1.291	0.505	1.172	1.077	1.487
**4calc**	1.542	1.398	0.757	1.210	1.302	0.519	1.159	1.086	1.461
**5calc**	1.239	1.132	0.668	1.701	1.173	0.553	1.236	1.120	1.515
**6calc**	C2-C1:<br>1.838	1.148	0.884	P2: 1.022<br>I1: 1.331	1.155	0.462	1.688	C2-O:<br>1.541	1.478
**8calc**	C2-C1:<br>1.848	1.101	0.953.	P2: 0.949<br>Br1: 0.944<br>Br2: 0.995	1.076	0.451	1.287	C2-O:<br>1.529	1.493

[a] The Wiberg bond orders were calculated from the optimized geometries using the Multiwfn program.[4] The calculations were done at the PW6B95-D3/def2tzvp level of theory for C, H, N, P, O atoms and LANL2TZ(f) as ECP for Rh, Br, Cl, and I.

Table S9. Comparison of calculated natural charges [a] at the phosphorus atoms, carbon atoms, and rhodium atoms for ligand **L2calc** and complexes **1calc**-**8calc**.

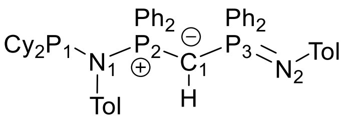

L2calc

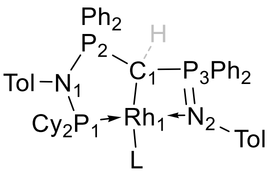

L = Cl (**1calc**), CO (**2calc**), PPh_3 (**3calc**), PMe_3 (**4calc**)
(P_4 from PPh_3 and PMe_3 groups)

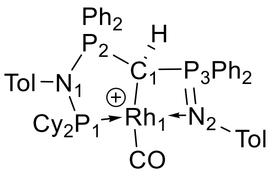

5calc

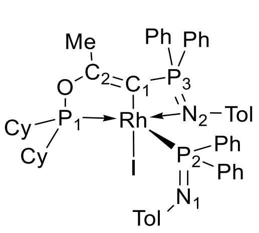

Complex **6calc**

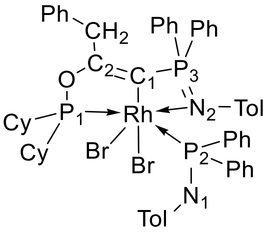

Complex **8calc**

	q (P1)	q(N1)	q(P2)	q(C1)	q(P3)	q(N2)	q(Rh1)	q(L)	
**L2calc**	1.034	-1.065	1.873	-1.427	1.833	-1.040	-	-	
**1calc**	1.449	-1.101	1.890	-1.258	1.865	-1.028	-0.040	Cl: -0.640	
**2calc**	1.460	-1.106	1.877	-1.485	1.856	-1.008	-0.079	C: 0.557 O: -0.515	
**3calc**	1.430	-1.104	1.875	-1.449	1.857	-1.044	-0.181	P4: 1.079	
**4calc**	1.436	-1.108	1.872	-1.469	1.858	-1.056	-0.213	P4: 1.074	
**5calc**	1.388	-1.091	1.885	-1.287	1.856	-1.019	-0.106	C: 0.594 O: -0.464	
**6calc**	1.565	-1.046	1.520	-0.736	1.813	-1.018	0.324	I1: -0.203	C2: 0.311
									O1: -0.778
**8calc**	1.647	-0.974	1.418	-0.573	1.825	-1.001	0.150	Br1: -0.561	C2: 0.318
								Br2: -0.468	O1: -0.783

[a] The natural charges were obtained through NBO analyses using NBO 7. The calculations were done at the PW6B95-D3/def2tzvp for C, H, N, O, P, and LANL2TZ(f) as ECP for Rh, Cl, Br, and I.

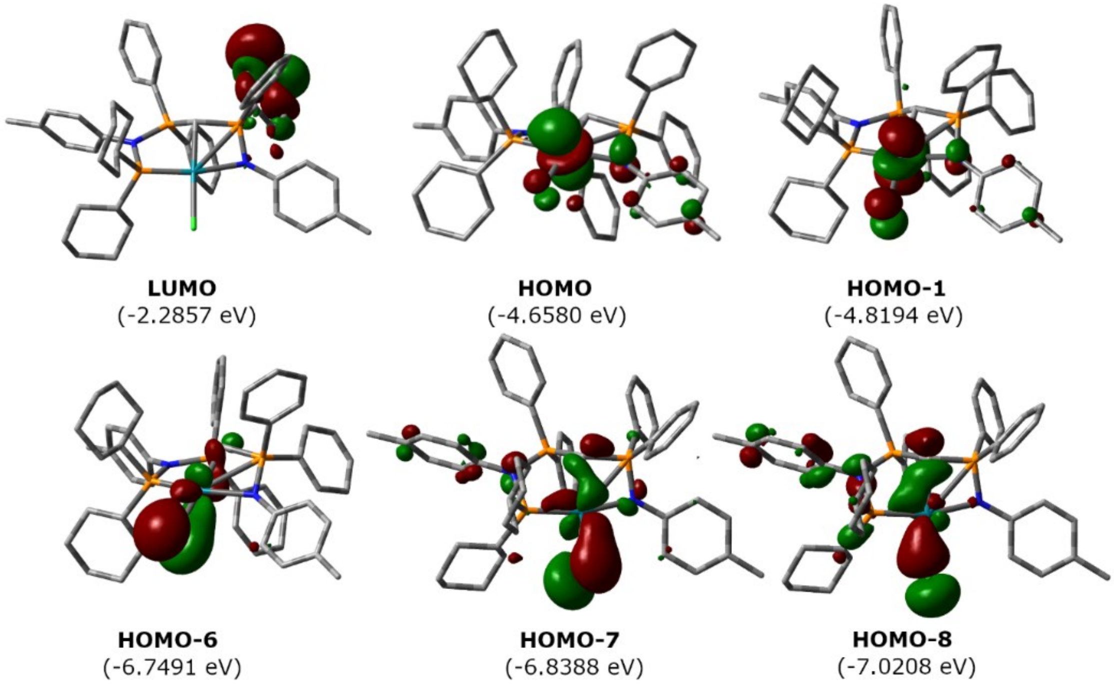

Figure S76 – NLMO diagrams of LUMO, HOMO orbitals as well as orbitals involved in the Rh-C bonding for complex $\mathbf{1}_{\mathbf{calc}}$ and their energies in parentheses.

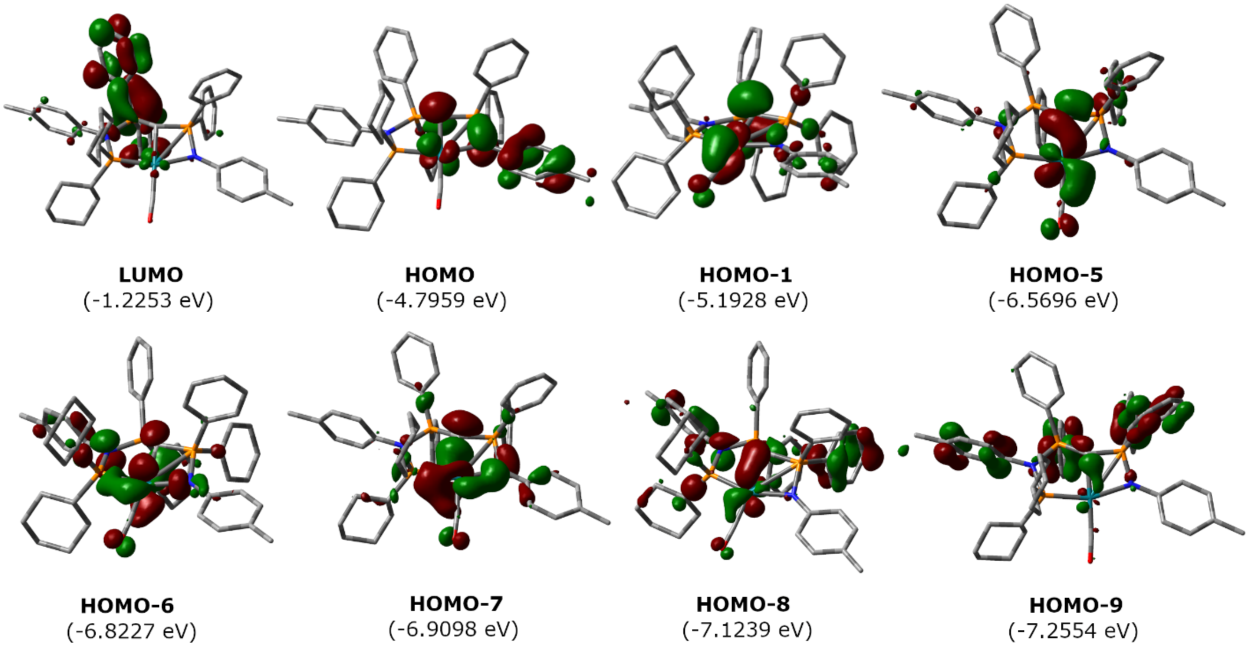

Figure S77– NLMO diagrams of LUMO, HOMO, HOMO-1 orbitals, as well as orbitals involved in the Rh-C bonding for complex $\mathbf{2}_{\mathbf{calc}}$ and their energies in parentheses.

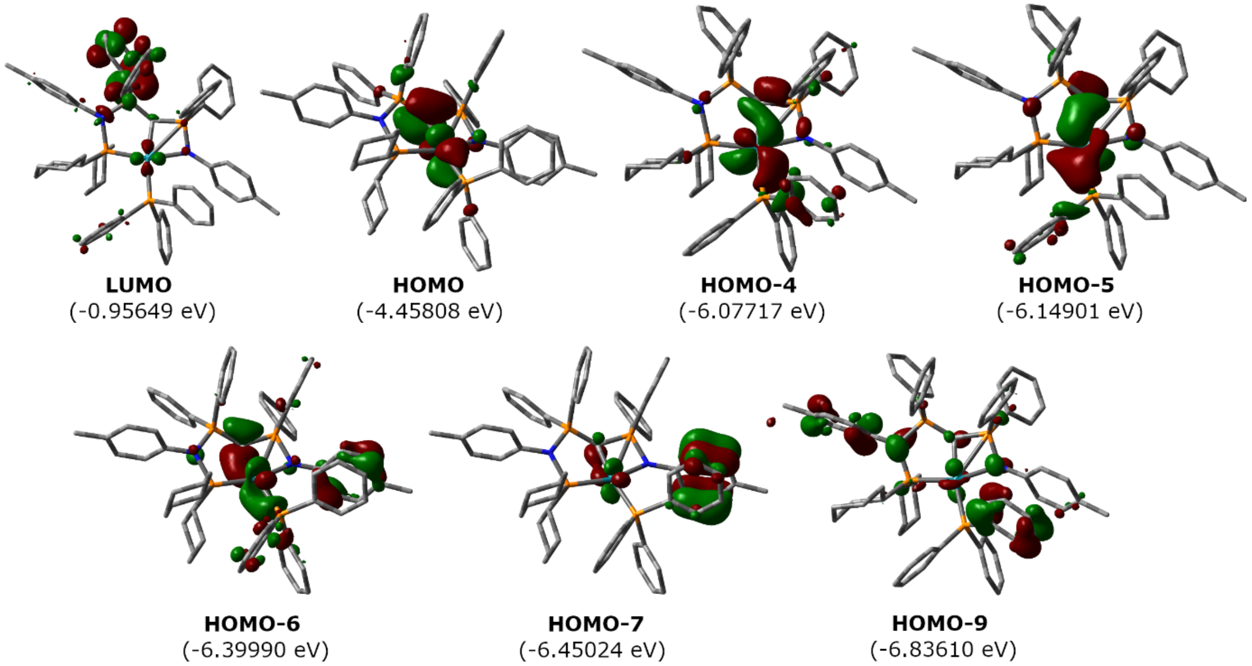

Figure S78 – NLMO diagrams of LUMO, HOMO orbitals, as well as orbitals involved in the Rh-C bonding for complex $\mathbf{3}_{\mathbf{calc}}$ and their energies in parentheses.

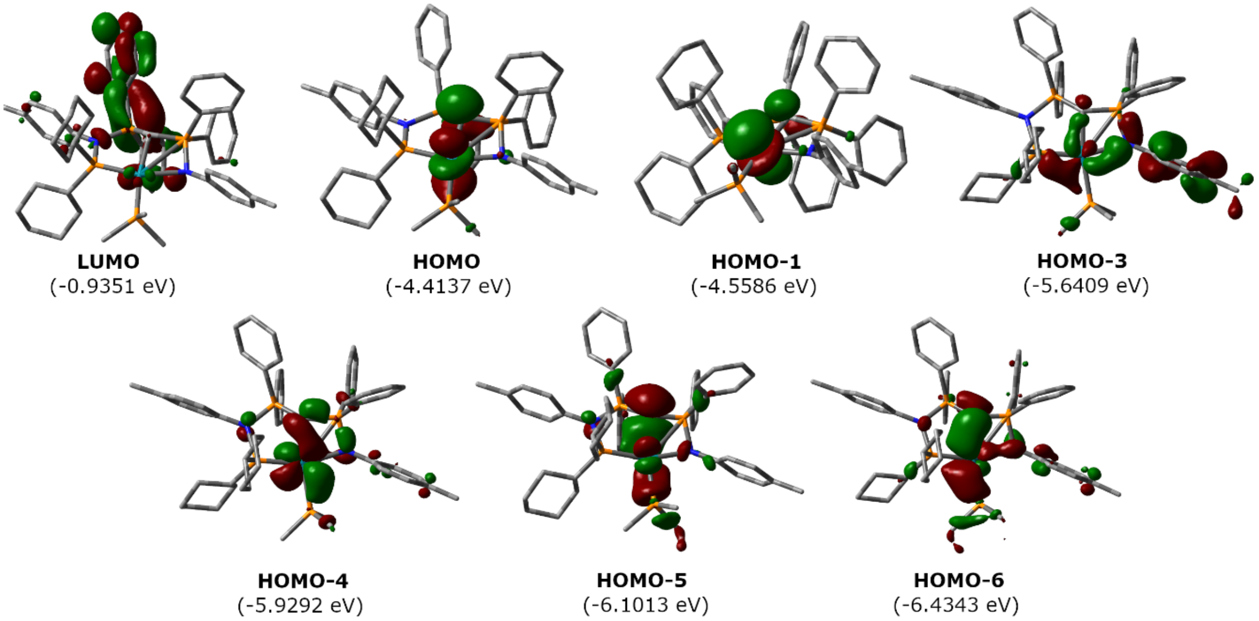

Figure S79 – NLMO diagrams of LUMO, HOMO orbitals, as well as orbitals involved in the Rh-C bonding for complex $\mathbf{4}_{\mathbf{calc}}$ and their energies in parentheses.

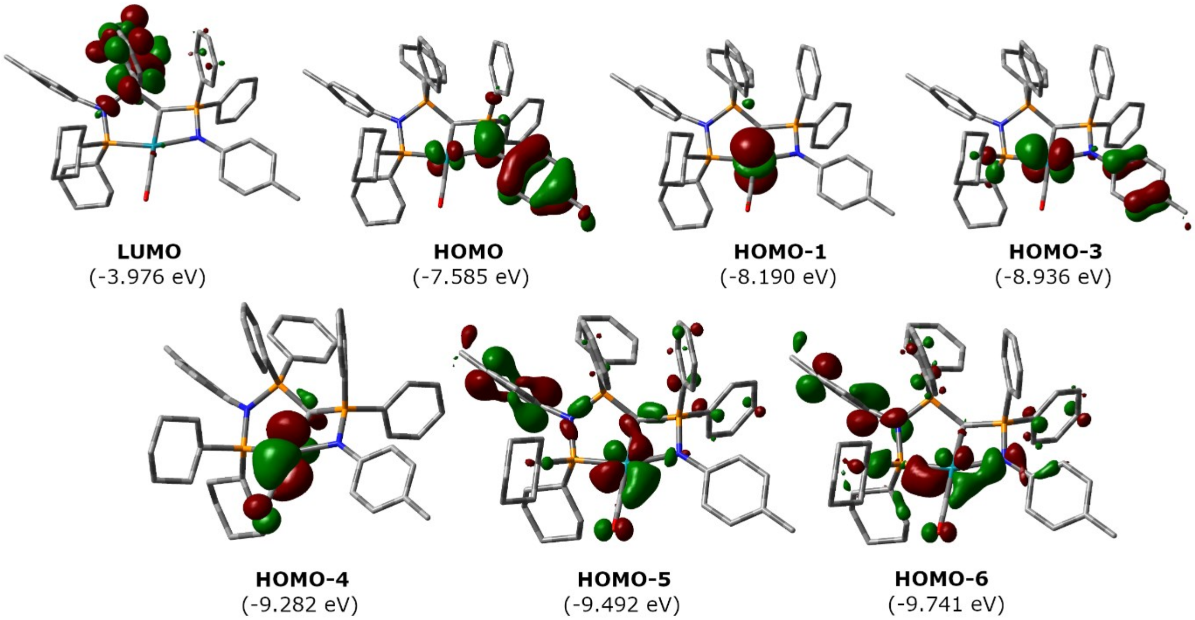

Figure S80 – NLMO diagrams of LUMO, HOMO orbitals, as well as orbitals involved in the Rh-C bonding for complex **5**$_{\mathbf{calc}}$ and their energies in parentheses.

5.2. Proposed mechanism of the formation of 6 and 7

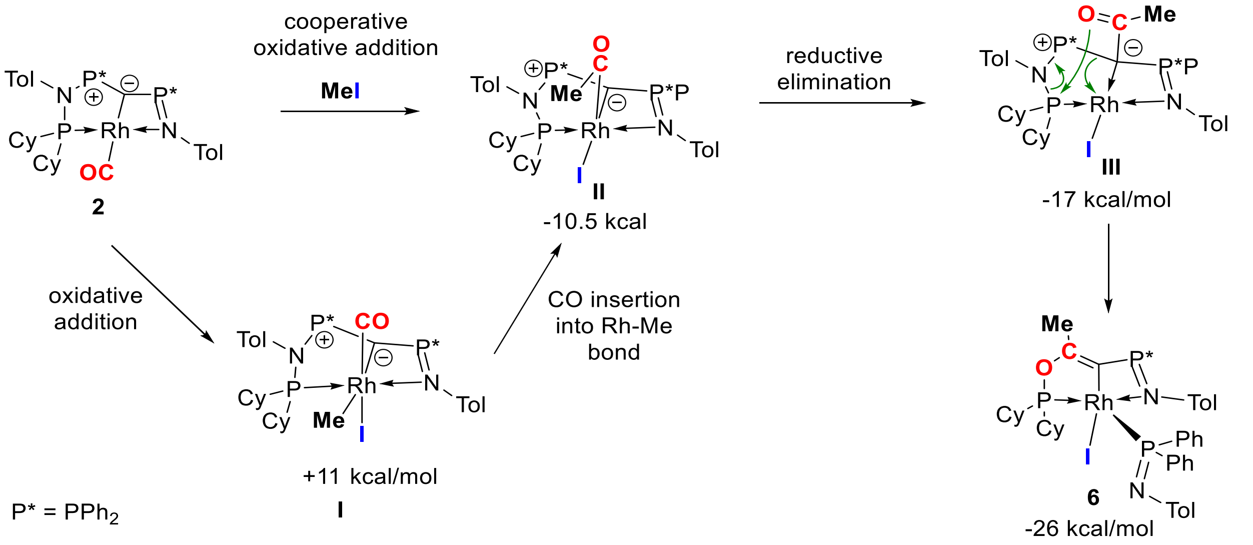

Figure S81 – Proposed mechanism for the formation of **6**. Energies have been calculated at the PW6B95D3/def2svp(C,H,N,P,O)/lanl2dz(Rh, I) level of theory.

The formation of complex **6** (and **7**) is proposed to first proceed via the formation of acyl complex **II**. Due to the high electron shift to the carbonyl ligand in **2** this is an exergonic **reaction** and the acyl complex favoured over the classical oxidative addition complex **I**. Due to the higher energy of complex **I** relative to **2**, it is reasonable to assume that the formation of **II** proceeds via a cooperative bond activation step, in which the methyl group directly is bound to the carbonyl carbon atom. In the next step, reductive elimination leads to the formation of ylide complex **III**, from which the carbonyl group might attack at the aminophosphine moiety to eliminate the amidophosphine and to form the final complex **6**. For a detailed mechanistic understanding further calculations including conformational analyses, the inclusion of solvent effects and calculations of activation barriers as well as other mechanistic pathways are required.

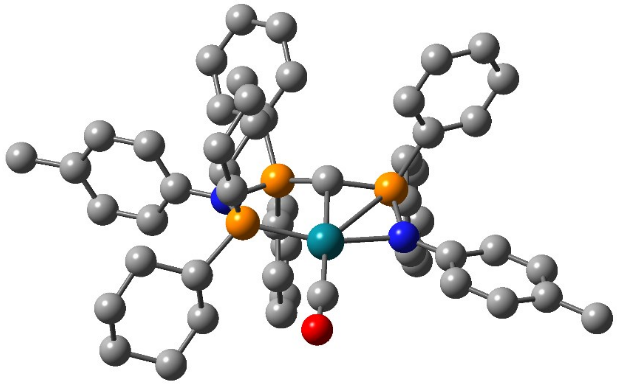

2_{calc}

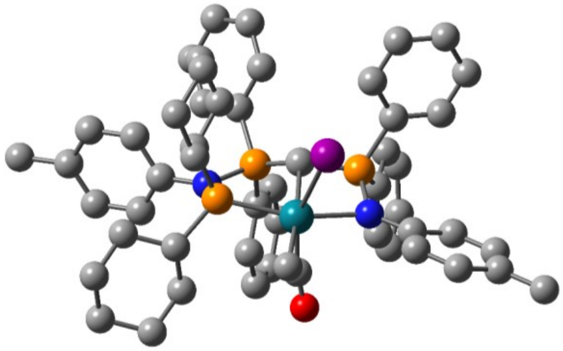

I

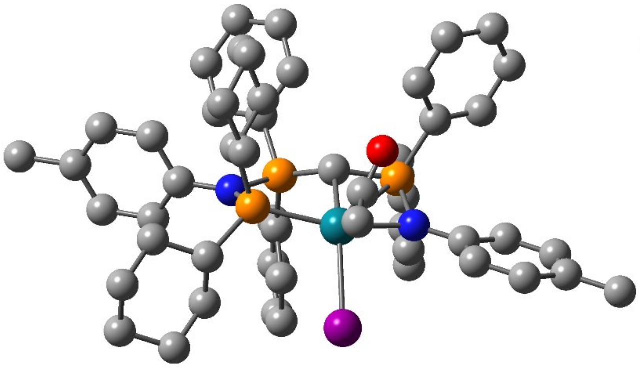

II

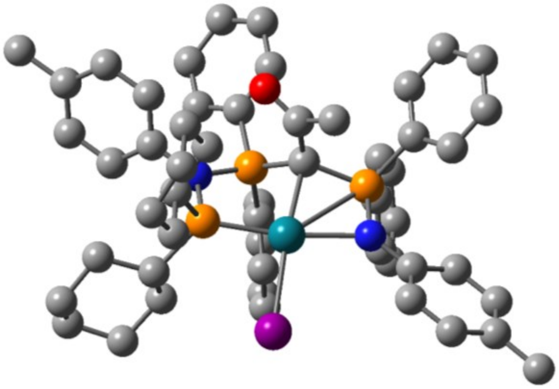

III

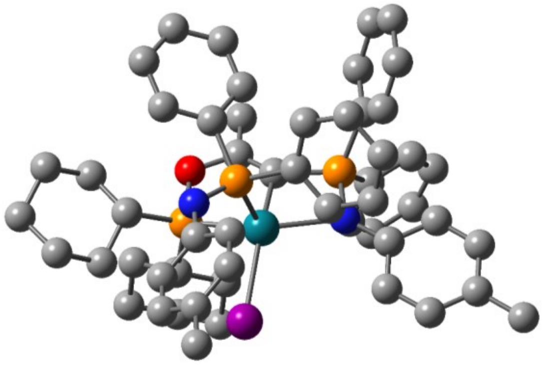

6_{calc}

Figure S82. Optimized structures of the complexes shown in Figure S81.

Table S10. Caclulcated energies of the complexes shown in Figure S81.

	Gibbs Energies from optimization [Hartree]	ΔG relative to complex **2**+ MeI [kcal/mol]
Complex **2**	-3334.822726	-
MeI	-51.282	-
Complex 2 + MeI	-3672.62238	0
Complex **I**	-3386.087181	11.00698
Complex **II**	-3386.121511	-10.5351
Complex **III**	-3386.132553	-17.464
Complex **6**	-3386.145745	-25.7419

5.3. Cartesian coordinates

Ligand L2 CyPC(H)NTol

P	-2.42068111	-0.46918461	-1.08752671
P	0.32996009	-1.15541317	-0.06661018
P	2.96624285	0.26546987	0.22272871
N	2.85115500	1.57656786	-0.68083460
N	-1.13126206	-0.27601106	0.10142889
C	-3.05797816	1.26131519	-1.26949148
H	-3.40194133	1.62627944	-0.29003914
C	0.79133660	0.10460935	-2.46546310
H	0.50965034	1.00968097	-1.93606973
C	-1.43716023	0.17220240	1.42468669
C	2.02199722	2.64140897	-0.46171237
C	0.75177499	-1.12910609	-1.81361187
C	1.75733603	3.52190803	-1.53710128
H	2.23697156	3.30678293	-2.49070091
C	-2.19341961	1.06426676	3.99595020
C	-0.78072024	-5.48662386	1.06259910
H	-1.05648760	-6.50024373	1.34738756
C	4.03151304	0.52523240	1.68692662
C	-1.98835228	2.21201624	-1.80695905
H	-1.11544076	2.24953046	-1.14220897
H	-1.62492204	1.82318044	-2.77076157
C	0.56113491	4.06029532	0.88779737
H	0.09743599	4.25887585	1.85547686
C	0.93131657	4.62275437	-1.39438764
H	0.76148979	5.27292178	-2.25402070
C	-1.99315286	1.96393315	2.94518975
H	-2.13008486	3.03112743	3.11800562
C	0.30671128	4.92293303	-0.17696116
C	3.85558036	-0.91421904	-0.84136248

C	4.40151832	-0.48490544	-2.04953754
H	4.28418710	0.56220457	-2.31991894
C	1.15198855	-2.28931658	-2.47972086
H	1.15157744	-3.24654828	-1.96413512
C	-4.82960676	2.63687419	-2.41768900
H	-5.22793174	2.99438373	-1.45581385
H	-5.67857074	2.60216390	-3.11318227
C	-0.08159854	-2.88503844	0.34075038
C	1.57391311	-2.21378545	-3.80041768
H	1.89457897	-3.11606828	-4.31648642
C	1.21053241	0.17133229	-3.78687388
H	1.24407843	1.13598603	-4.28902343
C	-1.61824000	-0.73497187	2.47108929
H	-1.48861209	-1.79982021	2.29156144
C	-1.98892535	-0.29070092	3.73513333
H	-2.13226262	-1.01755383	4.53370294
C	-1.61153516	1.53136595	1.68382437
H	-1.44180797	2.24744632	0.88897837
C	-1.08113859	-3.56663393	-0.36159193
H	-1.57810155	-3.08137513	-1.20191021
C	1.38772388	2.94912764	0.76031724
H	1.55199582	2.30726398	1.62436235
C	-3.72966308	-1.31055292	-0.04040788
H	-3.10372870	-1.93378169	0.61894358
C	-5.43057351	-3.16152235	0.02279923
H	-4.76380964	-3.80255268	0.62106067
H	-6.04963252	-3.83513562	-0.58452098
C	-2.54934240	3.61342252	-2.00495254
H	-1.76791183	4.26872687	-2.40759608
H	-2.82153817	4.03064560	-1.02249510
C	0.22644801	-4.81989896	1.75191203
H	0.74175470	-5.31120049	2.57497957
C	4.76121242	1.71237587	1.74871061
H	4.65492541	2.43352822	0.93932424
C	-4.25554514	1.23878799	-2.22476077
H	-3.92611924	0.83912440	-3.19652090
H	-5.03723727	0.55980819	-1.86133296
C	5.14631161	-2.73229287	-2.51173221
H	5.64656504	-3.44335434	-3.16717029
C	-4.57504566	-2.27573428	-0.87470303
H	-5.22533704	-1.70593411	-1.55629132
H	-3.92837404	-2.88994450	-1.51606279
C	-3.76819511	3.60698018	-2.91621782
H	-3.45617043	3.30517619	-3.92801452
H	-4.18586188	4.61804434	-3.00918487
C	3.96092479	-2.25581243	-0.47001549
H	3.50984701	-2.59122509	0.46309168
C	-1.42863533	-4.86200440	-0.00066676
H	-2.20701036	-5.38652602	-0.55202715
C	0.58128855	-3.52476927	1.38742296
H	1.37311460	-2.98996380	1.91005238
C	-6.30227412	-2.33381458	0.95757092
H	-7.03992197	-1.77677377	0.35926462
H	-6.87708175	-2.98903839	1.62519348
C	4.14894886	-0.41190930	2.71519608

H	3.57519464	-1.33637060	2.67063761
C	5.71616132	1.02291415	3.85117705
H	6.37285960	1.21754690	4.69734512
C	5.59938868	1.96050768	2.83053074
H	6.16189980	2.89120779	2.87761925
C	1.57850195	-0.55199404	0.89648568
C	-4.62512457	-0.45721916	0.85827887
H	-4.03433181	0.23787325	1.46462901
H	-5.29046973	0.15837506	0.23490694
C	1.59899937	-0.98539422	-4.45398297
H	1.93345155	-0.92935866	-5.48835803
C	-2.58696235	1.54771148	5.35935001
H	-1.75056344	2.06201111	5.85160688
H	-2.89303143	0.71878550	6.00601488
H	-3.41668436	2.26270703	5.30622707
C	-5.47126428	-1.34502332	1.76328784
H	-6.11907534	-0.72727781	2.39907678
H	-4.80016169	-1.89307509	2.44314182
C	-0.56163204	6.13667139	-0.02275121
H	-1.19313732	6.06543042	0.87070254
H	-1.22183419	6.27572747	-0.88848241
H	0.03508423	7.05529033	0.07398376
C	4.60460889	-3.16222902	-1.30306533
H	4.67892296	-4.20878443	-1.01257037
C	5.04551416	-1.39514412	-2.88117451
H	5.46355999	-1.05924087	-3.82868663
C	4.99070837	-0.16383142	3.79275809
H	5.08051831	-0.89750275	4.59200002
H	1.36959310	-0.41515480	1.95667547

Complex 1 (CyPC(H)NTol)Rh(Cl)

Rh	0.54477458	-1.43632484	-0.01780029
Cl	0.58759359	-3.71154064	-0.93323449
P	2.30064938	0.62677091	0.51685268
P	-1.61083835	-1.52622260	0.24161622
P	-0.70699646	1.22306623	-0.04154292
N	-2.04656252	0.18935665	0.03171354
N	2.59207722	-0.67139346	-0.37876768
C	2.71802317	2.19973682	-0.28270012
C	-3.36744442	0.68086130	-0.15787010
C	0.13453088	0.62170209	-2.60719956
H	0.09605553	-0.41080359	-2.25436555
C	3.25919149	2.17919145	-1.56799622
H	3.46722350	1.22311870	-2.04512351
C	-1.33247285	2.74783100	0.74639132
C	-0.20815467	1.65218396	-1.72484133
C	0.57234446	0.48947384	0.87836138
C	0.56424213	0.92556441	-3.89261444
H	0.83829734	0.11748076	-4.56749764
C	-4.24073959	0.80968311	0.92076959
H	-3.88851721	0.56685367	1.92008073
C	-0.10293932	2.98464675	-2.13851686
H	-0.31901711	3.79762531	-1.45145790
C	3.82869348	-1.17354855	-0.76419976

C	-2.35816410	-1.94194125	1.87695255
H	-3.43566930	-1.71754896	1.85049865
C	-5.99831789	1.61207815	-0.54986694
C	3.51298039	3.37712266	-2.22710567
H	3.93938165	3.36106907	-3.22800756
C	5.04259795	-0.51251802	-0.52054012
H	5.05057092	0.43716823	0.01409732
C	-5.53889599	1.26020699	0.72136705
H	-6.20821403	1.35136731	1.57579419
C	2.39971119	3.41723049	0.32496760
H	1.95837980	3.43116199	1.32114362
C	-3.80748253	1.05030058	-1.43017688
H	-3.11949215	0.98054498	-2.27025322
C	3.11367495	0.60742214	2.14249923
C	3.87959254	-2.39982112	-1.44646613
H	2.94566845	-2.93241423	-1.61473434
C	2.82265604	-0.49310617	2.95724469
H	2.12446970	-1.25243140	2.59720150
C	-2.28953444	3.57780592	0.14990357
H	-2.65198905	3.36141950	-0.85170698
C	6.25018414	-1.05179211	-0.95153595
H	7.17403670	-0.51129496	-0.74430222
C	0.64978264	2.25066314	-4.30718131
H	0.98692932	2.48529409	-5.31533823
C	5.09052540	-2.92143660	-1.86722945
H	5.09371965	-3.87788772	-2.39070201
C	-2.22519434	-2.11000981	-2.40153155
H	-2.48824484	-1.06045105	-2.59665958
H	-1.13757254	-2.20675454	-2.49627969
C	-0.88790373	3.05007843	2.03608460
H	-0.14057841	2.42074640	2.51442466
C	6.30473538	-2.26369897	-1.63483981
C	-5.10293129	1.51244836	-1.61805892
H	-5.42805257	1.79621664	-2.61819251
C	-7.41153373	2.06337013	-0.76487515
H	-7.81639105	2.55554447	0.12612979
H	-8.06448993	1.20875004	-0.98970095
H	-7.48790194	2.75957397	-1.60725584
C	-2.16221121	-3.43895111	2.13400493
H	-1.09201540	-3.67278999	2.02175509
H	-2.68280611	-4.03265072	1.37237315
C	-1.69508003	-1.12310023	2.98138556
H	-1.83569037	-0.04678179	2.79879978
H	-0.61138578	-1.31120731	2.92830304
C	0.31835274	3.27758878	-3.42936550
H	0.40634466	4.31608731	-3.74166478
C	-2.21050042	-1.51132512	4.35961893
H	-1.70700005	-0.91407507	5.13143422
H	-3.28358733	-1.27368300	4.43112649
C	-2.78627382	4.68124930	0.83075698
H	-3.53083435	5.31561642	0.35425945
C	3.43588333	-0.61819941	4.19629948
H	3.20819787	-1.47505078	4.82724987
C	-2.65343528	-3.83499766	3.52084845
H	-3.74570068	-3.70258512	3.56809468

H	-2.46256641	-4.90236873	3.69073584
C	4.04215480	1.55858878	2.56477694
H	4.29598944	2.39992799	1.92287331
C	-2.33853398	4.97100255	2.11601386
H	-2.73094935	5.83527928	2.64840283
C	-2.61096888	-2.48285489	-0.97125274
H	-2.18369324	-3.48624559	-0.79935896
C	4.34979815	0.34248699	4.62295296
H	4.83371763	0.23945855	5.59241285
C	-1.38820980	4.15457647	2.71713076
H	-1.03122226	4.37543958	3.72092809
C	-2.00362427	-2.99803076	4.61336810
H	-0.92378781	-3.20952537	4.63387500
H	-2.39685832	-3.27990307	5.59883685
C	4.65560401	1.42518814	3.80633951
H	5.38121630	2.16768239	4.13274092
C	-4.77236886	-3.46424875	-1.79494448
H	-4.41907547	-4.49404613	-1.63362340
H	-5.86060337	-3.47921742	-1.64842651
C	-4.12451574	-2.54568101	-0.76451874
H	-4.36138650	-2.90008760	0.24663492
H	-4.56547020	-1.54463621	-0.85725794
C	7.60588614	-2.85231183	-2.09462420
H	7.79749646	-3.82537524	-1.62224462
H	8.44886915	-2.19513316	-1.85318788
H	7.61345507	-3.01952809	-3.18011844
C	-4.42572802	-3.03605859	-3.21349962
H	-4.90120702	-3.70498862	-3.94292485
H	-4.83691106	-2.03020070	-3.39599520
C	-2.91786094	-3.01004782	-3.41484924
H	-2.66763875	-2.68516568	-4.43337458
H	-2.51815132	-4.02899594	-3.30611471
H	0.36267786	0.47158164	1.95092929
C	3.20761551	4.58917131	-1.61701784
H	3.40315181	5.52456703	-2.13830366
C	2.64374583	4.61033298	-0.34345981
H	2.39513418	5.55845734	0.12956833

Complex 2 (CyPCNTol)Rh(CO)

Rh	0.67405752	-1.57651355	-0.28167059
P	2.24989899	0.64876879	0.22904577
P	-1.50865571	-1.57476342	0.13924340
P	-0.73886411	1.22925517	0.00852888
N	2.67857294	-0.77753636	-0.43572750
N	-2.01921962	0.09078497	-0.02293869
O	0.64203009	-4.35232846	-1.45939800
C	-0.73048863	1.96825074	-1.65552681
C	2.76489423	1.88985014	-2.19510124
H	2.79721294	0.87043148	-2.57896936
C	2.59020435	2.09005309	-0.82472483
C	2.53026483	3.38864316	-0.31240535
H	2.38159712	3.54601355	0.75605434
C	4.15483543	-2.66394996	-0.73766086
H	3.30093444	-3.32042126	-0.58923363
C	0.59678649	0.37701450	0.50809811

C	-1.25729364	2.55195370	1.14267637
C	3.15845394	0.97188867	1.77537838
C	-0.64157086	1.07033009	-2.72601739
H	-0.62815772	0.00028842	-2.51618413
C	4.35828686	1.68415445	1.82577115
H	4.76207577	2.13850452	0.92297474
C	3.94600657	-1.27630662	-0.66383138
C	2.89739296	2.98270101	-3.04307755
H	3.03243903	2.82510406	-4.11137567
C	-2.01606047	-2.05113891	1.84648800
H	-3.09832737	-1.87095196	1.93629973
C	-2.61663012	-2.56872324	-0.95347844
H	-2.16186949	-3.56829148	-0.84068701
C	2.66804893	4.47850839	-1.16450038
H	2.63053885	5.48950592	-0.76266927
C	5.41142756	-3.19068012	-0.98090893
H	5.52738215	-4.27387143	-1.02843504
C	5.07005047	-0.45439641	-0.85071822
H	4.95404267	0.62700639	-0.83533330
C	2.64653732	0.40617136	2.94687824
H	1.70694522	-0.14233642	2.89104198
C	2.85612269	4.27538455	-2.52839287
H	2.96564085	5.12924524	-3.19469876
C	-0.66906611	3.33911187	-1.90164083
H	-0.69188427	4.04661013	-1.07604888
C	-0.50507301	2.91335293	-4.27019899
H	-0.42084892	3.28344604	-5.29037592
C	-2.43821678	-2.16659279	-2.41618459
H	-1.36776065	-2.15379120	-2.66037847
H	-2.80714169	-1.14077712	-2.55242835
C	-0.53816792	1.54114512	-4.02627249
H	-0.47318398	0.83609978	-4.85290778
C	6.32821095	-0.99689913	-1.08521229
H	7.17442935	-0.32354951	-1.22413907
C	6.53320506	-2.37278585	-1.15640606
C	-4.09226360	-2.68905201	-0.57045492
H	-4.19318587	-3.04512291	0.46244174
H	-4.57484550	-1.70460818	-0.61308217
C	4.51928672	1.26552679	4.19409020
H	5.05001472	1.38200765	5.13736123
C	3.32427206	0.55180846	4.15004822
H	2.91928652	0.10832590	5.05791898
C	-0.55932973	3.80705947	-3.20851656
H	-0.50758687	4.87836872	-3.39307238
C	5.03407535	1.83098917	3.03292054
H	5.96775171	2.38928793	3.06499666
C	-2.34317055	3.39884060	0.89500541
H	-2.91359805	3.30218237	-0.02716791
C	0.71538875	-3.29926915	-1.00052533
C	-4.66200376	-3.20589389	-2.96710219
H	-5.13973545	-2.22284558	-3.10178965
H	-5.18576720	-3.90238052	-3.63459434
C	-4.81822357	-3.63930511	-1.51665151
H	-4.40751969	-4.65370251	-1.39665172
H	-5.87959427	-3.69805337	-1.24248343

C	-3.19190893	-3.10532081	-3.34808632
H	-3.08293364	-2.76709884	-4.38670013
H	-2.73363429	-4.10446395	-3.29603089
C	-1.27701549	-1.20305611	2.87841894
H	-1.46225920	-0.13408020	2.70485811
H	-0.19856843	-1.34083272	2.71567811
C	7.89138393	-2.95915659	-1.40613016
H	7.89746545	-3.60434425	-2.29490900
H	8.22725628	-3.57704375	-0.56200635
H	8.64114686	-2.17506904	-1.56002328
C	-3.37106314	0.53172215	-0.02489326
C	-1.74537263	-3.53785295	2.08321300
H	-2.30847001	-4.15510806	1.37059748
H	-0.67829591	-3.72992527	1.89134478
C	-0.53328787	2.68745468	2.32930188
H	0.30500588	2.01427473	2.50905796
C	-3.97251365	0.96297091	-1.20824995
H	-3.39442754	0.96126885	-2.12876692
C	-2.69717962	4.36550490	1.82742063
H	-3.54556116	5.01853485	1.63239061
C	-1.65233430	-1.60274020	4.29726555
H	-1.09752382	-0.98751408	5.01786477
H	-2.72057994	-1.39440471	4.46845439
C	-2.10086225	-3.94358122	3.50878818
H	-3.18825707	-3.84280060	3.64985492
H	-1.86497117	-5.00438546	3.66348616
C	-6.04728573	1.42425732	-0.03291872
C	-1.96891329	4.49878402	3.00681546
H	-2.24755047	5.25991880	3.73350594
C	-4.10700331	0.58614651	1.16011957
H	-3.62553452	0.31253222	2.09541157
C	-1.38180044	-3.08157300	4.53684908
H	-0.29885762	-3.26278933	4.46347403
H	-1.67932622	-3.37065370	5.55326622
C	-5.29031707	1.40381032	-1.20624113
H	-5.74123818	1.73746440	-2.13995705
C	-0.88652307	3.66211003	3.25634605
H	-0.31407680	3.76550730	4.17604690
C	-5.42669877	1.01474803	1.15021314
H	-5.98304393	1.04782885	2.08608202
C	-7.48206602	1.85864535	-0.04173948
H	-7.69963693	2.50969913	-0.89512227
H	-7.74443448	2.39635144	0.87636811
H	-8.15355395	0.99178701	-0.11187867

Complex 3 (CyPCNTol)Rh(PPh3)

Rh	-0.62029841	-0.30399674	0.17529738
P	-0.69727941	2.43768312	0.25439660
P	1.24585563	-1.47892212	0.24964411
P	2.16088637	1.28398211	-0.07937396
P	-2.25555570	-1.84289662	-0.12566197
N	-1.95090318	1.43085584	0.47445889

N	2.58985707	-0.37003987	-0.08301482
C	2.52031512	1.92574427	-1.74445425
C	-0.72661740	3.26590720	-1.37692587
C	1.61005251	-2.09220412	1.95779129
H	2.63652610	-2.48830783	1.97743274
C	3.38219889	2.11886849	0.98554156
C	0.64262389	-3.21797750	2.31908129
H	-0.38091283	-2.83520541	2.19287524
H	0.75007807	-4.06305426	1.62703700
C	6.05996799	-1.11822862	-1.21716731
H	6.61146652	-1.16995852	-2.15512980
C	1.48546727	-0.94845887	2.96122089
H	2.17403806	-0.13382612	2.70216258
H	0.48074247	-0.51164701	2.85705290
C	0.60178300	1.36821305	0.46706095
C	6.72315689	-1.37281373	-0.01636893
C	-0.93962882	5.16301576	-2.85855323
H	-1.04901304	6.23696788	-2.99936417
C	-3.90280340	2.38522585	-0.69500506
H	-3.27868858	2.74636698	-1.50707554
C	-4.88720740	-3.54665576	2.57879306
H	-5.69293091	-4.27862079	2.57512296
C	4.70772316	-0.79756109	-1.24192655
H	4.21827948	-0.59009589	-2.18841831
C	-0.86887608	4.64030498	-1.56938246
H	-0.93893665	5.30810824	-0.71385050
C	-5.69438986	-0.55319832	-2.07352149
H	-6.77082179	-0.54041422	-1.91083906
C	-0.66388372	2.42309409	-2.49166253
H	-0.53804617	1.35264826	-2.32261326
C	2.77190924	3.28765706	-1.93399622
H	2.91817028	3.93917143	-1.07294521
C	-1.34973318	-6.05760537	-1.86910734
H	-1.10965661	-7.03879196	-2.27417009
C	-2.81798286	-1.68959319	2.58611809
H	-1.99769252	-0.96900298	2.56265064
C	4.70141563	2.34999420	0.58535282
H	5.02705348	2.05108032	-0.41035459
C	5.59657578	2.95238658	1.46002897
H	6.62464562	3.12651757	1.14824221
C	2.86978503	-5.04714669	-1.37360596
H	3.72869058	-5.67303766	-1.09749509
H	1.96958638	-5.66763820	-1.24162292
C	-5.27394038	2.59311423	-0.74428111
H	-5.69233157	3.13216949	-1.59486732
C	2.96762254	2.50104294	2.26214756
H	1.93077719	2.31879737	2.54519429
C	0.51721620	5.66780145	2.43186566
H	1.38335300	6.32686240	2.45015573
C	-0.67817453	3.81197181	1.44740106
C	1.59777349	-2.95353001	-0.81664627
H	0.68130270	-3.54729024	-0.66976394
C	2.78534966	-3.84243734	-0.44277176
H	3.72200098	-3.27414686	-0.50730953
H	2.69798101	-4.18469785	0.59511835

C	-3.31420396	1.68878661	0.37271462
C	2.31395760	1.11112506	-2.85913744
H	2.05475071	0.06702442	-2.70497012
C	1.70914509	-1.42723315	4.38722112
H	2.74117957	-1.79779905	4.49668700
H	1.60499205	-0.58637135	5.08557511
C	-5.54713570	1.41762401	1.30841281
H	-6.17927428	1.03007829	2.10797059
C	-7.61674358	2.28269863	0.14168676
H	-7.88258467	3.27642270	-0.23893857
H	-8.10572416	2.15092178	1.11361105
H	-8.05869644	1.54754701	-0.54738649
C	-4.50336947	-2.93741315	3.77114185
H	-5.01080636	-3.19229234	4.69991391
C	-6.13073597	2.10721663	0.24497591
C	-0.75084658	2.94365270	-3.77308014
H	-0.69748459	2.27740079	-4.63256134
C	2.41296143	1.63338546	-4.14176278
H	2.26657857	0.98389291	-5.00315930
C	0.73430146	-2.54118051	4.74301111
H	0.90671063	-2.89812441	5.76696980
H	-0.29039001	-2.13960843	4.71780908
C	-0.88529400	4.31837107	-3.96016495
H	-0.94782638	4.72901301	-4.96642691
C	3.86517070	3.10880746	3.13362467
H	3.53509326	3.41239257	4.12529388
C	2.83920016	3.81594509	-3.21815621
H	3.02553803	4.87923850	-3.35542592
C	3.97187332	-0.72213769	-0.05905051
C	-5.15534030	0.03097779	-3.21378584
H	-5.80883253	0.49255157	-3.95185334
C	1.64078584	-2.54937241	-2.28749757
H	2.50872807	-1.89538813	-2.43940545
H	0.74405348	-1.95843622	-2.52560238
C	-1.65450785	-5.00192271	-2.72136518
H	-1.65720184	-5.15441406	-3.79919998
C	5.98676023	-1.26662297	1.16636103
H	6.48238050	-1.42707828	2.12304906
C	2.96709221	-4.61420163	-2.82906596
H	3.03460483	-5.48959149	-3.48842985
H	3.89683747	-4.04096540	-2.97172144
C	0.83655354	-3.69302799	3.75354816
H	0.09702677	-4.46944040	3.99092747
H	1.82681285	-4.16541544	3.84790026
C	2.67675550	2.98775650	-4.32391475
H	2.74108087	3.40190437	-5.32829768
C	-4.17528218	1.21830644	1.37601935
H	-3.74006161	0.69662167	2.22286006
C	4.63958635	-0.93721797	1.14960733
H	4.09809926	-0.81149926	2.08273114
C	-4.86552288	-1.13749601	-1.12053848
H	-5.30326320	-1.55999118	-0.22010894
C	5.17864719	3.33159093	2.73367968
H	5.88194107	3.80628790	3.41588451
C	1.77429825	-3.75060162	-3.21232531

H	0.86045524	-4.35510332	-3.15017918
H	1.85922531	-3.41191545	-4.25376800
C	0.42742250	4.66649610	1.47464601
H	1.22831702	4.53575412	0.74529988
C	-3.77570811	0.05189193	-3.39079415
H	-3.34068149	0.53591758	-4.26373510
C	-0.50048630	5.82040564	3.37122633
H	-0.43251048	6.60451921	4.12331866
C	-1.36581794	-5.85084381	-0.49341964
H	-1.14063474	-6.67128932	0.18581643
C	-2.94721666	-0.52156930	-2.43508333
H	-1.86311484	-0.48051405	-2.53999427
C	-1.69096201	3.96281207	2.39323964
H	-2.54490928	3.28867769	2.37770194
C	8.17284019	-1.75367814	0.00897488
H	8.29228639	-2.83809105	0.13900440
H	8.69968149	-1.27031731	0.83993265
H	8.67705270	-1.47758539	-0.92315485
C	-4.23566481	-3.22877823	1.39339393
H	-4.51953411	-3.72757092	0.46723475
C	-1.68339748	-4.59957191	0.02246754
H	-1.71583192	-4.45926224	1.10024257
C	-3.20279290	-2.28576291	1.38418198
C	-3.46519922	-2.01255492	3.77483395
H	-3.15475122	-1.54115681	4.70579097
C	-1.60020548	4.96966787	3.35075973
H	-2.39373537	5.08759710	4.08641821
C	-1.97422195	-3.52601381	-0.82722441
C	-3.48318509	-1.14415477	-1.30232937
C	-1.95448079	-3.74479625	-2.20668994
H	-2.18946481	-2.93083667	-2.88862736

Complex 4 (CyPCNTol)RhPMe3

Rh	0.69060961	-1.25670889	-0.27372584
P	2.03883952	1.10836220	0.09135820
P	-1.43813552	-1.49642538	0.24014775
P	-0.99715341	1.38226107	0.02677972
N	2.60872449	-0.23925438	-0.62955896
N	-2.14415113	0.11504846	0.10525982
C	-1.14490809	2.05828325	-1.65864452
C	2.27453646	2.39270930	-2.35902014
H	2.32795932	1.37790278	-2.75043435
C	2.20134472	2.57376469	-0.97736567
C	2.09623729	3.86459759	-0.45298324
H	2.03091033	4.00849935	0.62571364
C	4.52982628	-1.19854130	0.54823514
H	3.91515914	-1.29252105	1.44210083
C	0.44503550	0.70702202	0.48505170
C	-1.60951225	2.68392605	1.14524183
C	3.03402241	1.52003365	1.56027208
C	-0.98213205	1.13480416	-2.69860566
H	-0.82089978	0.08521855	-2.44595336
C	4.29030729	2.11760048	1.44000837

H	4.65005227	2.43080386	0.46035578
C	3.94362136	-0.64626481	-0.60325569
C	2.25949170	3.49335029	-3.20727801
H	2.31045238	3.34749861	-4.28475563
C	-1.81139494	-1.99631790	1.98215394
H	-2.89559944	-1.91007401	2.15010720
C	-2.56928700	-2.57575824	-0.75581217
H	-2.05937816	-3.54479998	-0.65022061
C	2.08148323	4.96352488	-1.30464344
H	2.00606655	5.96830703	-0.89239493
C	5.85388557	-1.61003161	0.55661600
H	6.27434373	-2.02892720	1.47128285
C	4.75541893	-0.52841734	-1.73829955
H	4.32577645	-0.09668608	-2.64005644
C	2.58363251	1.10547650	2.81472368
H	1.60717131	0.62515772	2.88112646
C	2.16845661	4.77873351	-2.68146172
H	2.15859815	5.64003626	-3.34726510
C	-1.26041595	3.41551830	-1.95690472
H	-1.34183025	4.14810198	-1.15702470
C	-1.12380279	2.91629736	-4.31370610
H	-1.11924290	3.25214423	-5.34919563
C	-2.49238551	-2.19806768	-2.23310647
H	-1.43489710	-2.13087083	-2.52727974
H	-2.91492888	-1.19349427	-2.36956791
C	-0.98069299	1.56118077	-4.01796276
H	-0.85756806	0.83611205	-4.82070218
C	6.08138533	-0.94853745	-1.72420430
H	6.68317993	-0.84322820	-2.62706381
C	6.65974865	-1.49822864	-0.58073161
C	-4.00997272	-2.78869084	-0.28699933
H	-4.02394763	-3.13694720	0.75365348
H	-4.56765971	-1.84555749	-0.30995106
C	4.62805690	1.89456352	3.81565180
H	5.25080317	2.04250883	4.69631346
C	3.37802810	1.29447163	3.93969814
H	3.02253311	0.97130965	4.91672599
C	-1.25133878	3.83945403	-3.28369088
H	-1.33623890	4.90088552	-3.50912152
C	5.08379703	2.30426917	2.56605847
H	6.06378405	2.76762485	2.46796088
C	-2.79177391	3.40301843	0.93820356
H	-3.39643975	3.21792158	0.05211288
C	-4.68120539	-3.37080856	-2.63969294
H	-5.22183265	-2.41763941	-2.74842096
H	-5.20357832	-4.10131939	-3.27123305
C	-4.72161443	-3.79951676	-1.17995612
H	-4.23561847	-4.78271154	-1.07904475
H	-5.75893800	-3.92870612	-0.84426231
C	-3.24544487	-3.18822582	-3.11083361
H	-3.21945860	-2.85813484	-4.15772987
H	-2.73334670	-4.16297304	-3.07959725
C	-1.07285002	-1.07890350	2.95371067
H	-1.37636020	-0.03381564	2.79893606
H	-0.00445753	-1.11001679	2.69299833

C	8.08741447	-1.95953856	-0.56489749
H	8.15784055	-3.04711844	-0.42690889
H	8.65075312	-1.49708239	0.25598477
H	8.59747032	-1.71165237	-1.50212403
C	-3.52958065	0.41727330	0.16475189
C	-1.39511269	-3.45232009	2.19681085
H	-1.96962865	-4.11992130	1.53928803
H	-0.33692101	-3.54897146	1.90305600
C	-0.84528766	2.93452627	2.28663811
H	0.07048295	2.36297541	2.43280943
C	-4.22785292	0.77010377	-0.99169595
H	-3.69664947	0.81467838	-1.93909667
C	-3.19944676	4.35407420	1.86554389
H	-4.12261754	4.90647934	1.70097441
C	-1.27994068	-1.50644285	4.39848757
H	-0.72486565	-0.83843714	5.07053446
H	-2.34343300	-1.40373336	4.66739904
C	-1.57492736	-3.88268795	3.64764914
H	-2.64865551	-3.88228048	3.89141346
H	-1.22817509	-4.91659706	3.77707666
C	-6.28325004	1.02812228	0.27536721
C	-2.43142497	4.59908353	3.00098611
H	-2.75403037	5.34647868	3.72384083
C	-4.21283281	0.41277129	1.38281343
H	-3.66147661	0.20166721	2.29567183
C	-0.84619762	-2.95092101	4.60551340
H	0.23723042	-3.02689124	4.42670130
H	-1.01487208	-3.26310906	5.64459052
C	-5.58278569	1.07177131	-0.93188601
H	-6.10877798	1.34476985	-1.84597732
C	-1.25317985	3.89104438	3.20967601
H	-0.64786155	4.08202408	4.09376106
C	-5.56901057	0.70155157	1.43143370
H	-6.08241648	0.68957503	2.39218981
C	-7.75456193	1.31113358	0.33160397
H	-8.08467603	1.89481715	-0.53433611
H	-8.02274895	1.86327131	1.23966819
H	-8.33456702	0.37793480	0.33829729
P	1.33078340	-3.31292614	-0.98674313
C	0.18413704	-4.70742935	-1.33370784
H	-0.36301159	-4.96766078	-0.41998930
H	-0.53842612	-4.42107872	-2.10552233
H	0.74013265	-5.58945020	-1.67619741
C	2.49926161	-4.16249412	0.14685031
H	2.84974574	-5.11646855	-0.26911132
H	3.35867725	-3.50876654	0.32702449
H	1.99867682	-4.34500702	1.10492972
C	2.29800475	-3.28357672	-2.54465560
H	3.11441791	-2.56190151	-2.43298891
H	2.71128275	-4.26989982	-2.79323735
H	1.65001659	-2.94199189	-3.35971252

Complex 5 [(CyPC(H)NTol)RhCO][I]

Rh	0.63416275	-1.43573802	0.18562380

P	-1.60869703	-1.42959392	0.01148593
P	-0.77506964	1.39405370	0.06950222
P	2.36759474	0.72117932	-0.23424360
O	0.90208093	-4.22852738	1.30064719
N	2.68202593	-0.84265248	0.01709242
N	-2.02594450	0.27742278	-0.16940902
C	3.91831795	-1.48379433	-0.09574472
C	0.62374322	0.58219750	-0.57636918
H	0.47185359	0.46185440	-1.65740085
C	-2.36568260	3.60727263	-0.73381593
H	-3.13932419	3.27279656	-0.04688689
C	-1.17223902	2.88500392	-0.87694353
C	3.97023017	-2.86588473	-0.31310147
H	3.03821508	-3.41801825	-0.40288375
C	3.21343766	1.37728061	-1.69239071
C	-0.42646737	4.48454146	-2.53388643
H	0.33351640	4.82078336	-3.23560075
C	-0.60869241	1.85128381	1.80448558
C	0.05370689	0.98408907	2.68117200
H	0.46826403	0.04568253	2.30580585
C	2.71997084	1.79159247	1.17007673
C	-3.35049690	0.68941279	-0.51392291
C	-2.57871368	4.75346851	-1.48655475
H	-3.50964259	5.30414031	-1.37176503
C	-2.40010042	-1.29479456	2.67847702
H	-2.43713274	-0.20646329	2.55155410
H	-1.36470960	-1.53286658	2.96587991
C	-4.33187979	0.84224956	0.46293345
H	-4.08413326	0.66396340	1.50642944
C	-0.20667630	3.33399453	-1.78523124
H	0.72522218	2.78667995	-1.91481252
C	-1.61183273	5.19405012	-2.38626876
H	-1.78600674	6.09272364	-2.97434081
C	-2.70782303	-2.00762309	1.36503447
H	-3.73415417	-1.76264162	1.04924717
C	0.19040164	1.32260199	4.01912201
H	0.72082601	0.65071373	4.69024989
C	-3.66935458	0.94948690	-1.84751559
H	-2.89536226	0.85825555	-2.60699099
C	-4.95432911	1.33489144	-2.19626957
H	-5.18628344	1.53557118	-3.24097810
C	3.43099375	1.25020395	2.24222589
H	3.76772055	0.21584042	2.19809516
C	5.18369937	-3.52336411	-0.43326873
H	5.18483459	-4.60004985	-0.60021964
C	-1.13380295	3.05419002	2.28714104
H	-1.62105582	3.75645254	1.61796956
C	6.40118762	-2.83933248	-0.35855313
C	5.13446321	-0.79158965	-0.02477329
H	5.14077308	0.28588359	0.13814193
C	-0.34258975	2.51614056	4.49777179
H	-0.23475622	2.77721072	5.54860311
C	-2.27743485	-2.15699868	-1.55490470
H	-2.07781945	-1.34816605	-2.27664635
C	-1.45638833	-3.36965699	-2.00547181

H	-0.38789252	-3.11832076	-1.99183831
H	-1.58891479	-4.19393419	-1.28815252
C	-3.77642184	-2.46824481	-1.58875336
H	-4.37322903	-1.60776663	-1.26693919
H	-3.99458008	-3.28185243	-0.88387419
C	2.24672489	3.10514235	1.23054636
H	1.66215011	3.51945701	0.40831248
C	-5.95622565	1.47525184	-1.22939907
C	3.76190890	2.65902539	-1.76802581
H	3.74618135	3.31879498	-0.90363289
C	-2.61218419	-3.52276729	1.56678391
H	-2.80983460	-4.06652615	0.63613748
H	-1.58857672	-3.77990086	1.86810872
C	2.49075562	3.87435645	2.35979456
H	2.11255822	4.89283018	2.41450591
C	-3.35525229	-1.73916954	3.77810613
H	-3.10683895	-1.22639827	4.71581262
H	-4.37760078	-1.42714431	3.51352267
C	0.78898902	-3.17694870	0.86953910
C	6.34515272	-1.46122952	-0.15902266
H	7.27157926	-0.89114390	-0.10135227
C	-5.61807339	1.22919620	0.10331466
H	-6.37550871	1.34393107	0.87692939
C	3.28310191	0.51862838	-2.79592074
H	2.88723863	-0.49370563	-2.71811459
C	-3.57890210	-3.97662493	2.65506682
H	-4.61049773	-3.78342699	2.32420696
H	-3.49441093	-5.06123686	2.79419375
C	3.68611778	2.03187218	3.36330367
H	4.24655854	1.61414924	4.19681149
C	-1.00623428	3.37793874	3.63239065
H	-1.41778589	4.31489040	4.00101364
C	-1.88896365	-3.82427030	-3.39363431
H	-1.63588101	-3.03533726	-4.11865321
H	-1.31602575	-4.71181652	-3.68912248
C	3.21558649	3.33904510	3.42228801
H	3.40840258	3.94608554	4.30453651
C	-3.31883234	-3.24920636	3.96632952
H	-4.04626607	-3.55859532	4.72664296
H	-2.32815332	-3.53966383	4.34709254
C	3.87868446	0.95163100	-3.97224274
H	3.94064990	0.28109352	-4.82654413
C	7.70704572	-3.56565105	-0.48059478
H	7.88346225	-4.21684820	0.38586829
H	8.54826969	-2.86798315	-0.54675127
H	7.72850788	-4.20553991	-1.37130818
C	-4.19276213	-2.90451147	-2.98853803
H	-5.26576637	-3.13272519	-3.00167049
H	-4.04521401	-2.06288894	-3.68324629
C	4.40850984	2.23679781	-4.05206265
H	4.87883257	2.57324564	-4.97377742
C	-3.38363902	-4.10469529	-3.45903928
H	-3.61816957	-4.96877865	-2.81912784
H	-3.67460101	-4.38756755	-4.47811320
C	4.35324688	3.08653626	-2.95220805

H	4.78418633	4.08377577	-3.01071989
C	-7.35093462	1.85835724	-1.61811062
H	-7.35336582	2.60248055	-2.42206818
H	-7.90911047	2.26465497	-0.76880785
H	-7.90344362	0.98358006	-1.98672088

Complex 6 (CyPOCMeCPNTol)Rh(PPh2NTol)(I)

I	1.48264223	0.87875795	-2.54429427
Rh	0.09509281	-0.23378029	-0.44676940
P	-2.59818682	-0.75580125	0.34802047
P	1.01358622	1.01192608	1.25595087
P	1.57136210	-1.86607679	-0.13366242
O	0.78733355	-2.89293490	0.96309333
N	-2.06322365	0.39771192	-0.65858259
N	2.50919396	1.50348358	1.08917740
C	-1.03871593	-1.50008567	0.61552232
C	-4.20287306	1.52450599	-1.11675233
H	-4.70242806	0.99543190	-0.30661623
C	-0.70544786	2.98547605	0.32241005
H	-0.41493239	2.62374080	-0.66183587
C	-0.55523435	-2.65647457	1.09539992
C	-3.36136592	-0.17016026	1.88606451
C	-0.21038312	2.35132127	1.46414644
C	-2.27005940	1.98543409	-2.45744419
H	-1.22838840	1.78920561	-2.70086929
C	3.03569224	2.59117259	0.42498322
C	1.02346507	0.07411096	2.81661755
C	-2.84877041	1.27931850	-1.39134465
C	2.22886231	-0.16257478	3.47868918
H	3.14089119	0.26005700	3.06228884
C	-4.92889341	2.44868543	-1.86027287
H	-5.97799865	2.61409573	-1.61534799
C	-3.80311097	-1.88482157	-0.40477691
C	-3.41029829	1.19765136	2.15268340
H	-3.05593873	1.90974804	1.40899693
C	-3.00244826	2.90872117	-3.18333798
H	-2.51256683	3.43956037	-3.99973220
C	3.63146807	-2.79335965	1.58413540
H	2.86369709	-2.88553773	2.35989682
H	3.68593804	-3.76843199	1.08136592
C	1.68674432	-2.92989315	-1.62678309
H	2.31952989	-2.33319213	-2.30478353
C	-3.90184760	1.64353683	3.37614365
H	-3.93957758	2.71143040	3.57762508
C	-0.16746755	-0.38368104	3.38259083
H	-1.11263114	-0.15094658	2.90465765
C	-0.53346040	2.85010078	2.72819625
H	-0.13185118	2.37862713	3.62293993
C	3.23539117	-1.70051353	0.59142375
H	3.15854280	-0.74739688	1.13230273
C	-0.15086561	-1.10947916	4.56637639
H	-1.09006071	-1.45005576	4.99931873
C	4.29396697	-1.46576120	-0.48911275
H	4.41161246	-2.36238173	-1.11423392

H	3.96919583	-0.65489811	-1.15516147
C	-3.79354099	-1.08665048	2.85171093
H	-3.75465773	-2.15551143	2.64697886
C	-4.34870808	3.16867705	-2.90195224
C	2.56947230	3.90603355	0.60645022
H	1.74809991	4.09138276	1.29270804
C	3.16764648	4.97436046	-0.04553128
H	2.78213762	5.97957596	0.12590235
C	4.25265034	4.79646455	-0.90563941
C	-1.26440740	-3.76044149	1.79145766
H	-0.95946919	-4.73003047	1.38061813
H	-2.34817961	-3.65687215	1.68710496
H	-1.00481095	-3.75854220	2.85941561
C	4.73073925	3.49439129	-1.06988161
H	5.58143734	3.31542274	-1.72736872
C	-5.14778958	-1.94923789	-0.03617523
H	-5.52329976	-1.33052466	0.77664343
C	5.62830350	-1.13676073	0.16757075
H	6.39549055	-0.98222172	-0.60181796
H	5.53229011	-0.18600076	0.71207729
C	4.14315180	2.42047245	-0.42250022
H	4.52310826	1.41566190	-0.57588888
C	-1.36996507	3.95564608	2.84258388
H	-1.61797062	4.34302463	3.82942665
C	2.32066810	-4.29664616	-1.37202542
H	3.33015950	-4.18709899	-0.96280046
H	1.72751092	-4.82206170	-0.60928036
C	-4.32690636	0.73253402	4.33488325
H	-4.70325040	1.08652629	5.29276142
C	6.05124270	-2.23049287	1.13865379
H	7.00879179	-1.97412028	1.60981486
H	6.21919171	-3.16574351	0.58170264
C	-1.56063990	4.07313757	0.44102678
H	-1.96367282	4.53597608	-0.45792741
C	2.24738194	-0.90686626	4.65378088
H	3.19446234	-1.09821244	5.15547677
C	0.30833012	-3.08386820	-2.27028915
H	-0.35961722	-3.57399170	-1.54532044
H	-0.11318487	-2.09123717	-2.48611165
C	-1.89619215	4.55797246	1.70299723
H	-2.56217760	5.41382918	1.79790782
C	4.98561798	-2.46520779	2.20126842
H	4.88632562	-1.55854359	2.81695352
H	5.28828751	-3.27160023	2.88204531
C	1.06030233	-1.39011661	5.19412978
H	1.07611021	-1.96789453	6.11658546
C	-5.54322614	-3.58262795	-1.76477591
H	-6.22376565	-4.24697189	-2.29388050
C	-4.27195424	-0.63554157	4.07364720
H	-4.60516819	-1.34987037	4.82399702
C	-5.12938396	4.16966979	-3.70085481
H	-6.15300714	4.27060437	-3.32385350
H	-5.19117442	3.88006647	-4.75847596
H	-4.66226921	5.16286190	-3.67019935
C	0.37171010	-3.91695396	-3.54225374

H	-0.63733884	-4.02930113	-3.96082746
H	0.96155663	-3.37268945	-4.29427411
C	2.37607070	-5.12653287	-2.64956189
H	3.05291588	-4.63555099	-3.36478308
H	2.81260058	-6.11009780	-2.43274503
C	-6.01304484	-2.80099406	-0.71515263
H	-7.06089929	-2.84858815	-0.42580998
C	-3.34000054	-2.66019001	-1.47230028
H	-2.29705364	-2.58304978	-1.77031286
C	-4.20589683	-3.50895835	-2.14649287
H	-3.83760614	-4.11012309	-2.97562805
C	1.00202296	-5.27775505	-3.28433636
H	0.35327589	-5.85484952	-2.60707895
H	1.07098078	-5.85376264	-4.21602286
C	4.86646234	5.95006370	-1.64099838
H	4.47014161	6.02629798	-2.66376326
H	5.95447799	5.84186388	-1.72596712
H	4.65829988	6.90237726	-1.14010339

Complex 7 (CyPOC(CH2Ph)CPNTol)Rh(PPh2NHTol)(Br)2

Rh	-0.04438156	0.13873801	-0.87230295
Br	1.43038903	-0.23370109	-3.06574070
Br	-2.06930655	0.61614800	-2.43174420
P	-0.58647355	-2.04469737	-0.56036809
P	-1.39161846	2.08649707	0.74419841
P	1.86929550	-0.05094261	0.44639542
O	-1.24839171	-1.96759473	0.98859967
N	-0.30381856	2.33491202	-0.43401297
N	2.99188390	-1.04399837	-0.33756857
H	2.71497195	-1.14349072	-1.31840866
C	0.65088968	-3.36719552	-0.32193873
H	1.50929581	-2.81269852	0.07119889
C	0.29238451	-4.44373071	0.70431788
H	-0.00251140	-3.97581206	1.65022111
H	-0.56917163	-5.02799627	0.35719809
C	1.48519667	-5.37228258	0.91209987
H	2.29939541	-4.81042430	1.39353955
H	1.21142002	-6.17447142	1.60994834
C	1.99800648	-5.95685680	-0.39788777
H	1.22849756	-6.62013809	-0.82230838
H	2.87996765	-6.58360978	-0.21343289
C	2.31984030	-4.86458022	-1.40949132
H	2.65456704	-5.30462314	-2.35725011
H	3.15340291	-4.24756547	-1.03898788
C	1.10784017	-3.97503171	-1.64807166
H	1.32895761	-3.18545879	-2.37913404
H	0.29616577	-4.58585657	-2.06699529
C	-2.01534329	-2.64562779	-1.52395225
H	-2.70216638	-1.79461045	-1.38257604
C	-2.70473354	-3.89645405	-0.98570289
H	-2.07320611	-4.77774239	-1.17314490
H	-2.86152943	-3.82367257	0.09751467
C	-4.03542519	-4.07347379	-1.70972751
H	-4.68628107	-3.22439779	-1.45447574

H	-4.54358755	-4.97460071	-1.34063973
C	-3.85203283	-4.13392600	-3.22102430
H	-4.82769366	-4.21757073	-3.71770644
H	-3.29538073	-5.04918622	-3.47734291
C	-3.08796898	-2.92390923	-3.74575807
H	-3.67830651	-2.00816544	-3.59426674
H	-2.92201783	-3.01382535	-4.82666785
C	-1.75520112	-2.75809109	-3.02829407
H	-1.12958878	-3.63636711	-3.23164813
H	-1.21505119	-1.87837667	-3.39591548
C	-1.24214184	0.33683600	0.72700914
C	-1.69071342	-0.73648244	1.38737569
C	-2.62989280	-0.81948932	2.55134005
C	-3.11400711	2.63197574	0.57205110
C	-3.47931836	3.93718647	0.91681054
H	-2.73687448	4.62922258	1.30907545
C	-4.79769709	4.35194276	0.77690310
H	-5.07400845	5.37118115	1.03866721
C	-5.76068317	3.46351145	0.30902386
H	-6.79413866	3.78860316	0.20439938
C	-5.40427123	2.16046130	-0.02330584
H	-6.15529837	1.46448921	-0.39190720
C	-4.08464953	1.74508698	0.09937546
H	-3.79227554	0.73951338	-0.19145741
C	-0.88700649	2.85225798	2.31665118
C	0.34587824	3.50051366	2.39112324
H	0.95725306	3.59859233	1.49531994
C	0.78226179	4.01767449	3.60731038
H	1.74183493	4.52718227	3.65758596
C	0.00062236	3.87724529	4.74788546
H	0.34848824	4.27692010	5.69861639
C	-1.23432128	3.23516574	4.67487585
H	-1.85279147	3.13663854	5.56489711
C	-1.68200299	2.73155032	3.46116927
H	-2.65896093	2.25421599	3.40005500
C	-0.12598711	3.47487999	-1.20910249
C	-0.33383205	4.77222693	-0.72150094
H	-0.68808274	4.92102532	0.29644612
C	-0.06881747	5.88478952	-1.51262185
H	-0.23830807	6.87920142	-1.09959922
C	0.41435368	5.75745376	-2.81311670
C	0.60684931	4.45910329	-3.29864226
H	0.96958559	4.31487430	-4.31631121
C	0.33510801	3.34225714	-2.52678754
H	0.47369889	2.34057992	-2.92765481
C	0.69196653	6.95562408	-3.67192198
H	-0.02446060	7.02857821	-4.50173325
H	1.69295604	6.90847767	-4.11950828
H	0.62490527	7.88516948	-3.09576923
C	1.66799556	-0.78097779	2.10192928
C	2.18344923	-2.04042819	2.41123398
H	2.79999507	-2.56218316	1.68356503
C	1.93160672	-2.61972267	3.64995250
H	2.33520728	-3.60551556	3.87353539
C	1.17693481	-1.93883266	4.59865811

H	0.98096572	-2.39236799	5.56842990
C	0.69245857	-0.66523556	4.31233903
H	0.12427854	-0.11119560	5.05748743
C	0.93373949	-0.09045115	3.07103565
H	0.55882222	0.90513446	2.85614451
C	2.67473697	1.55432263	0.71420209
C	3.24264499	1.96068660	1.92263872
H	3.20658154	1.31148976	2.79519124
C	3.84127216	3.21247203	2.01771168
H	4.28989833	3.52472639	2.95902845
C	3.85284307	4.06775817	0.91999822
H	4.29973943	5.05648877	1.00655381
C	3.29857773	3.65900502	-0.29082981
H	3.29023181	4.32354335	-1.15290775
C	2.73078203	2.39742830	-0.39868728
H	2.30139400	2.06648183	-1.34334219
C	4.37181557	-1.15941004	-0.13128343
C	4.99511936	-0.88400690	1.08623063
H	4.41389225	-0.53735063	1.93463157
C	6.36882610	-1.05451100	1.22446937
H	6.83021368	-0.82937712	2.18541402
C	7.16522103	-1.50287818	0.17393734
C	6.52799468	-1.77460411	-1.04122501
H	7.11652440	-2.12168085	-1.89003845
C	5.16318142	-1.60520698	-1.19818184
H	4.68968707	-1.80888325	-2.15775545
C	8.64646308	-1.68127497	0.32691456
H	9.20181122	-1.01579270	-0.34717854
H	8.95472196	-2.70757741	0.08884390
H	8.97075068	-1.46369321	1.35012601
H	-3.15592496	0.13714454	2.64039039
H	-2.03633092	-0.96223202	3.46558880
C	-3.60337507	-1.95731151	2.39093570
C	-4.75378429	-1.80308024	1.61745674
H	-4.98194621	-0.83002928	1.18564275
C	-5.61023448	-2.87676111	1.39855890
H	-6.50440766	-2.74021360	0.79329328
C	-5.32376861	-4.12187610	1.94796729
H	-5.99159294	-4.96335016	1.77424912
C	-4.18010080	-4.28391151	2.72385694
H	-3.94945312	-5.25465373	3.15884181
C	-3.32681336	-3.20841685	2.94245136
H	-2.42324558	-3.34038517	3.53644796

6. References

[1] Voskuil W. and Arens J. F., *Organic Syntheses, Coll.*, 1968, **48**, 47.

[2] P. Imhoff, R. van Asselt, C. J. Elsevier, K. Vrieze, K. Goubitz, K. F. van Malssen and C. H. Stam, *Phosphorus Sulfur Silicon Relat Elem*, 1990, **47**, 401–415.

[3] G. M. Sheldrick, *Acta Crystallogr. A*, 2008, **64**, 112–122.

[4] A. Thorn, B. Dittrich and G. M. Sheldrick, *Acta Crystallogr. A*, 2012, **68**, 448–451.

[5] G. M. Sheldrick, *Acta Crystallogr. Sect. C Struct. Chem.*, 2015, **71**, 3–8.

[6] M. J. Frisch, G. W. Trucks, H. B. Schlegel, G. E. Scuseria, M. A. Robb, J. R. Cheeseman, G. Scalmani, V. Barone, G. A. Petersson, H. Nakatsuji, X. Li, M. Caricato, A. v. Marenich, J. Bloino, B. G. Janesko, R. Gomperts, B. Mennucci, H. P. Hratchian, J. v. Ortiz, A. F. Izmaylov, J. L. Sonnenberg, D. Williams-Young, F. Ding, F. Lipparini, F. Egidi, J. Goings, B. Peng, A. Petrone, T. Henderson, D. Ranasinghe, V. G. Zakrzewski, J. Gao, N. Rega, G. Zheng, W. Liang, M. Hada, M. Ehara, K. Toyota, R. Fukuda, J. Hasegawa, M. Ishida, T. Nakajima, Y. Honda, O. Kitao, H. Nakai, T. Vreven, K. Throssell, Jr. Montgomery, J. A., J. E. Peralta, F. Ogliaro, M. J. Bearpark, J. J. Heyd, E. N. Brothers, K. N. Kudin, V. N. Staroverov, T. A. Keith, R. Kobayashi, J. Normand, K. Raghavachari, A. P. Rendell, J. C. Burant, S. S. Iyengar, J. Tomasi, M. Cossi, J. M. Millam, M. Klene, C. Adamo, R. Cammi, J. W. Ochterski, R. L. Martin, K. Morokuma, O. Farkas, J. B. Foresman and D. J. Fox, Gaussian, Inc., Wallingford CT, 2016.

[7] T. Lu and F. Chen, *J Comput Chem*, 2012, **33**, 580–592.

[8] Y. Zhao and D. G. Truhlar, *J Phys Chem A*, 2005, **109**, 5656–5667.

[9] L. Falivene, Z. Cao, A. Petta, L. Serra, A. Poater, R. Oliva, V. Scarano and L. Cavallo, *Nat Chem*, 2019, **11**, 872–879.
